# ‘Omics‐guided prediction of the pathway for metabolism of isoprene by *Variovorax* sp. WS11

**DOI:** 10.1111/1462-2920.16149

**Published:** 2022-08-05

**Authors:** Robin A. Dawson, Gregory D. Rix, Andrew T. Crombie, J. Colin Murrell

**Affiliations:** ^1^ School of Environmental Science University of East Anglia, Norwich Research Park Norwich UK

## Abstract

Bacteria that inhabit soils and the leaves of trees partially mitigate the release of the abundant volatile organic compound, isoprene (2‐methyl‐1,3‐butadiene). While the initial steps of isoprene metabolism were identified in *Rhodococcus* sp. AD45 two decades ago, the isoprene metabolic pathway still remains largely undefined. Limited understanding of the functions of *isoG*, *isoJ* and *aldH* and uncertainty in the route of isoprene‐derived carbon into central metabolism have hindered our understanding of isoprene metabolism. These previously uncharacterised *iso* genes are essential in *Variovorax* sp. WS11, determined by targeted mutagenesis. Using combined ‘omics‐based approaches, we propose the complete isoprene metabolic pathway. Isoprene is converted to propionyl‐CoA, which is assimilated by the chromosomally encoded methylmalonyl‐CoA pathway, requiring biotin and vitamin B12, with the plasmid‐encoded methylcitrate pathway potentially providing robustness against limitations in these vitamins. Key components of this pathway were induced by both isoprene and its initial oxidation product, epoxyisoprene, the principal inducer of isoprene metabolism in both *Variovorax* sp. WS11 and *Rhodococcus* sp. AD45. Analysis of the genomes of distinct isoprene‐degrading bacteria indicated that all of the genetic components of the methylcitrate and methylmalonyl‐CoA pathways are not always present in isoprene degraders, although incorporation of isoprene‐derived carbon via propionyl‐CoA and acetyl‐CoA is universally indicated.

## INTRODUCTION

Annual emissions of isoprene (2‐methyl‐1,3‐butadiene) to the atmosphere rival those of methane, with the vast majority of isoprene coming from biogenic sources including terrestrial plants, particularly trees, which contribute approximately 90% of the estimated total 500 Tg isoprene yr^−1^ (Guenther et al., 2012; Pacifico et al., 2012), dwarfing the contributions of aquatic ecosystems which range from 0.1 to 11.6 Tg isoprene yr^−1^ in marine environments, reviewed by Dawson et al. (2021). Anthropogenic sources of isoprene primarily originate from vehicular exhaust (Khan et al., 2018; Reimann et al., 2000; Wang et al., 2013) and the industrial production of polyisoprene rubber, which contributes an estimated 0.8 Tg isoprene yr^−1^ (Greve, 2000). Studies have identified that isoprene, a highly reactive dialkene, reacts with and depletes hydroxyl radicals in the atmosphere, thus lengthening the residence time of methane and contributing to global warming (Atkinson & Arey, 2003; Carlton et al., 2009; Pacifico et al., 2009, 2012). Isoprene also influences cloud formation, and therefore climate, through secondary organic aerosol production (Carlton et al., 2009; Hu et al., 2013; Meskhidze & Nenes, 2007), and can negatively impact air quality by producing ozone in industrialized areas due to interactions with NO_x_ (Atkinson & Arey, 2003; Fiore et al., 2012; Trainer et al., 1987).

Soils are a relatively small but significant sink for isoprene, with rates of uptake of 20.4 Tg C yr^−1^ in early estimates (Cleveland & Yavitt, 1997). Isoprene consumption by soils was linked to microbial activity (Cleveland & Yavitt, 1997, 1998), with both bacterial and fungal communities potentially contributing (Gray et al., 2015), likely by consuming isoprene produced by soil‐borne isoprene emitters. The diversity of isoprene‐degrading communities has been investigated through cultivation‐dependent and ‐independent techniques, with deoxyribonucleic acid stable isotope probing (DNA‐SIP) serving to identify the active isoprene‐degrading genera in soils, or associated with the phyllosphere of temperate and tropical trees (Carrión et al., 2018; Crombie et al., 2018; Gibson et al., 2021; Larke‐Mejía et al., 2019). *Rhodococcus* sp. AD45, isolated from freshwater sediment, was one of the first isoprene degraders to be characterized. Studies by van Hylckama Vlieg et al. (1998, 1999, 2000) reported a partial metabolic pathway for the degradation of isoprene, and subsequent sequencing and analysis of the *Rhodococcus* sp. AD45 genome and transcriptome identified a cluster of genes presumed to be involved in isoprene metabolism (Crombie et al., 2015). The genes encoding this metabolic pathway (see below) were subsequently identified in other cultivated isoprene degraders and also in the metagenome‐assembled genomes derived from isoprene‐enriched communities (Carrión et al., 2020; Crombie et al., 2018; Dawson et al., 2020; Larke‐Mejía et al., 2019). The Comamonadaceae family (which includes *Variovorax*) were abundant components of isoprene‐degrading communities, including from soil from a tyre dump, soils taken from the vicinity of willow and oil palm trees, and the phyllospheres of willow and poplar trees (Carrión et al., 2020; Crombie et al., 2018; Gibson et al., 2021; Larke‐Mejía et al., 2019), indicating that *Variovorax* species may be significant contributors to the consumption of isoprene in these environments.

All isoprene‐degrading bacteria characterized thus far employ the first steps of isoprene oxidation identified in *Rhodococcus* sp. AD45 (Crombie et al., 2015; Dawson et al., 2020; Gibson et al., 2020; Larke‐Mejía et al., 2020; van Hylckama Vlieg et al., 2000). Isoprene monooxygenase (IsoMO, encoded by *isoABCDEF*) catalyses the initial oxidation of isoprene to epoxyisoprene (Crombie et al., 2015; Dawson et al., 2020; Sims et al., 2022; van Hylckama Vlieg et al., 2000). An isoprene pathway‐specific glutathione *S*‐transferase, IsoI, then conjugates epoxyisoprene with glutathione, a thiol not typically found in Gram‐positive bacteria (Johnson et al., 2009), which was also implicated in the mechanistically similar styrene metabolic pathway of *Gordonia rubripertincta* CWB2 (Heine et al., 2018), forming 1‐hydroxy‐2‐glutathionyl‐2‐methyl‐3‐butene (HGMB) (van Hylckama Vlieg et al., 1999). IsoH, a NAD^+^‐dependent dehydrogenase, then catalyses two successive oxidation reactions, first from HGMB to 1‐oxo‐2‐glutathionyl‐2‐methyl‐3‐butene (GMB), then from GMB to 2‐glutathionyl‐2‐methyl‐3‐butenoic acid (GMBA). The structures of these intermediate metabolites are shown in Figure S1. In *Rhodococcus* sp. AD45, van Hylckama Vlieg et al. (2000) predicted the incorporation of carbon from isoprene via β‐oxidation, resembling fatty acid metabolism (Jimenez‐Diaz et al., 2017). This is likely initiated with the incorporation of a coenzyme A (CoA) group by the predicted CoA‐transferase IsoG to form a CoA‐thioester. Additional conserved *iso* metabolic genes, *isoJ* (a second glutathione *S‐*transferase) and *aldH* (a putative dehydrogenase), also lack functional characterizations. A possible role of AldH was suggested during a study of the styrene metabolic pathway of *G. rubripertincta* CWB2, wherein AldH may perform the same second oxidation reaction as StyH, which catalyses the oxidation of (1‐phenyl‐2‐acetaldehyde)glutathione to (1‐phenyl‐2‐acetic acid)glutathione (Heine et al., 2018). The equivalent reaction in the isoprene metabolic pathway is the oxidation of GMB to GMBA by IsoH (van Hylckama Vlieg et al., 1999), although there is currently no evidence to support this role for AldH in isoprene metabolism. The entire 22‐gene *iso* metabolic gene cluster in *Rhodococcus* sp. AD45 was transcribed during growth on isoprene or upon induction with epoxyisoprene, suggesting the involvement of these uncharacterized genes, including glutathione biosynthesis genes (*gshAB*) and two phylogenetically distinct *aldH* genes. However, genes involved in the methylcitrate cycle and methylmalonyl‐CoA pathway, which were predicted to be involved if isoprene were incorporated by a similar mechanism as odd‐chain fatty acids (Textor et al., 1997), were not differentially expressed in the presence of isoprene (Crombie et al., 2015).

The impact of bacteria in mitigating global emissions of isoprene remains to be fully characterized, and the exact mechanism by which bacteria assimilate carbon from isoprene is still unclear. Until now, the only detailed mechanistic information on the isoprene metabolic pathway was derived from a single bacterium, *Rhodococcus* sp. AD45. This study aimed to provide further insights into the isoprene metabolic pathway using an environmentally abundant and genetically tractable Gram negative isoprene degrader, *Variovorax* sp. WS11. This study involved a combination of ‘omics‐based approaches, with further molecular characterization of candidate genes suspected to be involved in isoprene metabolism. By comparing these data from *Variovorax* sp. WS11 with those already gathered from *Rhodococcus* sp. AD45, we hypothesize a generalized metabolic pathway, which facilitates the oxidative breakdown of isoprene and incorporation of isoprene‐derived carbon into central metabolism. We also confirm that previously uncharacterized *iso* metabolic genes are essential for isoprene metabolism, considerably advancing our knowledge of this environmentally important biogeochemical process.

## EXPERIMENTAL PROCEDURES

### Culture conditions

*Variovorax* sp. WS11 was cultivated in liquid medium, herein Ewer's medium, as described by Dawson et al. (2020). Isoprene concentrations in the headspace of vials were measured using a Fast Isoprene Sensor (Hills Scientific, Boulder, Colorado) as described previously (Dawson et al., 2020).

### Analysis of the transcriptome of isoprene‐grown *Variovorax* sp. WS11

The transcriptome of isoprene‐grown *Variovorax* sp. WS11 was prepared and analysed as described in the Supporting Information Materials and Methods S1. In brief, succinate‐grown *Variovorax* sp. WS11 cells were washed in Ewers medium and starved for 1 h to deplete intracellular carbon stores (Timepoint 0). These cultures were switched to one of four conditions (1% (v/v) isoprene, 0.01% (w/v) epoxyisoprene, 10 mM succinate, no substrate), and 10 ml aliquots were harvested at specific intervals and stored for RNA extraction (see Supporting Information S1). Total RNA was sent for library preparation and transcriptome sequencing by Novogene UK (Cambridge, UK) using an Illumina NovaSeq 6000 instrument.

### Analysis of the proteome of isoprene‐grown *Variovorax* sp. WS11

The proteome of isoprene‐grown *Variovorax* sp. WS11 was prepared and analysed as described in the Supporting Information Materials and Methods. Succinate‐grown *Variovorax* sp. WS11 was washed in Ewers medium, cells were starved to deplete intracellular stores of carbon (Timepoint 0), and switched onto one of two growth conditions (10 mM succinate or 1% (v/v) isoprene). The 200 ml aliquots were removed at Timepoint 0, after 6, 24, or 30 h and the total protein content was extracted. Fractionation, Tandem Mass Tagging, and proteome analysis were run at the Proteomics Facility (John Innes Centre, Norwich, UK) using nanoLC‐MS/MS on an Orbitrap Eclipse™ Tribrid™ mass spectrometer, coupled to an UltiMate® 3000 RSLCnano LC system (Thermo Fisher Scientific, Hemmel Hempstead, UK). Data processing was run using Proteome Discoverer 2.4.1.14 (Thermo). A total of 5364 proteins were identified, of which 4350 were quantified with at least two unique peptides. Hypothesis testing was run using the background‐based *t*‐test (see Supporting Information Materials and Methods S1).

### Deletion of *iso* genes by targeted mutagenesis and restoration of function by complementation

*isoG*, *isoJ*, *aldH* and *garB* were selected for deletion by double‐homologous recombination (Schäfer et al., 1994). Suicide constructs (Table S4) were constructed using the primers described in Table S5. Targeted *iso* gene deletions were conducted as detailed by Dawson et al. (2020). Approximately 500 bp flanking regions upstream and downstream of the gene of interest, overlapping the translational start and stop codons, were amplified by polymerase chain reaction (PCR) using Q5 high‐fidelity polymerase (NEB, Hitchin, UK) and ligated into pK18mobsacB (Schäfer et al., 1994). The gentamicin resistance gene (*aacC1*) was PCR‐amplified from pCM351 to include the 5′ and 3′ *loxP* sites (Marx & Lidstrom, 2002) (Table S5). *aacC1*‐*loxP* was inserted between the flanking regions cloned into pK18mobsacB, forming the mutagenic vectors described in Table S4. *Variovorax* sp. WS11 was transformed by electroporation, and the mutagenic vector backbones were subsequently removed by counter‐selection using sucrose (Dawson et al., 2020). Transformation of targeted mutants with a Cre recombinase‐encoding vector, pCM158 (Marx & Lidstrom, 2002), catalysed the excision of *aacC1* and formed marker‐free mutants. Restoration of function was achieved by synthesizing intact copies of the deleted genes by PCR using Q5 high‐fidelity polymerase and subsequently ligating into pBBR1MCS‐2 under the control of the de‐repressed *lacZ* promoter (Kovach et al., 1995). Each marker‐free mutant was transformed using the respective complementation vector by electroporation (Dawson et al., 2020), and restored gene function was confirmed by inoculating the complemented mutants in Ewers medium with 1% (v/v) isoprene (Figures [Link], [Link]). Consumption of isoprene from the headspace of 120 ml vials was measured by fast isoprene sensor.

### RT‐qPCR‐based analysis of gene expression by *Variovorax* sp. WS11

*Variovorax* sp. WS11 and *Variovorax* sp. WS11 Δ*garB* were grown in 50 ml Ewers medium with 10 mM succinate or 1% (v/v) isoprene to an OD_540_ of 0.6, using three biological replicates for each condition. Cells were pelleted by centrifugation at 4000*g* for 10 min at 4°C. Total RNA was extracted using TRIzol reagent (ThermoFisher Scientific, Loughborough, UK) according to the manufacturer's instructions. Contaminating DNA was removed by a single treatment with RNase‐free DNase (Qiagen, Manchester, UK), then DNase‐treated samples were purified using RNeasy spin columns (RNeasy Mini Kit, Qiagen). RNA concentration was determined using a Qubit RNA Broad Range assay kit (ThermoFisher Scientific), and a lack of contaminating DNA was verified by a negative PCR assay using 16S rRNA primers. cDNA was generated from approximately 900 ng RNA in 20 μl reactions using the Superscript III reverse transcriptase kit (ThermoFisher Scientific) with random hexamers (Invitrogen), including controls for each condition without reverse transcriptase for reference. Primers were designed to amplify *garB_1*, *garB_2*, and *garB_3*, using *rpoB* as an internal reference gene (Dawson et al., 2020) (Table S5). Standard curve‐based reverse transcriptase quantitative polymerase chain reaction (RT‐qPCR) was conducted in 20 μl reactions using SensiFAST SYBR HI‐ROX master mix (Bioline, London, UK), with 2 μl of cDNA prepared at a 1/20 dilution. qPCR was performed using a StepOnePlus instrument (Applied Biosystems, Waltham, USA). Target gene transcription was normalized to *rpoB* and presented as the level of expression during growth on isoprene compared to growth on succinate.

### Measurement of isoprene uptake by *Variovorax* sp. WS11

Isoprene uptake assays were conducted as described by Dawson et al. (2020). Variovorax sp. WS11 was grown in 20 ml Ewers medium in 120 ml vials at 30°C, with shaking at 160 rpm, to an OD_540_ of 0.6 in sealed with butyl rubber stoppers and aluminium crimp caps, using 1% (v/v) isoprene, 10 mM pyruvate, or a combination of 10 mM pyruvate and 1% (v/v) isoprene. Cells were pelleted by centrifugation at 4000*g* for 10 min at 4°C, then resuspended to an OD_540_ of 10.0 in 1 ml of ice cold 4‐(2‐hydroxyethyl)‐1‐piperazineethanesulfonic acid (HEPES) (50 mM, pH 6.0). Cell suspensions were sealed in 30 ml vials and pre‐incubated for 3 min in a water bath at 30°C with shaking at 160 rpm, then spiked with 1 ml of headspace from a 1% isoprene stock. After a further 1 min, 50 μl headspace samples were taken using a gas‐tight syringe (Agilent, Santa Clara, CA, USA) and injected into a Fast Isoprene Sensor. Further samples were taken every 3 min for 1 h.

### BLAST analysis of gene homologues and predicted protein domains

Translated amino acid sequences were used as query sequences against a specific subject genome sequence using tBLASTn (Altschul et al., 1990) in order to identify genes with homology to the query sequence. Standard parameters were used in each case. Predicted protein domains were analysed by BLASTp analysis, with the translated amino acid sequence used as a query against the standard non‐redundant protein sequences (nr) database (Altschul et al., 1990).

### Statistical analysis

All experiments were conducted using three biological replicates for each condition, with the exception of the proteome study, which was run using two biological replicates per condition. Statistical differences between two experimental conditions were calculated using the Student's *t‐*test. Statistical differences between more than two experimental conditions were initially calculated using one‐way ANOVA, followed by post hoc testing using Tukey's HSD test (calculated by Statistics Kingdom, accessed at https://www.statskingdom.com/180Anova1way.html).

## RESULTS AND DISCUSSION

### Multi ‘omics analysis of isoprene metabolism by *Variovorax* sp. WS11

**FIGURE 1:**
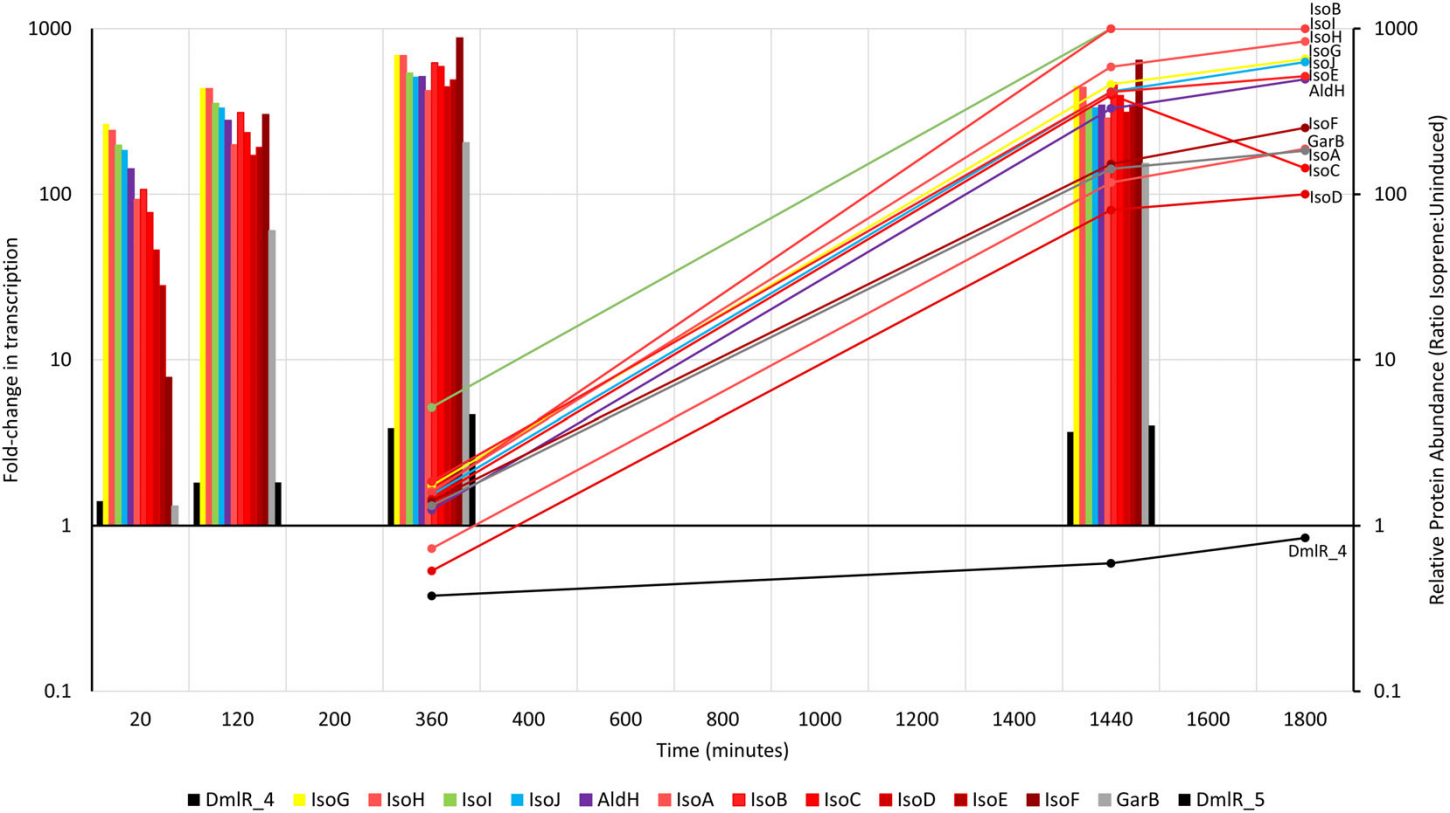
Transcription of the *iso* gene cluster during growth on isoprene (bars = fold‐increase in transcription) and abundance of the Iso proteins (lines = relative protein abundance over time, relative to Timepoint 0). Each colour corresponds to a specific *iso* metabolic gene (see Figure S2A), with transcriptome data shown in the 5–3′ gene order. Proteome data are labelled with the appropriate Iso protein. *Y* axes are presented on a Log(10) scale

**FIGURE 2:**
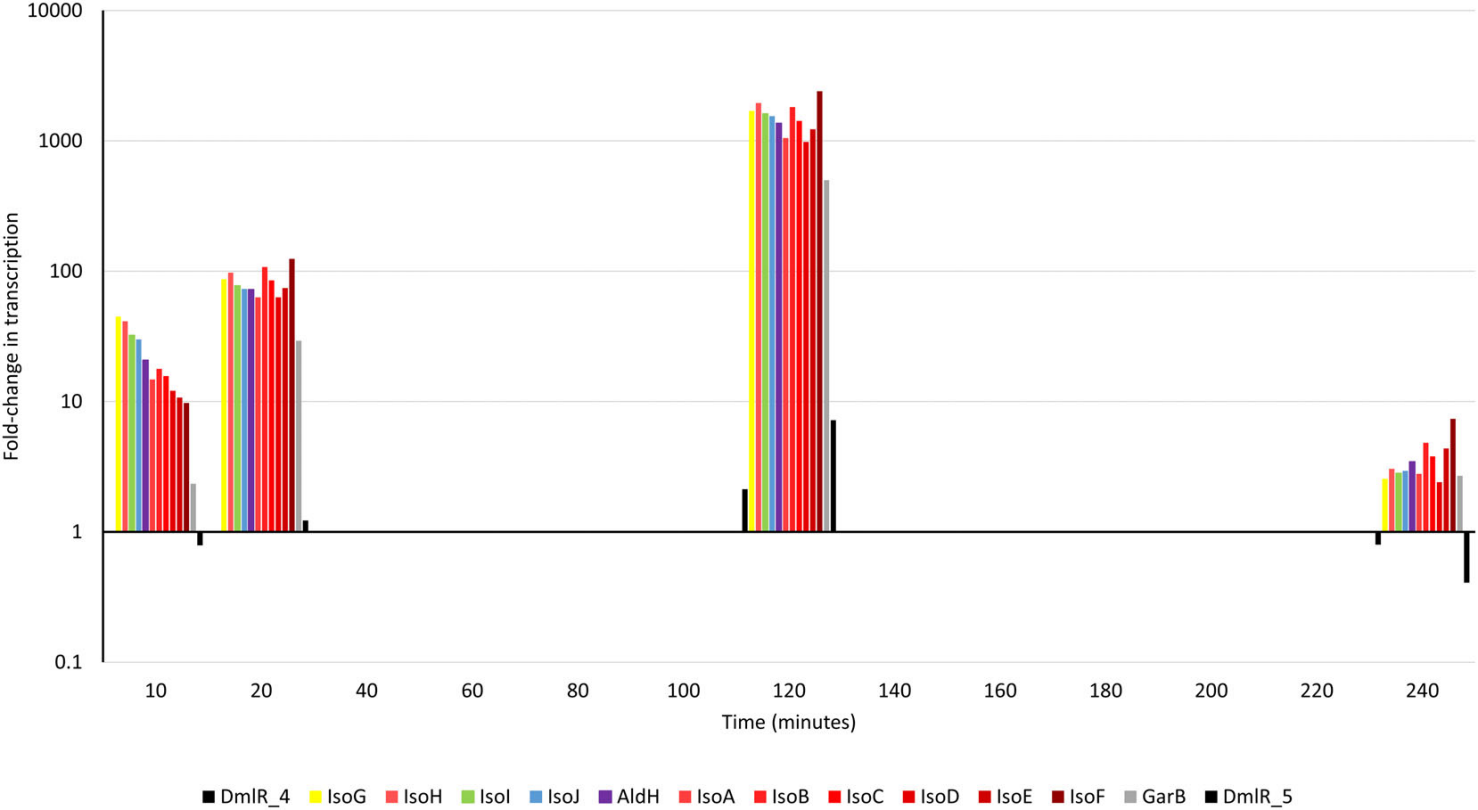
Transcription of the *iso* gene cluster during induction by epoxyisoprene (bars = fold‐change in transcription), relative to Timepoint 0. Each colour corresponds to a specific *iso* metabolic gene (see Figure S2A), with data presented in the relevant 5′–3′ gene order. *Y* axes are presented on a Log(10) scale.

To confirm that the isoprene metabolic genes (Figure S2A) were induced by isoprene, we investigated the effect of incubation of succinate‐grown cell suspensions with either isoprene, epoxyisoprene, succinate, or no‐substrate, on the *Variovorax* sp. WS11 transcriptome over a range of timepoints (see Experimental Procedures). Alongside this transcriptomic analysis, we also analysed the proteome of cells in a parallel experiment in which succinate‐grown cells were incubated with either isoprene or succinate with samples taken at 0, 6, 24, and 30 h after adding the growth substrate. Both isoprene and epoxyisoprene induced large changes in gene transcription by *Variovorax* sp. WS11. Sequences at the 5′ end of the *iso* gene cluster were more abundant in the RNAseq data than at the 3′ end at Timepoint 1 (20 min), possibly due to the fact that termination of transcription often occurs at the 5′ end of the sequence. Transcription of the *iso* genes (Figure S2A) accounted for 8.5% of normalized transcripts [fragments per kilobase million (FKPM)] after 6 h in the presence of isoprene, with 267‐ to 8‐fold upregulation of *iso* transcripts relating to *isoG* and *isoF*, respectively, at Timepoint 1 (20 min), increasing to 451‐ to 694‐fold at Timepoint 4 (24 h), relative to Timepoint 0 (0 h) (Figure 1, Figure S3), although the relative transcript abundance had decreased slightly to 5.8% of transcripts by Timepoint 4 (24 h). The decrease in relative abundance of *iso* gene transcripts may have been due to decreasing levels of isoprene in the headspace of the vials during incubations, due to increasing transcription of genes involved in metabolic processes associated with the incorporation of carbon from isoprene other than the *iso* gene cluster, thereby decreasing the relative FKPM of *iso*‐derived transcripts, or due to fewer transcripts being required to maintain the translated Iso proteins at a high level compared to the initial stages of incubation with isoprene. In the presence of epoxyisoprene, transcription of the *iso* metabolic genes increased by a fold‐change relative to Timepoint 0 of 10–45 after 10 min, and 983‐ to 2406‐fold after 2 h (Figure 2). Transcripts specific to the *iso* gene cluster accounted for 22.5% of transcripts after 2 h of incubation with epoxyisoprene (Figure S3). The more rapid induction of *iso* gene expression by epoxyisoprene supports the hypothesis that epoxyisoprene, or a subsequent intermediate of isoprene metabolism, is the primary inducer of *iso* gene expression as shown in *Rhodococcus* sp. AD45 (Crombie et al., 2015). However, transcription of the *isoG* gene in an *isoA* deletion mutant of *Variovorax* sp. WS11 (Δ*isoA*, deficient for the α‐oxygenase subunit of isoprene monooxygenase) exhibited a weak response to isoprene (Dawson et al., 2020). This confirmed that, although epoxyisoprene is the more potent inducer of *iso* gene expression, isoprene is also an inducer. The relative transcription of the *iso* cluster during induction with epoxyisoprene decreased from 22.5% after 2 h to 0.1% after 4 h (Figure 2). The decrease in gene induction was likely to be caused by the loss of epoxyisoprene due to hydrolysis in water to the corresponding diol (Golding et al., 2022), rather than metabolism of epoxyisoprene to HGMB by the isoprene metabolic pathway. The abundances of Iso proteins at Timepoint 0 (0 h) accounted for 0.00001%–0.005% of the total proteome, compared 0.005%–3.32% of the total proteome after 30 h of growth on isoprene (Supplementary Data sheet S2, L4501‐4514), making it unlikely that IsoI was present at sufficient abundance to catalyse the complete conversion of epoxyisoprene to HGMB within the initial 2–4 h period. However, this cannot be definitively stated without available proteome data after 2 h in the presence of isoprene. Succinate failed to induce a fold‐increase in *iso* gene expression beyond 0 and 3, when compared to Timepoint 0 (0 h) (Supplementary Data Sheet S1, L638‐649), confirming previous observations that isoprene metabolism is specifically induced by isoprene or epoxyisoprene (Crombie et al., 2015; Dawson et al., 2020).

In contrast to the rapid transcriptional response of *Variovorax* sp. WS11 to isoprene, the corresponding Iso proteins were not significantly expressed above Timepoint 0 (0 h) levels after 6 h of growth on isoprene, with the exception of IsoI (*p* ≤ 0.01) (Figure 1). This provides an interesting perspective into the physiology of isoprene degraders during the early stages of isoprene metabolism. An isoprene‐degrading bacterium would be protected from toxic accumulation of epoxyisoprene by initially producing greater quantities of IsoI than the IsoMO proteins. The ratio of abundance of Iso proteins when *Variovorax* sp. WS11 was grown on succinate, compared to Timepoint 0 (0 h), largely failed to exceed 1.0, with the greatest positive increase demonstrated by IsoI with a ratio of 2.5 after 24 h of growth on succinate (Supplementary Data Sheet S2, L4512). The significant increases in expression of IsoG, IsoJ, AldH and GarB in the proteome of isoprene‐grown *Variovorax* sp. WS11 indicated that these previously uncharacterized *iso* metabolic proteins may be required during isoprene metabolism. *aldH*, encoding a putative dehydrogenase, has consistently been identified in the *iso* gene clusters of extant and metagenome‐derived isoprene degraders (Carrión et al., 2020; Crombie et al., 2015, 2018; Dawson et al., 2020; Gibson et al., 2020; Larke‐Mejía et al., 2019). Two phylogenetically distinct *aldH* genes were significantly expressed in *Rhodococcus* sp. AD45 during growth on isoprene (Crombie et al., 2015). *Variovorax* sp. WS11 contains a single copy of *aldH* (Dawson et al., 2020), sharing the greatest predicted amino acid identity with AldH_1 from *Rhodococcus* sp. AD45 (52.7%, compared to 26.1% identity with AldH_2). The transcription of *aldH* increased significantly from a fold‐change of 143 after 20 min to 514 after 6 h in the presence of isoprene, relative to Timepoint 0 (0 h) (Figure 1). This change was reflected in the proteome of *Variovorax* sp. WS11, as the relative abundance of AldH increased from 1.25 after 6 h to 494.7 after 30 h of growth on isoprene, relative to Timepoint 0 (0 h). An AldH homologue in the isoprene‐degrading *Rhodococcus opacus* PD630, which shares the same genetic context with AldH1 from *Rhodococcus* sp. AD45 and shares 90% amino acid identity with the latter, was characterized as a NAD(P)^+^‐dependent glyceraldehyde 3‐phosphate dehydrogenase (MacEachran & Sinskey, 2013).

Transcription of the putative glutathione‐disulfide reductase, *garB*, increased significantly to a maximum fold‐change of 205 after 6 h of growth on isoprene, and the translated protein increased in abundance to 183.2, relative to Timepoint 0 (0 h) (Figure 1), providing the first evidence that *garB* might be required for growth on isoprene. Glutathione disulfide reductase (GarB) catalyses the reductive cleavage of the disulfide bond of oxidized glutathione‐disulfide (GSSG) to form reduced glutathione (GSH) in order to restore its redox function (Deponte, 2013; Fuchs, 1977). *garB* has only been identified in the *iso* gene clusters of Gram negative isoprene degraders (Carrión et al., 2020; Dawson et al., 2020), raising the question of how Gram positive isoprene degraders cycle redox‐active species, particularly glutathione. The isoprene metabolic gene cluster of *Rhodococcus* sp. AD45 contains a putative CoA‐disulfide reductase, which shares only 26.9% identity at the predicted amino acid level with GarB from *Variovorax* sp. WS11. The consistent requirement for glutathione by Gram positive and Gram negative isoprene degraders, indicated by the conservation of *isoI*, suggests that the putative CoA‐disulfide reductase in the *Rhodococcus* sp. AD45 *iso* gene cluster may, in fact, act as a glutathione‐disulfide reductase despite the low sequence identity. Experimental validation of the roles of each thiol reductase will be required in the future. GarB from *Variovorax* sp. WS11 shares 51.5% identity with the characterized glutathione reductase from *E. coli* K‐12 (EC:1.8.1.7), suggesting that the functional prediction as a glutathione‐disulfide reductase is correct. The putative LysR‐type transcriptional regulator (LTTR) *dmlR_4* has consistently been identified upstream of *isoG* in the *iso* metabolic gene clusters from known Gram negative isoprene degraders (Carrión et al., 2020; Dawson et al., 2020; Larke‐Mejía et al., 2019), while Gram positive isoprene degraders typically contain MarR‐type transcriptional regulators at various positions upstream (5′) of *isoG* (Crombie et al., 2015; Murrell et al., 2020). The relative abundance of DmlR_4 did not change significantly during growth on isoprene, and the transcription of *dmlR_4* only increased by a fold‐change of 4 after 6 h on isoprene (Figure 1). The second LTTR present in the *iso* metabolic gene cluster of *Variovorax* sp. WS11, *dmlR_5* (Dawson et al., 2020), was not detected in the proteome of succinate‐grown or isoprene‐grown *Variovorax* sp. WS11, indicating that this LTTR was only expressed at a very low abundance. However, transcription of *dmlR_5* increased by a fold‐change of 5 after 6 h of growth on isoprene, and was maintained at a fold‐increase of 4 after 24 h of growth, relative to Timepoint 0 (0 h) (Figure 1). Likewise, the transcription of each putative LTTR increased when *Variovorax* sp. WS11 was incubated in the presence of epoxyisoprene (Figure 2).

### Molecular analysis and the roles of previously uncharacterized *iso* genes

**FIGURE 3:**
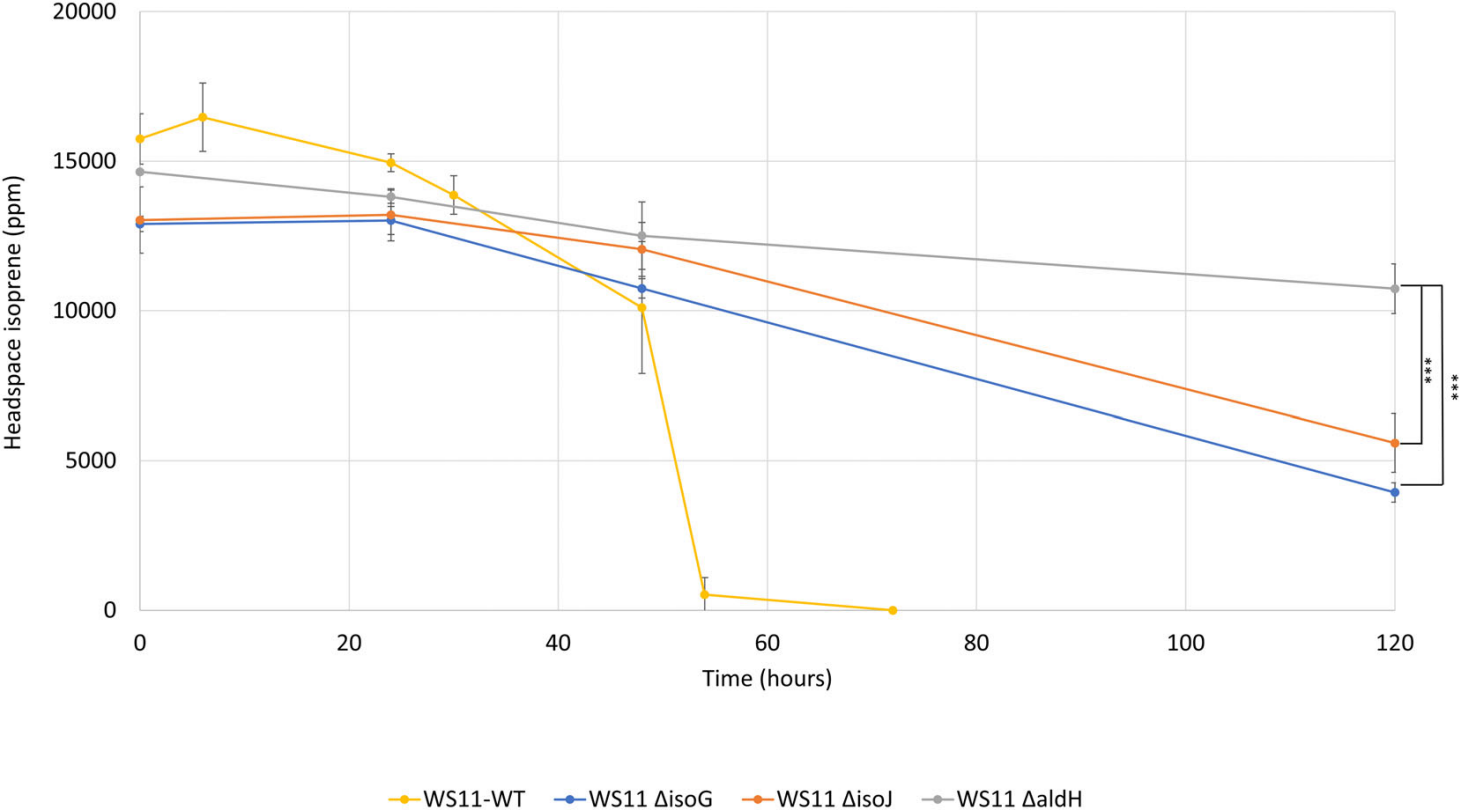
Consumption of isoprene by wild‐type *Variovorax* sp. WS11 compared with targeted *iso* mutants; *Variovorax* sp. WS11 Δ*isoG*, Δ*isoJ*, and Δ*aldH*, during growth on a combination of 10 mM pyruvate and ~1.5% (v/v) isoprene. Error bars standard deviation about the mean of three biological replicates. An asterisk denotes a statistically significant difference between the indicated conditions (***p ≤ 0.0005), determined by Tukey's HSD test.

To confirm the involvement in isoprene metabolism of uncharacterized isoprene metabolic genes, *isoG*, *isoJ*, *aldH*, and *garB*, which were highly upregulated by isoprene (Figure 1), these candidates were targets for deletion by marker‐exchange mutagenesis. IsoJ was partially characterized as a GST in *Rhodococcus* sp. AD45 with confirmed activity towards 1‐chloro‐2,4‐dinotrobenzene (CDNB) and 3,4‐dichloro‐1‐nitrobenzene (DCNB) (van Hylckama Vlieg et al., 2000), commercially available substrates for this family of enzymes. IsoJ lacked activity towards HGMB and GMBA (van Hylckama Vlieg et al., 2000), suggesting that this GST may have activity instead towards a downstream metabolic intermediate of isoprene metabolism. IsoG and AldH are putative CoA‐transferase and dehydrogenase enzymes, respectively, with an unknown role in isoprene metabolism. *Variovorax* sp. WS11 strains with disrupted *isoG* (Δ*isoG*), *isoJ* (Δ*isoJ*) or *aldH* (Δ*aldH*) were unable to grow on isoprene, although *Variovorax* sp. WS11 Δ*isoJ* and Δ*aldH* mutants consumed a small amount of isoprene from the headspace of the respective vials (Figures [Link], [Link]). Although oxidation of isoprene is repressed in cultures of *Variovorax* sp. WS11 exposed to both isoprene and glucose (Dawson et al., 2020), we noted that pyruvate in combination with isoprene did not have a similar repressing effect on isoprene oxidation (Figure S7). Therefore, each mutant strain was supplied with a combination of 1% (v/v) isoprene and 10 mM pyruvate. *Variovorax* sp. WS11 Δ*isoG*, Δ*isoJ*, and Δ*aldH* mutants consumed isoprene from the headspace of sealed 120 ml vials when pyruvate was present to support growth and to supply the reducing power required for isoprene oxidation (Figure 3), albeit at a significantly lower rate than the wild‐type strain (*p* ≤ 0.05, determined by Tukey's HSD test), which consumed all detectable isoprene within 72 h. The *Variovorax* sp. WS11 Δ*aldH* mutant consumed significantly less isoprene than mutants WS11 Δ*isoG* and WS11 Δ*isoJ* after 120 h (*p* ≤ 0.0005) (Figure 3). The lack of growth by the mutants with isoprene as the sole carbon source confirmed that an essential component of the isoprene metabolic pathway was disrupted, but the ability to grow and consume isoprene when additional pyruvate was supplied shows that the accumulated metabolic intermediates were not toxic to *Variovorax* sp. WS11, or at least were not retained within the cell at a toxic concentration. When intact copies of *isoG*, *isoJ* and *aldH* were restored to the respective *Variovorax* sp. WS11 mutants on plasmids, growth on isoprene was restored (Figures [Link], [Link]), albeit to a lesser degree than in the wild type in the case of the complemented strains of *Variovorax* sp. WS11 Δ*isoJ* (*p* ≤ 0.01) and Δ*aldH* (*p* ≤ 0.01). Each wild‐type copy of the deleted genes was expressed from the de‐repressed *lac* promoter of pBBR1MCS‐2 (Kovach et al., 1995), resulting in a constitutive low level of expression of the cloned gene rather than the high level of gene expression observed during the growth of wild‐type *Variovorax* sp. WS11 on isoprene (Figure 1).

Crombie et al. (2015) reported that the functionally characterized protein with the greatest similarity to IsoG (from *Rhodococcus* sp. AD45) was a succinyl‐CoA:d‐citramalate CoA transferase from *Chloroflexus aurantiacus*. The deduced IsoG amino acid sequence from *Variovorax* sp. WS11 encodes conserved domains which indicated CoA‐transferase activity, as determined by BLASTp analysis. If isoprene is incorporated into central metabolism via β‐oxidation, it stands to reason that a CoA group would be incorporated into the isoprene‐derived carbon chain. A GMBA‐CoA conjugate (2‐glutathionyl‐2‐methyl‐3‐butenyl‐CoA) is the most likely intermediate compound, since the alternative, removal of glutathione by glutathione‐*S*‐transferase IsoJ before CoA addition, is not supported due to the lack of activity towards GMBA by IsoJ reported by van Hylckama Vlieg et al. (2000). More likely, IsoJ catalyses the reductive removal of GSSG from GMBA‐CoA conjugates. GSSG may then be reduced to two molecules of GSH by GarB, allowing the continued detoxification of epoxyisoprene by IsoI. However, despite the high levels of gene and protein expression resulting from exposure to isoprene, *garB* was not essential for growth of *Variovorax* sp. WS11 on isoprene (Figure S8). While the megaplasmid‐encoded *iso* gene cluster contains one copy of *garB* (*garB_3*), two additional copies of *garB* (*garB_1*, *garB_2*) were detected on the chromosome with 45.3% and 42.9% identity to *garB*_3 from the isoprene cluster, respectively, at the translated amino acid level. It was clear that neither *garB_1* or *garB_2* was involved in isoprene metabolism by the wild‐type strain, since neither transcripts nor translated gene products were detected by either ‘omics experiment (Supplementary Data sheets S1 and S2), but it was suspected that *garB1‐2* may provide a functional redundancy in WS11 Δ*garB*. The relative expression of the three *garB* genes was analysed by RT‐qPCR, relative to *rpoB*, in succinate‐grown and isoprene‐grown *Variovorax* sp. WS11 and the WS11 Δ*garB* mutant. *garB3* was upregulated by 97‐fold during wild‐type growth on isoprene, compared to the succinate‐grown condition (Figure S9), and neither chromosomal copy of *garB* was upregulated. The expression of *garB2* was significantly elevated in WS11 Δ*garB* compared to the wild‐type strain (*p* ≤ 0.05), but there was no change between the succinate‐grown and isoprene‐grown conditions in the mutant strain. The continued growth of *Variovorax* sp. WS11 Δ*garB* on isoprene may have been supported by de novo glutathione biosynthesis or possibly due to the activity of an alternative thiol recycling mechanism. *Escherichia coli* was resilient to the loss of glutathione‐disulfide reductase, presumed to be due to redundancy provided by the thioredoxin system, although a glutathione‐disulfide reductase‐deficient and thioredoxin‐deficient double‐mutant was similarly able to maintain intracellular reduced glutathione pools (Prinz et al., 1997; Tuggle & Fuchs, 1985). Thioredoxin 1 was highly abundant in the proteome of *Variovorax* sp. WS11 during growth on isoprene (Table S1), although its expression was not significantly different when compared to Timepoint 0 (0 h). The high level of expression of GarB_3 by *Variovorax* sp. WS11 specifically during growth on isoprene, coupled with the apparent conservation of *garB* in the *iso* gene clusters from Gram negative isoprene degraders (Carrión et al., 2020; Dawson et al., 2020), suggests an important but non‐essential role for this gene.

### ‘Omics‐guided prediction of the whole isoprene metabolic pathway in *Variovorax* sp. WS11

#### Predicted roles of uncharacterized *iso* genes

**FIGURE 4:**
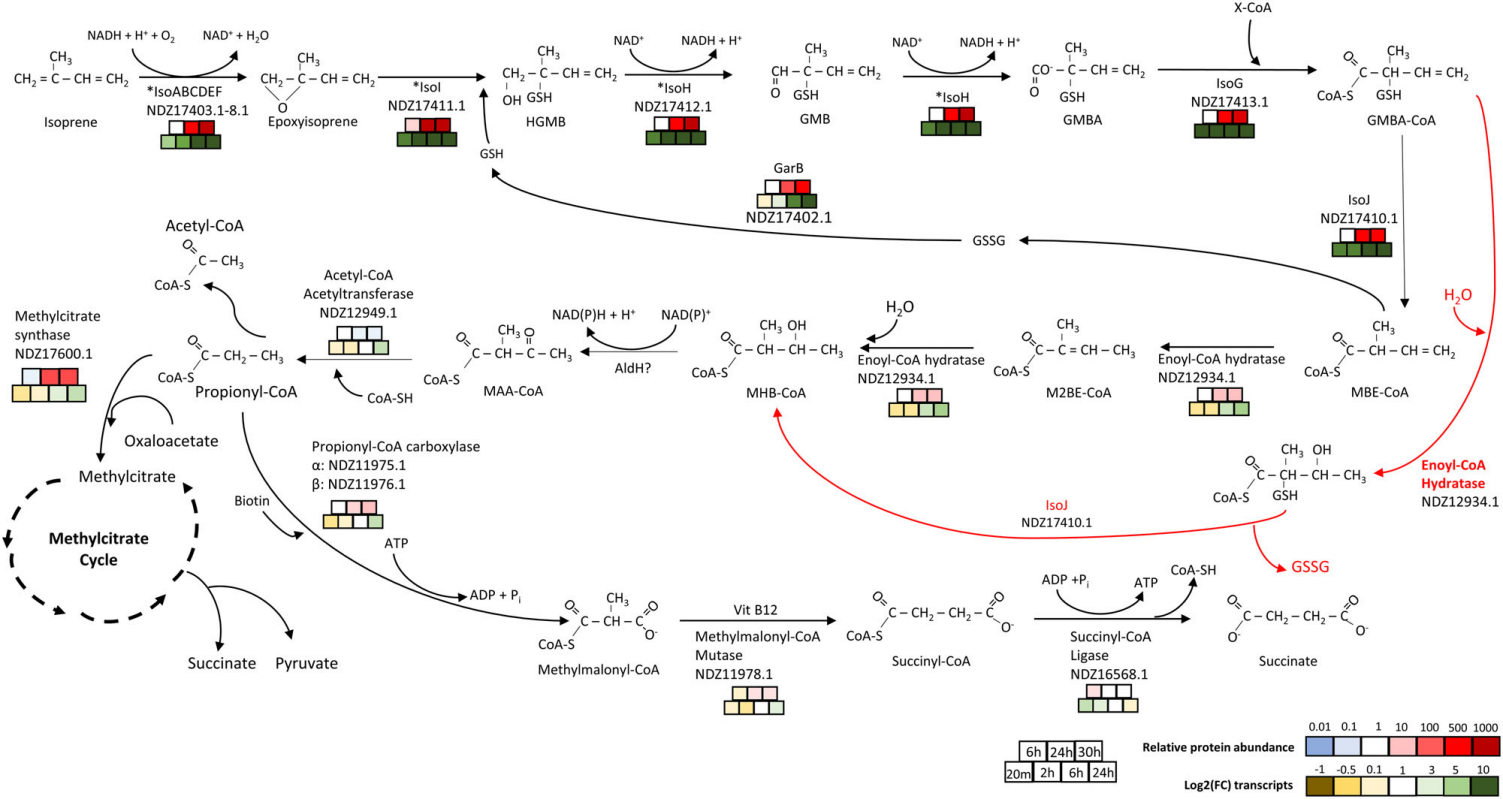
Predicted metabolic pathway to incorporate isoprene into central metabolism, as determined by transcriptomic and proteomic approaches. Confirmed enzymatic steps are denoted by an asterisk (*). Viable alternative metabolic reactions are denoted by red arrows. Relative peptide abundance was calculated (from left to right) after 6, 24, and 30 h of growth on isoprene, with downregulation (blue) or upregulation (red) denoted according to colour. Fold‐change (FC) in gene expression was recorded after 20 min, 2, 6, and 24 h of growth on isoprene (left to right), with downregulation (yellow) or upregulation (green) denoted by colour. CoA‐SH, free CoA; TCA, tricarboxylic acid; Vit B12, vitamin B12; X‐CoA: unidentified CoA‐thioester

Analysis of the proteome and transcriptome of *Variovorax* sp. WS11 strongly indicated the importance of genes involved in propionate assimilation during growth on isoprene, including propionyl‐CoA carboxylase, propionate‐CoA ligase, and methylcitrate synthase (Figure 4, Tables S1 and S2). The expression of proteins involved in propionate metabolism supports the notion that isoprene‐derived carbon is assimilated into central metabolism via β‐oxidation. This would require the conversion of GMBA to a CoA‐thioester, an essential component of the β‐oxidative metabolic process (Jimenez‐Diaz et al., 2017). *isoG* is a likely candidate due to its predicted role as a CoA‐transferase, as determined by BLASTp analysis (see Supplementary Materials and Methods S1), although the CoA‐donor is currently unknown (Figure 4). 2‐Methyl‐3‐butenyl‐CoA (MBE‐CoA) is then predicted to enter central metabolism via a series of β‐oxidative reactions (Figure 4). At present, no role has been assigned to AldH in the predicted isoprene metabolic pathway, although AldH is clearly essential for the growth of *Variovorax* sp. WS11 on isoprene (Figure S6). Further research is required to determine the function of this putative aldehyde dehydrogenase.

#### Assimilation of carbon from isoprene by β‐oxidation

The structure of GMBA suggests that it may be degraded similarly to branched‐chain amino acids, for example, isoleucine (Kohlmeier, 2003), requiring the formation of a CoA‐thioester and subsequent incorporation via β‐oxidation (Jimenez‐Diaz et al., 2017). NDZ12934.1, an enoyl‐CoA hydratase/isomerase family protein, was significantly expressed in the proteome of isoprene‐grown *Variovorax* sp. WS11 (*p* ≤ 0.01), but not in succinate‐grown cells, suggesting the role of this protein in isoprene metabolism. Enoyl‐CoA hydratase (ECH) catalyses the hydration of an alkene group in a CoA‐thioester to form a sub‐terminal hydroxyl group, but this typically occurs at the 2–3 carbon position rather than the 3–4 carbon position as in GMBA‐CoA. However, some enoyl‐CoA hydratase/isomerase family proteins may catalyse both the hydration and isomerase reactions (Palosaari & Hiltunen, 1990). MAFFT (Katoh et al., 2019) was used to align the deduced amino acid sequence of NDZ12934.1 with published ECH and enoyl‐CoA isomerase (ECI) sequences. Of the four conserved catalytic residues previously identified in ECH from *Rattus norvegicus* (Ala 98, Gly141, Glu144, Glu164) (Engel et al., 1996), NDZ12934.1 contained three of these residues (Ala98, Gly141, Glu164) and a substitution of glycine at position 144 (Figure S11). This substitution was also observed in ECH from *Thermus thermophilus* (1UIY_1) (Padavattan et al., 2021). We predict that NDZ12934.1 plays a bi‐functional role during isoprene metabolism as an enoyl‐CoA isomerase, conferred by the catalytic Glu164 (Onwukwe et al., 2015), and as an enoyl‐CoA hydratase, initially converting MBE‐CoA to 2‐methyl‐2‐butenyl‐CoA (M2BE‐CoA) and then introducing a hydroxyl group at carbon‐3 to form 2‐methyl‐3‐hydroxy‐burytyl‐CoA (MHB‐CoA) (Figure 4). Alternative, it is feasible that the double‐bond opening step could occur before the removal of glutathione (Figure 4).

Following the typical scheme of a β‐oxidation system, the hydroxyl group of MHB‐CoA would be oxidized to a keto group by an NAD^+^‐dependent 3‐hydroxyacyl‐CoA dehydrogenase, yielding 2‐methylacetoacetyl‐CoA (MAA‐CoA) (Menendez‐Bravo et al., 2017). These metabolic intermediates are also shared by the isoleucine metabolic pathway (Conrad et al., 1974), supporting their inclusion in the isoprene metabolic pathway on the basis of the similarities between formation and β‐oxidative breakdown of odd‐chain CoA‐thioesters. A putative 3‐hydroxyacyl‐CoA dehydrogenase (NDZ14590.1) was identified in the genome of *Variovorax* sp. WS11, although the deduced amino acid sequence shared only 26.0% identity with the functionally characterized 3‐hydroxyadipyl‐CoA dehydrogenase from *E. coli* K12 (P76083), indicating that the enzymatic functions may differ. Additionally, the expression of NDZ14590.1 was downregulated during growth on isoprene (Supplementary Data sheet S3, L31), and the translated gene product was present at only 1.93% of the abundance of IsoA after 30 h of growth on isoprene (Supplementary Data sheet S2, L2539). Therefore, NDZ14590.1 is unlikely to be involved in isoprene metabolism. At present, no satisfactory gene candidate has been identified, which catalyses the oxidation of HMB‐CoA to MAA‐CoA. Further analysis through targeted mutagenesis and metabolomics are required in order to confirm the predicted isoprene metabolic pathway (Figure 4).

**FIGURE 5:**
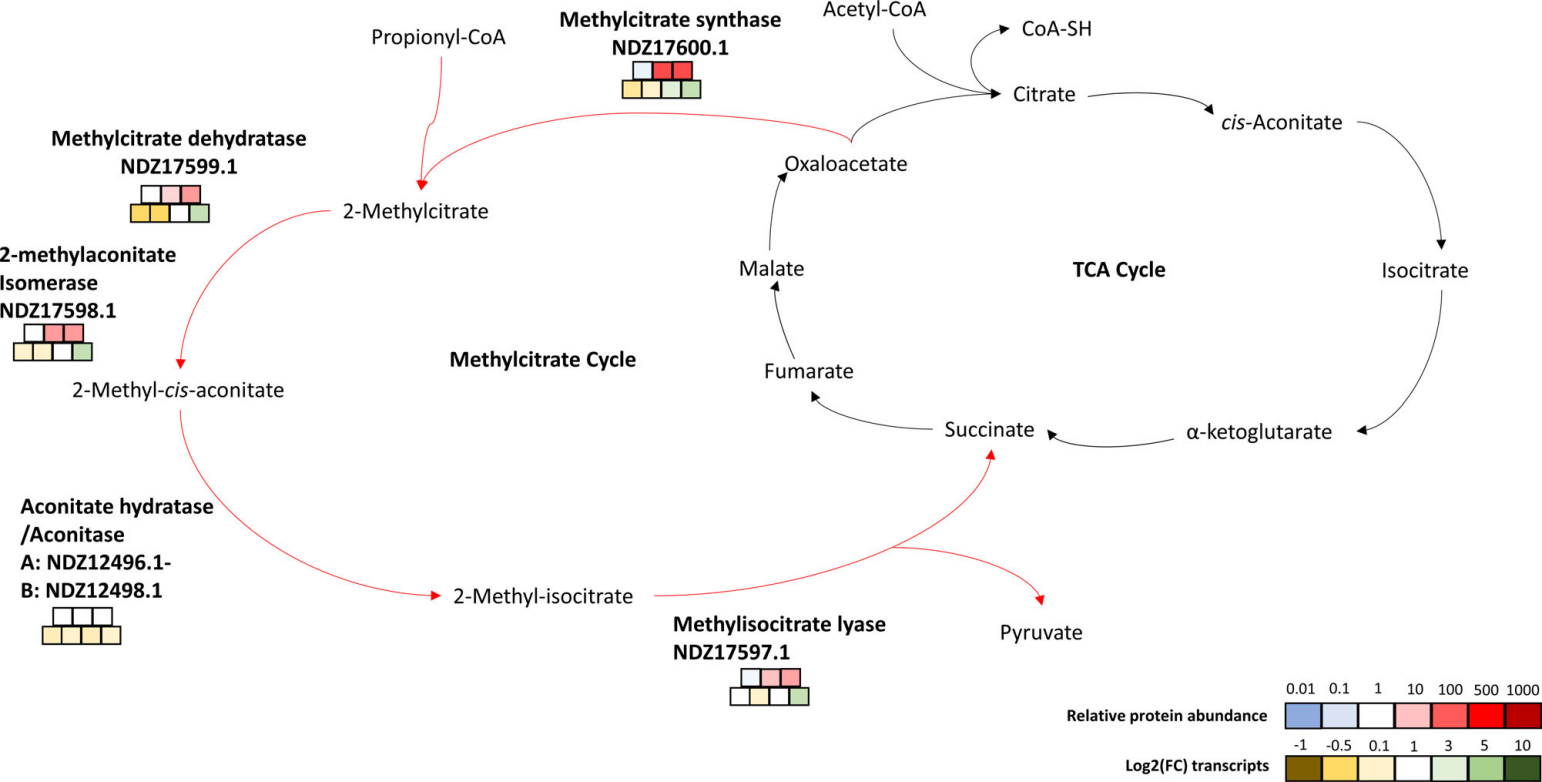
Incorporation of propionyl‐CoA into the TCA cycle (black arrows) via the methylcitrate cycle (red arrows), with candidate genes identified by proteomic and transcriptomic analysis during growth of *Variovorax* sp. WS11 on isoprene. Relative peptide abundance was calculated (from left to right) after 6, 24, and 30 h of growth on isoprene, with downregulation (blue) or upregulation (red) denoted according to colour. Fold‐change (FC) in gene expression was recorded after 20 min, 2, 6, and 24 h of growth on isoprene (left to right), with downregulation (yellow) or upregulation (green) denoted by colour.

Following the formation of MAA‐CoA, a typical β‐oxidative metabolic pathway would be followed via thiolysis, the process by which a second CoA group is added at the sub‐terminal carboxyl group, causing a split of the C5 MAA‐CoA into the C2 acetyl‐CoA and C3 propionyl‐CoA. Thiolysis is catalysed by acetyl‐CoA acetyltransferase (ketoacyl‐CoA thiolase) (Kunau et al., 1995). NDZ12949.1, a predicted acetyl‐CoA acetyltransferase, was present at moderately high abundance under all growth conditions in the proteome experiment (Supplementary Data sheet S2, L1251 and Data sheet 3, L58), amounting to 91.5% of the abundance of IsoA after 24 h of growth on isoprene, which itself was present in the top 1% of proteins in terms of abundance at this timepoint (Timepoint 4). This indicated that NDZ12949.1 was constitutively expressed at a relatively high level, and therefore was the most likely candidate to catalyse the thiolysis reaction in the predicted isoprene metabolic pathway (Figure 4). The metabolic pathways for propionyl‐CoA assimilation have been well studied, with propionyl‐CoA able to enter central metabolism via methylmalonyl‐CoA to succinyl‐CoA, catalysed by propionyl‐CoA carboxylase (α‐subunit: NDZ11975.1, β‐subunit: NDZ11976.1) and methylmalonyl‐CoA mutase (NDZ11978.1), or through conversion to methylcitrate using the methylcitrate cycle (NDZ17597‐600.1) (Textor et al., 1997). Genes encoding all the enzymes of both these pathways (Figures 4 and 5) are present in the *Variovorax* sp. WS11 genome. Additional copies of propionyl‐CoA carboxylase α‐ and β‐subunits were also located on an isoprene‐induced gene cluster (Figure S2B). The ‘omics experiments confirmed the significantly increased expression of genes which encode putative propionyl‐CoA carboxylase, methylmalonyl‐CoA mutase and methylcitrate synthase enzymes (Figures 4 and 5 and Figure S12), strongly indicating the presence of propionyl‐CoA as an intermediate metabolite in isoprene metabolism, thereby supporting the proposed β‐oxidative pathway. A dependence on vitamins may have been introduced into the isoprene metabolic pathway by propionyl‐CoA carboxylase, which requires biotin (Tong, 2013; Wongkittichote et al., 2017), and methylmalonyl‐CoA mutase, which requires vitamin B12 (Takahashi‐Iñiguez et al., 2012). The roles of these vitamins in isoprene metabolism were investigated further.

#### Conversion of isoprene‐derived propionyl‐CoA into central metabolic intermediates

Biotin biosynthesis genes were located on an isoprene‐induced gene cluster (Figure S2B), but the genome of *Variovorax* sp. WS11 only contained 5 (*cobDGNSU*) of the approximately 30 genes (Roth et al., 1993) required for the synthesis of vitamin B12. The removal of biotin, vitamin B12, or both vitamins from Ewers medium had varying effects on the growth rate of *Variovorax* sp. WS11 on isoprene (Figure S13), with the greatest decrease in growth observed in the absence of both vitamins. Propionate was also tested as propionate metabolism proceeds via propionyl‐CoA, initiated by propionyl‐CoA synthase (Horswill & Escalante‐Semerena, 2002). While little difference was seen between the final OD_540_ of the cultures according to vitamin availability, the absence of both biotin and vitamin B12 once again lowered the growth rate of *Variovorax* sp. WS11 (Figure S14). Overall, these data suggest that the absence of both biotin and vitamin B12 reduces the growth rate of *Variovorax* sp. WS11 on propionate, potentially due to the predicted need to assimilate carbon from each substrate via propionyl‐CoA.

The significant upregulation of the megaplasmid 1‐encoded methylcitrate pathway gene cluster during growth on isoprene suggested that *Variovorax* sp. WS11 was also capable of assimilating propionyl‐CoA via this pathway (Figures 2C and 5). Methylcitrate synthase, catalysing the first step of the methylcitrate pathway, was over 100‐fold more abundant in isoprene‐grown cells compared to succinate‐grown cells after 24 h (Figure 5, Supplementary Data sheet S2, L4589 and Data sheet S3, L21). All of the methylcitrate cycle enzymes were expressed to a significantly greater degree (relative abundances ranged from 41.6 to 111.2) during growth on isoprene, with the exception of aconitase (Figure 5). These ‘omics data strongly indicate the ability of *Variovorax* sp. WS11 to metabolize isoprene via differing metabolic pathways subsequent to the formation of propionyl‐CoA (Figures 4 and 5), perhaps with the chosen pathway varying depending on the vitamins in the growth medium. Surprisingly, the methylcitrate cycle and methylmalonyl‐CoA pathway‐encoding genes were not upregulated in the transcriptome of isoprene‐grown *Rhodococcus* sp. AD45 (Crombie et al., 2015), suggesting that the Gram positive model isoprene degrader may assimilate carbon from isoprene by a different pathway, or that constitutive expression of these enzymes is sufficient to accommodate additional carbon from isoprene. However, transcriptome data cannot be directly correlated with protein expression, indicating the need for proteomics and metabolomics studies.

Both the *iso* gene cluster and methylcitrate gene cluster are found on Megaplasmid 1, although it is unknown whether these gene clusters were horizontally acquired. Not all *Variovorax* spp. have acquired the methylcitrate gene cluster, such as *Variovorax* sp. RA8 (GCA_901827175.1), while the genome of *Variovorax paradoxus* B4 (GCA_000463015.1) contains genes encoding 2‐methylcitrate synthase (*prpC*) and 2‐methylcitrate dehydratase (*prpD*), with the *prpD* gene annotated as a bi‐functional 2‐methylcitrate dehydratase/aconitate hydratase which shared no identity with methylcitrate dehydratase (*acnD*) from *Variovorax* sp. WS11 when compared by BLASTp. Instead, *prpD* from *V. paradoxus* B4 shared 84% predicted amino acid identity with a non‐isoprene‐induced chromosomal copy of *prpD* (NDZ16656.1, also a bi‐functional 2‐methylcitrate dehydratase/aconitate hydratase) in *Variovorax* sp. WS11, which was adjacent to a second copy of methylcitrate synthase (*prpC_2* ‐ NDZ16657.1) which had a relative expression of 91.4 after 30 hours of growth on isoprene (Supplementary Data sheet S2, L5029). PrpC_2 was provisionally identified using only two unique peptides and shared 85.9% predicted amino acid identity with the megaplasmid‐encoded PrpC_1, indicating that PrpC_2 may have been wrongly identified in the proteome. Transcription of the chromosomal *prpC_2* and *prpD* genes was not upregulated in the transcriptome of isoprene‐grown cells (Supplementary Data sheet S3, L24‐25), further indicating that only the plasmid‐encoded methylcitrate genes were upregulated during growth on isoprene.

An isoprene‐induced gene cluster (NDZ12922.1–NDZ12938.1) measuring approximately 20 kbp was located on the chromosome of *Variovorax* sp. WS11 (S1b) with predicted roles in fatty acid metabolism and biotin synthesis, including acetyl‐/propionyl‐CoA carboxylase α‐subunit (NDZ12928.1) and β‐subunit (NDZ12936.1), acyl‐CoA dehydrogenase (NDZ12938.1), and propionate‐CoA ligase (NDZ12937.1) (Figure S2B, Supplementary Data sheet S3, L33‐48). The enoyl‐CoA hydratase/isomerase (NDZ12934.1), which was assigned a role in the predicted isoprene metabolic pathway (Figure 4) is also located in this gene cluster. Also encoded on the chromosome is a predicted 2‐oxoisovaleryl‐CoA dehydrogenase complex (NDZ14665‐667.1), which was significantly expressed in the proteome during growth on isoprene (Supplementary Data sheet S3, L50‐52), with growth on succinate inducing expression of these proteins to a lesser degree, indicating that *Variovorax* sp. WS11 broke down branched‐chain amino acids during growth on isoprene in order to replenish intracellular stores of reductant. However, the transcription of genes encoding this protein complex was not similarly upregulated (Supplementary Data sheet S3). Without conducting further analysis through targeted mutagenesis and metabolomics, we cannot rule out the involvement of the cluster shown in Figure S1B in isoprene metabolism.

#### Comparative analysis of the genomes of extant isoprene degraders

The genomes of cultivated isoprene‐degrading bacteria were studied for the presence of genes involved in the predicted isoprene metabolic pathway (Table S3). Propionyl‐CoA carboxylase and methylmalonyl‐CoA mutase were present in all tested genomes, but only *Variovorax* sp. OPL2.2 (Carrión et al., 2020) contained the entire methylcitrate cycle gene cluster found in *Variovorax* sp. WS11. This was unsurprising as *Variovorax* sp. WS11 and OPL2.2 share 99.9% average nucleotide identity (Carrión et al., 2020), suggesting that these isolates are closely related. The methylcitrate gene cluster from *Variovorax* sp. WS11 was analysed by BLAST against the nucleotide collection (nr/nt), with the closest relative at the nucleotide level found in *Acidovorax avenae* KL3 (GCA_003029905.1) (99% coverage, 80.65% identity), although this bacterium contains no isoprene gene cluster. *Ramlibacter* sp. WS9 (GCA_003797765.1), a Gram negative isoprene degrader, and *Rhodococcus* sp. AD45 (GCA_000949305.1), a Gram positive isoprene degrader, each contained the methylcitrate cycle‐encoding genes except for methylaconitate isomerase, indicating that they may lack the ability to convert mixed 2‐methyl‐*cis/trans*‐aconitate into 2‐methyl‐*cis*‐aconitate (Dolan et al., 2018). The Gram positive isoprene degraders *Nocardioides* sp. WS12 (GCA_014108865.1) and *Sphingopyxis* sp. OPL5 (GCA_003797775.2) do not encode complete methylcitrate pathways, suggesting a reliance on incorporating carbon from propionyl‐CoA via methylmalonyl‐CoA. The presence of an alternative metabolic pathway for the incorporation of carbon from isoprene cannot be discounted in these extant isoprene degraders. Overall, comparative genomics analysis suggested that the metabolic pathway predicted in *Variovorax* sp. WS11 is available to distinct isoprene degraders, albeit in varying levels of completeness, but the need for additional proteomics and metabolomics studies is clearly indicated.

## CONCLUSIONS

Using a combination of proteomics and transcriptomics, a complete isoprene metabolic pathway has been proposed for the first time. Gene expression during growth on isoprene strongly indicated a β‐oxidative pathway to generate propionyl‐CoA from GMBA, with confirmed roles for essential *iso* genes (*isoG*, *isoJ*, and *aldH*) likely to contribute to the assimilation of carbon from isoprene. While essential roles have been confirmed for previously uncharacterized *iso* genes, specific enzyme activity assays must now be developed for IsoG, IsoJ and AldH with a view to confirming the enzymatic steps predicted in this study. The incorporation of isoprene‐derived carbon is likely to vary according to the vitamins available to *Variovorax* sp. WS11 during growth, such as biotin and vitamin B12, permitting the conversion of propionyl‐CoA to methylmalonyl‐CoA or methylcitrate. The significant upregulation of the megaplasmid‐encoded methylcitrate gene cluster strongly indicated that *Variovorax* sp. WS11 had acquired an additional mechanism of incorporating carbon from isoprene, one that was not available to all extant isoprene degraders. Metabolomics analysis of isoprene‐grown *Variovorax* sp. WS11 is now required to verify the metabolic predictions made in this study. Additional proteomics studies are also required to assess the use of the proposed pathway by a greater diversity of isoprene degrading bacteria.

## FUNDING INFORMATION

The work on this project was funded through a European Research Council (ERC) Advanced Grant to J. C. Murrell (694578‐IsoMet), a Natural Environment Research Council (NERC) grant to J. C. Murrell (NE/J009725/1), a Leverhulme Trust Early Career Fellowship (ECF‐2016‐626) to A. T. Crombie and the Earth and Life Systems Alliance (ELSA) at the University of East Anglia.

## CONFLICT OF INTEREST

The authors declare no conflict of interest that could influence this work.

## Supporting information


**Figure S1** Confirmed steps of isoprene metabolism, adapted from van Hylckama Vlieg et al. (2000). Steps which are catalysed by enzymes are marked by an asterisk. IsoABCDEF: isoprene monooxygenase. IsoI: glutathione *S*‐transferase. IsoH: dehydrogenase.

**Figure S2** (A) *iso* metabolic gene cluster encoded on Megaplasmid 1 of *Variovorax* sp. WS11 (taken from Dawson et al., 2020). (B) Putative isoprene‐induced gene cluster with predicted roles in β‐oxidation. *acdA*: acyl‐CoA dehydrogenase (NDZ12938.1), *fadD*: propionyl‐CoA ligase (NDZ12937.1), *pccB*: propionyl‐CoA carboxylase, β‐chain (NDZ12936.1), *hypoth*: hypothetical protein (NDZ12935.1, NDZ12933.1), *dpgD*: enoyl‐CoA hydratase (NDZ12934.1), *bioA*: adenosylmethionine‐8‐amino‐7‐oxononanoate aminotransferase (NDZ12932.1), *bioF*: 8‐amino‐7‐oxononanoate synthase (NDZ12931.1), *bioD*: ATP‐dependent dethiobiotin synthetase (NDZ12930.1), *bioB*: biotin synthase (NDZ12929.1), *accA*: acetyl‐/propionyl‐CoA carboxylase α‐chain (NDZ12928.1). (C) Putative methylcitrate pathway gene cluster. *prpC*: 2‐methylcitrate synthase (NDZ17600.1), *acnD*: 2‐methylisocitrate dehydratase (NDZ17599.1), *prpF*: 2‐methylaconitate isomerase (NDZ17598.1), *dml*: 2,3‐dimethylmalate lyase (NDZ17597.1).

**Figure S3** *iso* metabolic gene transcripts (normalized read counts vs. fragments per kilobase million) as a percentage of all detected transcripts during growth on isoprene or incubation with epoxyisoprene, measured over time.

**Figure S4** Growth of *Variovorax* sp. WS11 Δ*isoG* on 1% (v/v) isoprene, compared with growth of *Variovorax* sp. WS11 Δ*isoG* transformed with pBBR1:*isoG*. Error bars represent the standard deviation about the mean (*n* = 3). An asterisk (*) denotes a statistically significant difference between the indicated data (*p* ≤ 0.05), determined by student's *t*‐test.

**Figure S5** Growth of *Variovorax* sp. WS11 Δ*isoJ* on 1% (v/v) isoprene, compared with the growth of *Variovorax* sp. WS11 Δ*isoJ* transformed with pBBR1:*isoJ*. Error bars represent the standard deviation about the mean (*n* = 3). An asterisk denotes a statistically significant difference between the indicated conditions (*p ≤ 0.05; **p ≤ 0.01).

**Figure S6** Growth of *Variovorax* sp. WS11 Δ*aldH* on 1% (v/v) isoprene, compared with the growth of *Variovorax* sp. WS11 Δ*aldH* transformed with pBBR1:*aldH*. Error bars represent the standard deviation about the mean (*n* = 3). An asterisk denotes a statistically significant difference (*p ≤ 0.05; **p ≤ 0.01).

**Figure S7** Isoprene oxidation by *Variovorax* sp. WS11 grown in the presence of 1% (v/v) isoprene, 10 mM pyruvate, or a combination of 1% (v/v) isoprene and 10 mM pyruvate, measured as nmol isoprene consumed minute^−1^ mg dry weight^−1^. Error bars represent the standard deviation about the mean (*n* = 3). An asterisk denotes a statistically significant difference (*p* ≤ 0.05) between the indicated conditions, determined by students *t*‐test.

**Figure S8** Growth of *Variovorax* sp. WS11 Δ*garB* on 1% (v/v) isoprene, compared to wild‐type *Variovorax* sp. WS11. Error bars represent the standard deviation about the mean (*n* = 3). An asterisk denotes a statistically significant difference between the indicated conditions (*p* ≤ 0.01).

**Figure S9** Fold‐change in expression of *garB_1*, *garB_2*, and *garB_3* by *Variovorax* sp. WS11 (wild‐type) and *Variovorax* sp. WS11 Δ*garB*, during growth on isoprene compared to growth on succinate, determined by RT‐qPCR, relative to the expression of *rpoB*. The fold‐change in expression of *garB_3* determined by RNA‐seq was calculated relative to timepoint 0. Error bars represent the standard deviation about the mean (*n* = 3). An asterisk (*) denotes a statistically significant difference between the indicated conditions (*p* ≤ 0.05).

**Figure S10** Isoprene‐induced changes in the transcription of typical β‐oxidation genes (bars), and the relative protein abundance of the respective gene products (lines).

**Figure S11** Alignment of the deduced amino acid sequence of NDZ12934.1 (ECH) with published ECH and ECI sequences, using the MAFFT online multiple sequence alignment tool with default parameters (Katoh et al., 2019). Conserved residues [Ala98, Gly142, Glu144, Glu164, based on alignment against the *Rattus norvegicus* ECH (Padavattan et al., 2021)] are highlighted in red. Glu144 has been substituted with a glycine residue in *Variovorax* sp. WS11. Where available, protein databank identifiers have been included in brackets.

**Figure S12** Isoprene‐induced changes in the expression of transcribed genes (bars) and translated gene products (lines) with predicted roles in the isoprene metabolic pathway (Figure 4), subsequent to GMBA formation by IsoH.

**Figure S13** Growth of *Variovorax* sp. WS11 on 1% (v/v) isoprene in the presence of a complete vitamin solution, in the absence of biotin (−biotin), in the absence of vitamin B12 (‐B12), or in the absence of both biotin and vitamin B12 (‐Biotin ‐B12). Error bars represent the standard deviation about the mean (*n* = 3). An asterisk denotes a statistically significant difference between the indicated conditions (*p ≤ 0.05, **p ≤ 0.01).

**Figure S14** Growth of *Variovorax* sp. WS11 on 10 mM propionate in the presence of all vitamins, in the absence of biotin (−biotin), in the absence of vitamin B12 (‐B12), or in the absence of both biotin and vitamin B12 (‐Biotin ‐B12). Error bars represent the standard deviation about the mean (*n* = 3).

**Appendix S1** Supporting Information.

**Appendix S2** Supporting Information.

**Appendix S3** Supporting Information.

**Appendix S4** Supporting Information.

**Table S1** Top 30 most abundant transcripts and polypeptides after 24 hours' growth on isoprene, calculated as the fold‐change in transcripts and ratio of abundance of polypeptides, compared to the respective timepoint 0 samples.

**Table S2** Top 30 most upregulated transcripts and polypeptides after 24 hours' growth on isoprene, calculated as the fold‐change in transcripts and the ratio of abundance of polypeptides, compared to the respective timepoint 0 samples.

**Table S3** Presence (✓) or absence (X) of genes involved in the methylcitrate pathway and propionyl‐CoA assimilation pathways in isoprene degrading bacteria.

**Table S4** Vectors used in this study.

**Table S5** Primers used in this study.

## Data Availability

Transcriptomic and proteomic data are available in Supplementary Data sheets S1 and S2, respectively. Data, which were specifically referred to in the text, are shown in Supplementary Data sheet S3.
