# Supplementary figures and images for: ‘Omics‐guided prediction of the pathway for metabolism of isoprene by Variovorax sp. WS11
Source: Environ Microbiol. 2022 Aug 5;24(11):5151–64. doi: 10.1111/1462-2920.16149 (PMC9804861; doi:10.1111/1462-2920.16149)

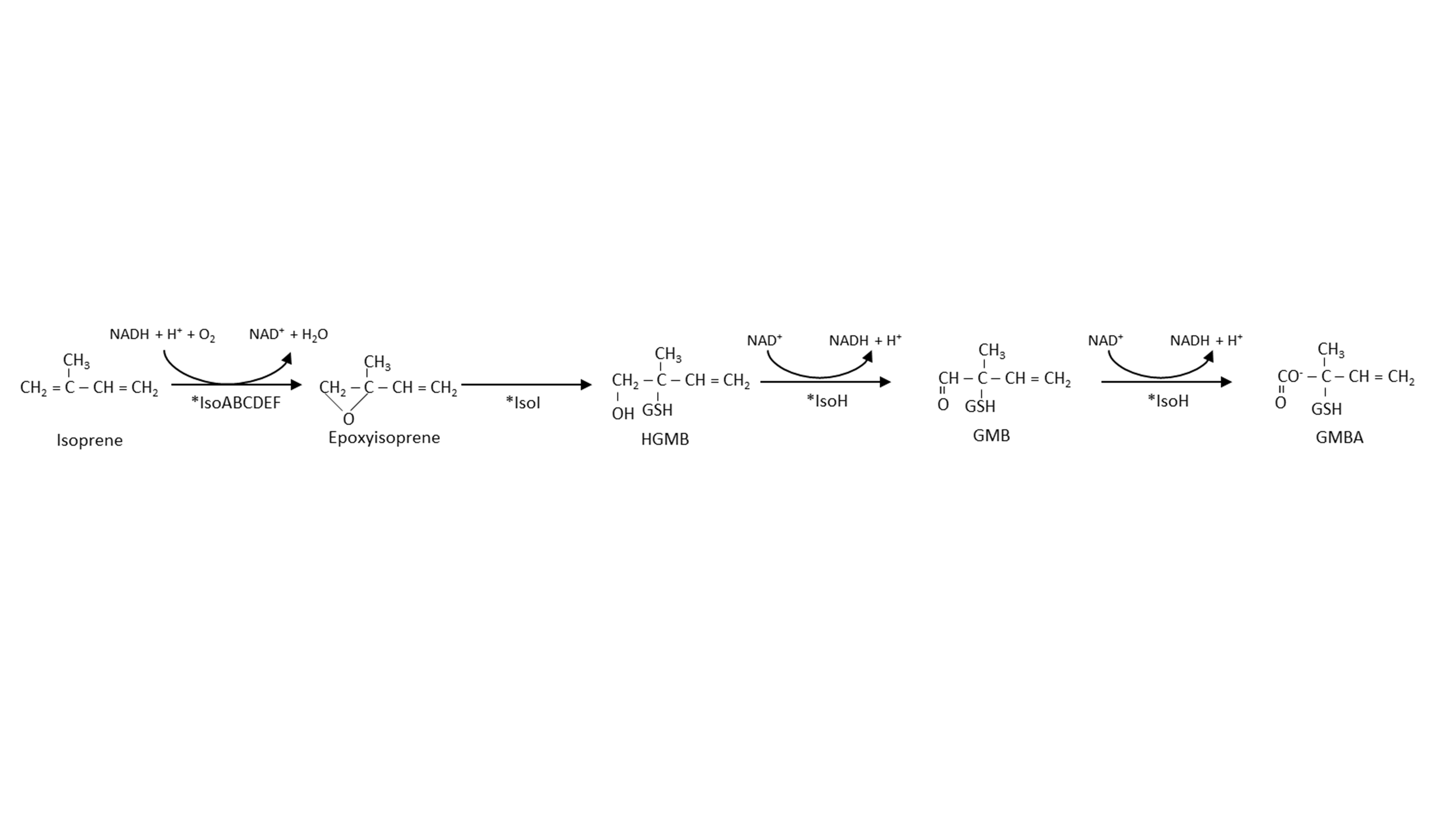

Supplement: Supplementary file 1 — Figure S1 Confirmed steps of isoprene metabolism, adapted from van Hylckama Vlieg et al. (2000). Steps which are catalysed by enzymes are marked by an asterisk. IsoABCDEF: isoprene monooxygenase. IsoI: glutathione S‐transferase. IsoH: dehydrogenase. [file EMI-24-5151-s009.tif]

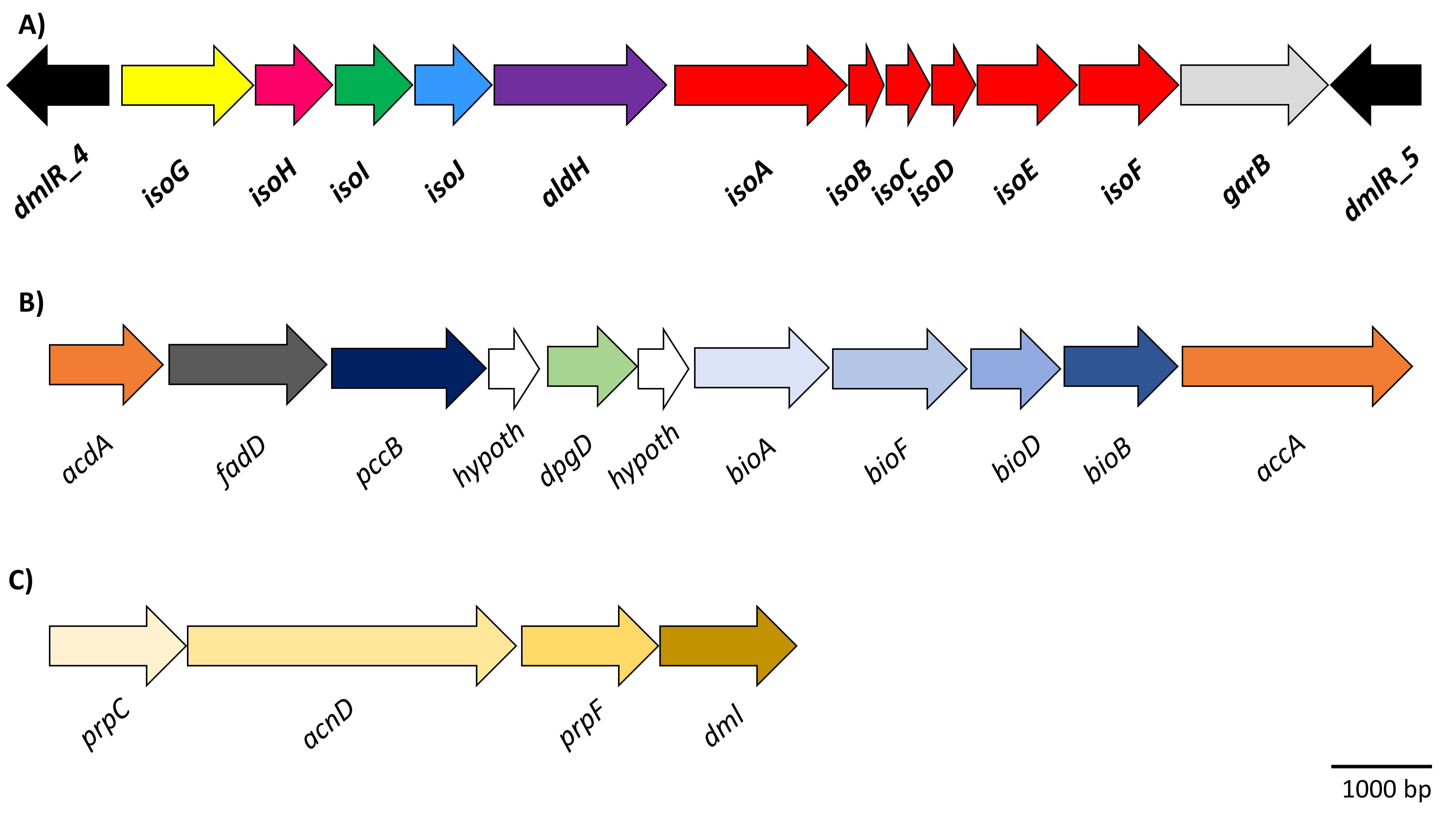

Supplement: Supplementary file 2 — Figure S2 (A) iso metabolic gene cluster encoded on Megaplasmid 1 of Variovorax sp. WS11 (taken from Dawson et al., 2020). (B) Putative isoprene‐induced gene cluster with predicted roles in β‐oxidation. acdA: acyl‐CoA dehydrogenase (NDZ12938.1), fadD: propionyl‐CoA ligase (NDZ12937.1), pccB: propionyl‐CoA carboxylase, β‐chain (NDZ12936.1), hypoth: hypothetical protein (NDZ12935.1, NDZ12933.1), dpgD: enoyl‐CoA hydratase (NDZ12934.1), bioA: adenosylmethionine‐8‐amino‐7‐oxononanoate aminotransferase (NDZ12932.1), bioF: 8‐amino‐7‐oxononanoate synthase (NDZ12931.1), bioD: ATP‐dependent dethiobiotin synthetase (NDZ12930.1), bioB: biotin synthase (NDZ12929.1), accA: acetyl‐/propionyl‐CoA carboxylase α‐chain (NDZ12928.1). (C) Putative methylcitrate pathway gene cluster. prpC: 2‐methylcitrate synthase (NDZ17600.1), acnD: 2‐methylisocitrate dehydratase (NDZ17599.1), prpF: 2‐methylaconitate isomerase (NDZ17598.1), dml: 2,3‐dimethylmalate lyase (NDZ17597.1). [file EMI-24-5151-s005.tif]

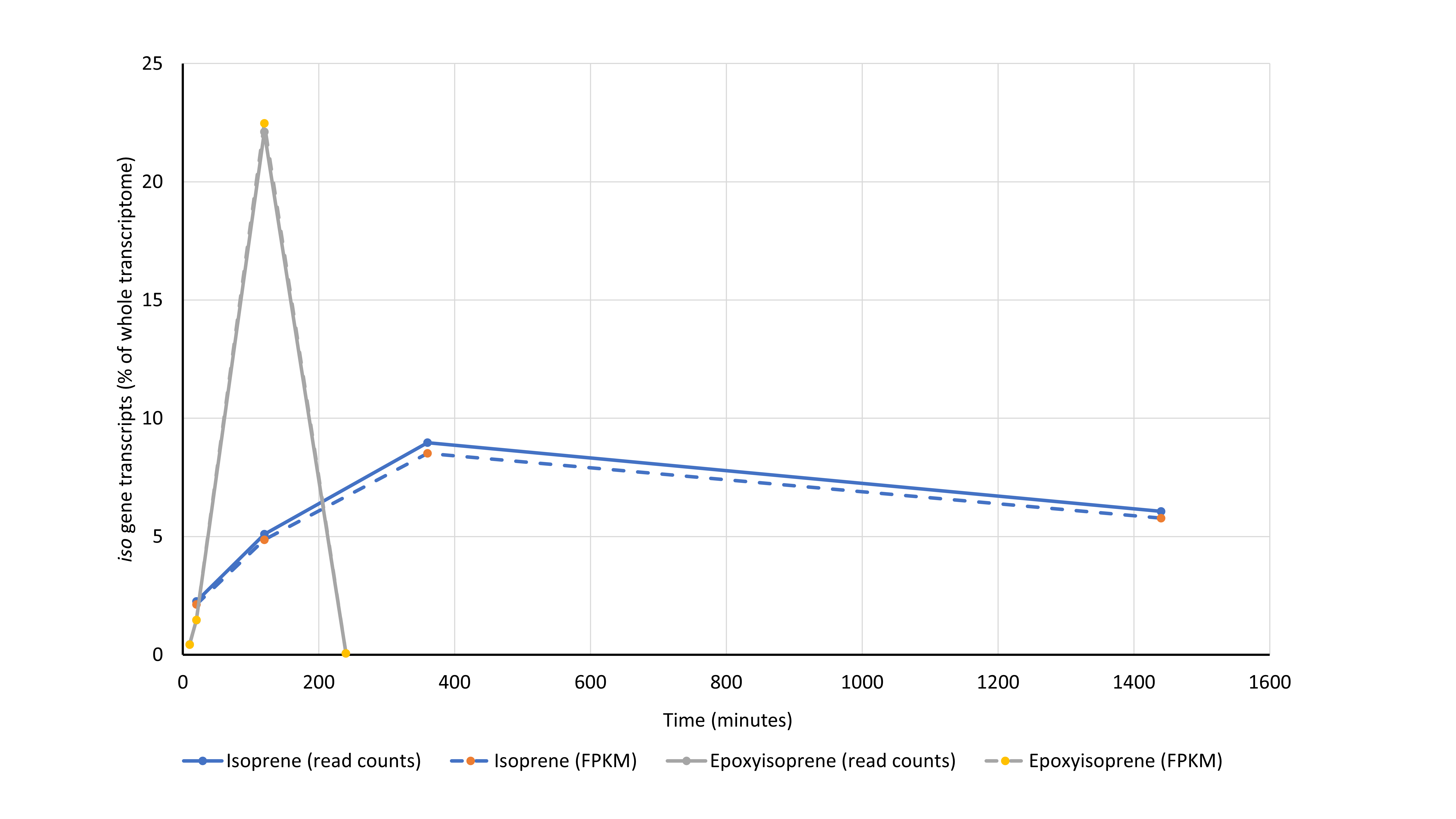

Supplement: Supplementary file 3 — Figure S3 iso metabolic gene transcripts (normalized read counts vs. fragments per kilobase million) as a percentage of all detected transcripts during growth on isoprene or incubation with epoxyisoprene, measured over time. [file EMI-24-5151-s015.tif]

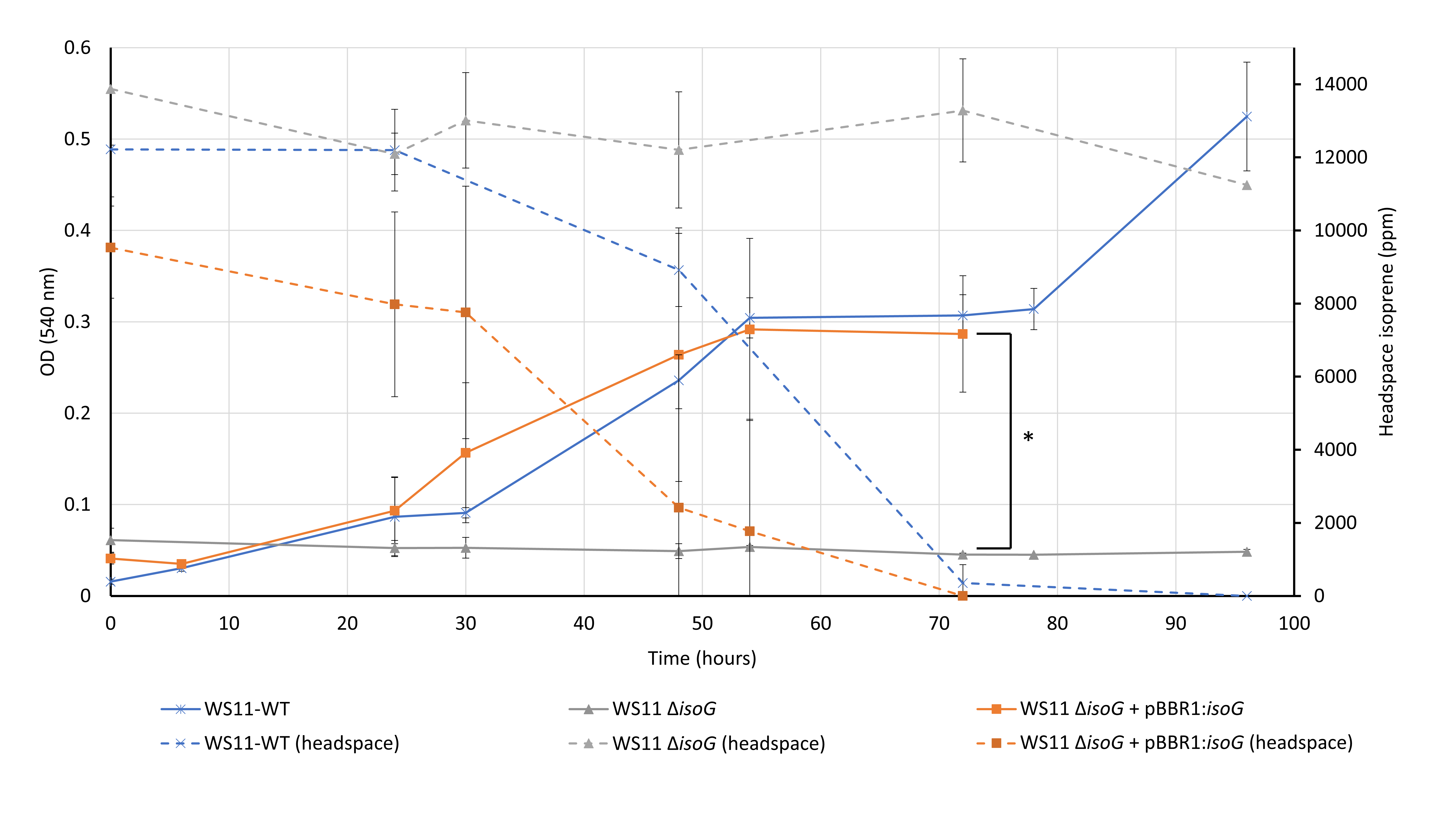

Supplement: Supplementary file 4 — Figure S4 Growth of Variovorax sp. WS11 ΔisoG on 1% (v/v) isoprene, compared with growth of Variovorax sp. WS11 ΔisoG transformed with pBBR1:isoG. Error bars represent the standard deviation about the mean (n = 3). An asterisk (*) denotes a statistically significant difference between the indicated data (p ≤ 0.05), determined by student's t‐test. [file EMI-24-5151-s013.tif]

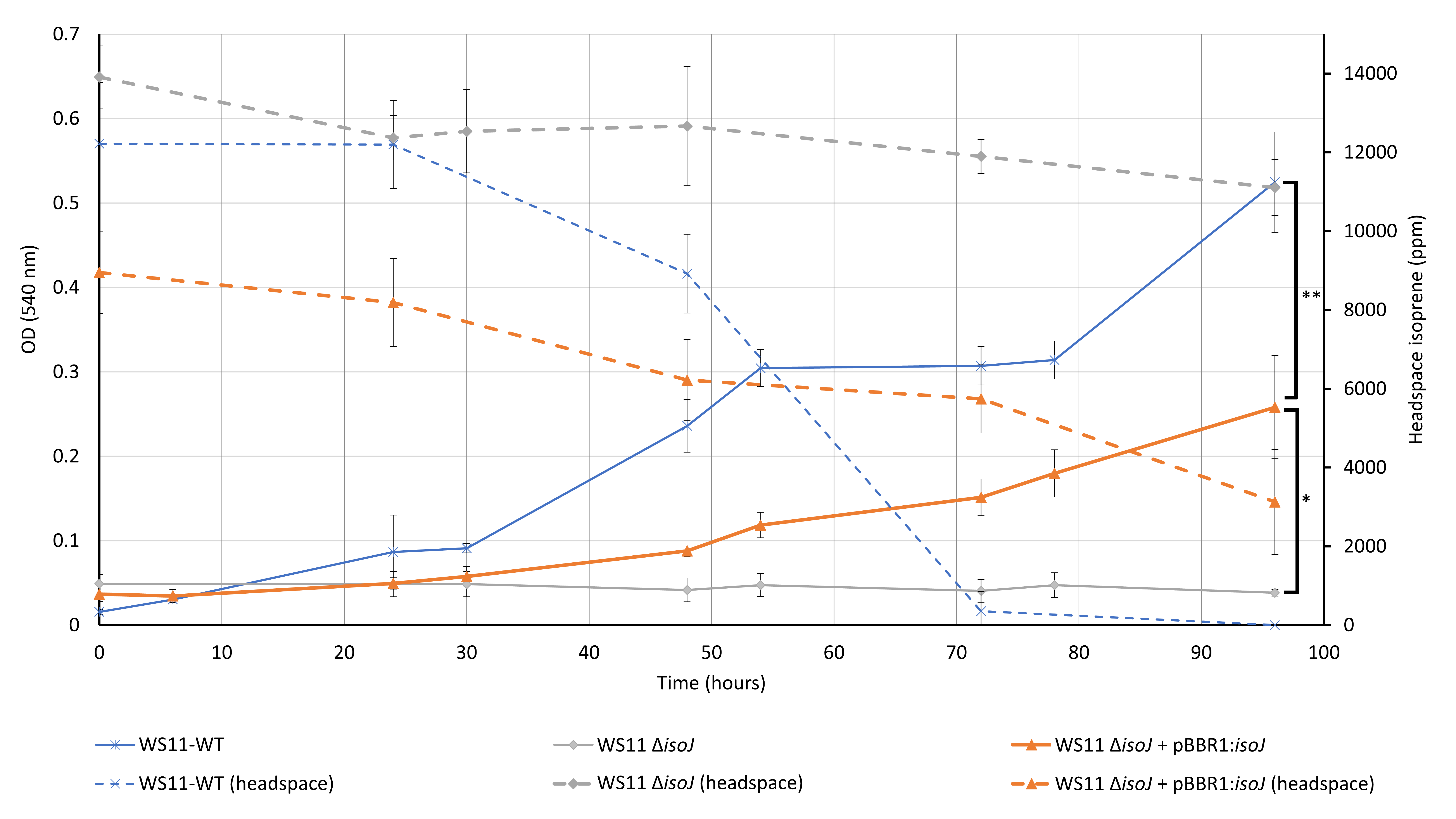

Supplement: Supplementary file 5 — Figure S5 Growth of Variovorax sp. WS11 ΔisoJ on 1% (v/v) isoprene, compared with the growth of Variovorax sp. WS11 ΔisoJ transformed with pBBR1:isoJ. Error bars represent the standard deviation about the mean (n = 3). An asterisk denotes a statistically significant difference between the indicated conditions (*p ≤ 0.05; **p ≤ 0.01). [file EMI-24-5151-s001.tif]

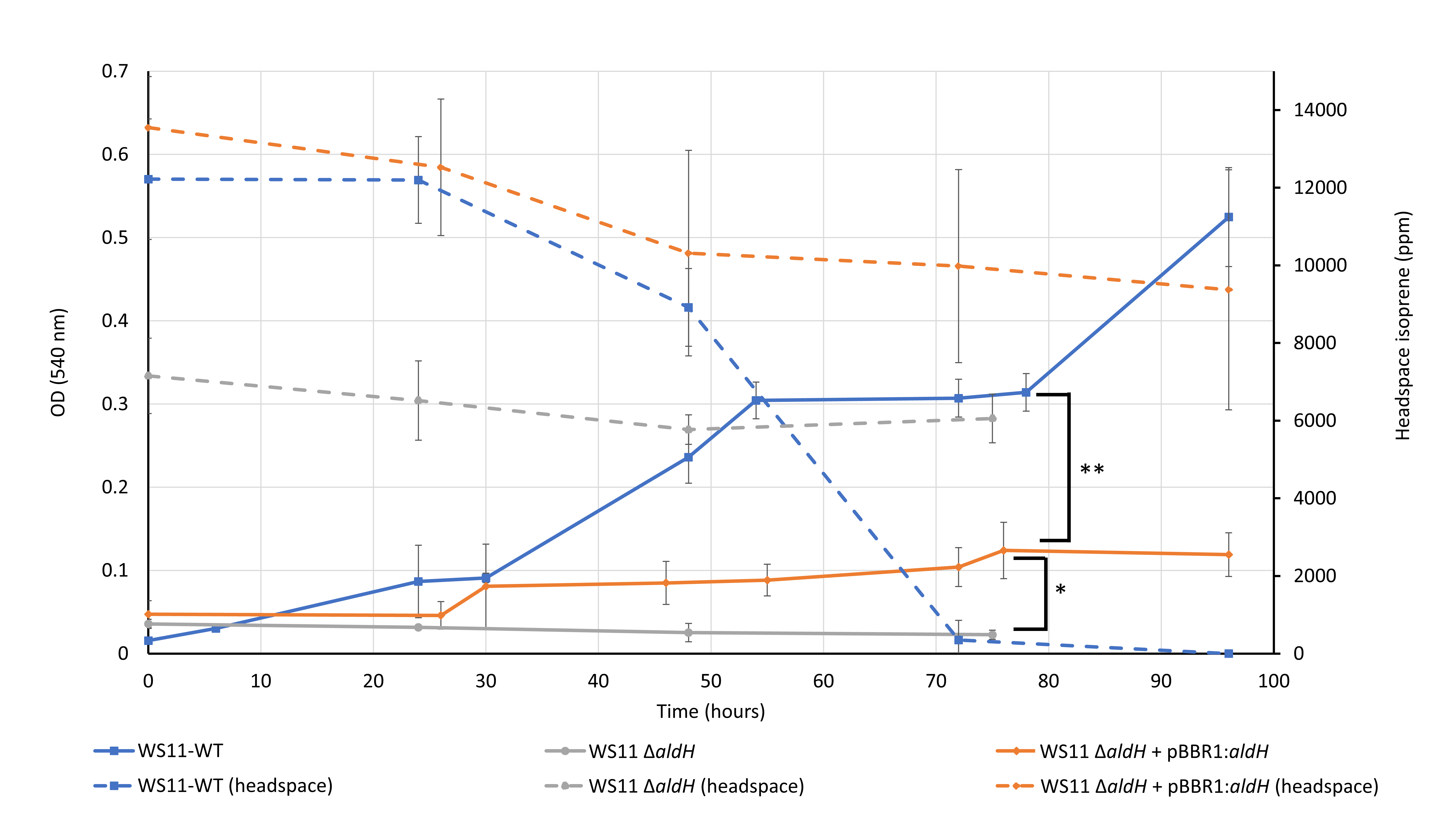

Supplement: Supplementary file 6 — Figure S6 Growth of Variovorax sp. WS11 ΔaldH on 1% (v/v) isoprene, compared with the growth of Variovorax sp. WS11 ΔaldH transformed with pBBR1:aldH. Error bars represent the standard deviation about the mean (n = 3). An asterisk denotes a statistically significant difference (*p ≤ 0.05; **p ≤ 0.01). [file EMI-24-5151-s007.tif]

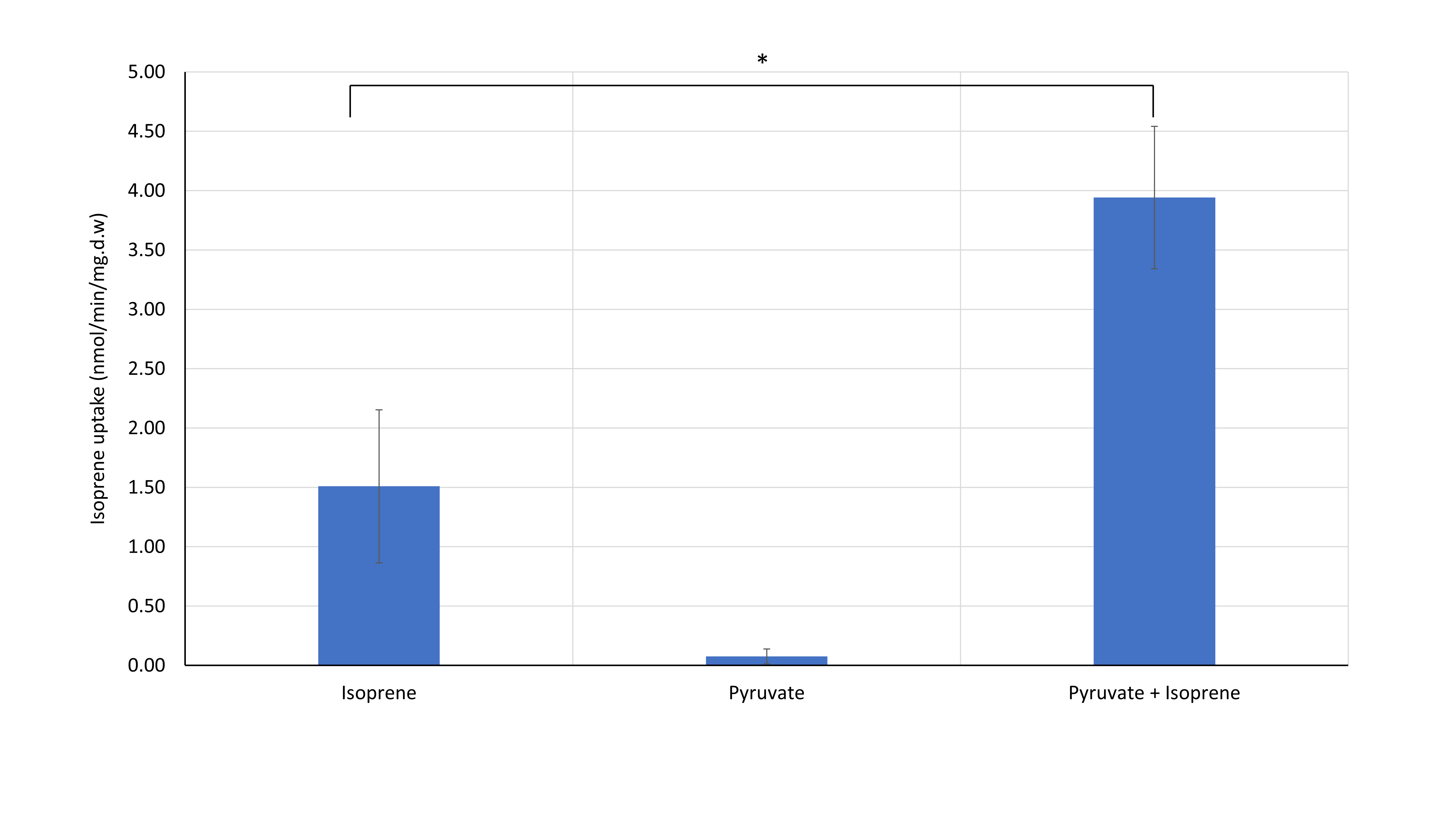

Supplement: Supplementary file 7 — Figure S7 Isoprene oxidation by Variovorax sp. WS11 grown in the presence of 1% (v/v) isoprene, 10 mM pyruvate, or a combination of 1% (v/v) isoprene and 10 mM pyruvate, measured as nmol isoprene consumed minute−1 mg dry weight−1. Error bars represent the standard deviation about the mean (n = 3). An asterisk denotes a statistically significant difference (p ≤ 0.05) between the indicated conditions, determined by students t‐test. [file EMI-24-5151-s016.tif]

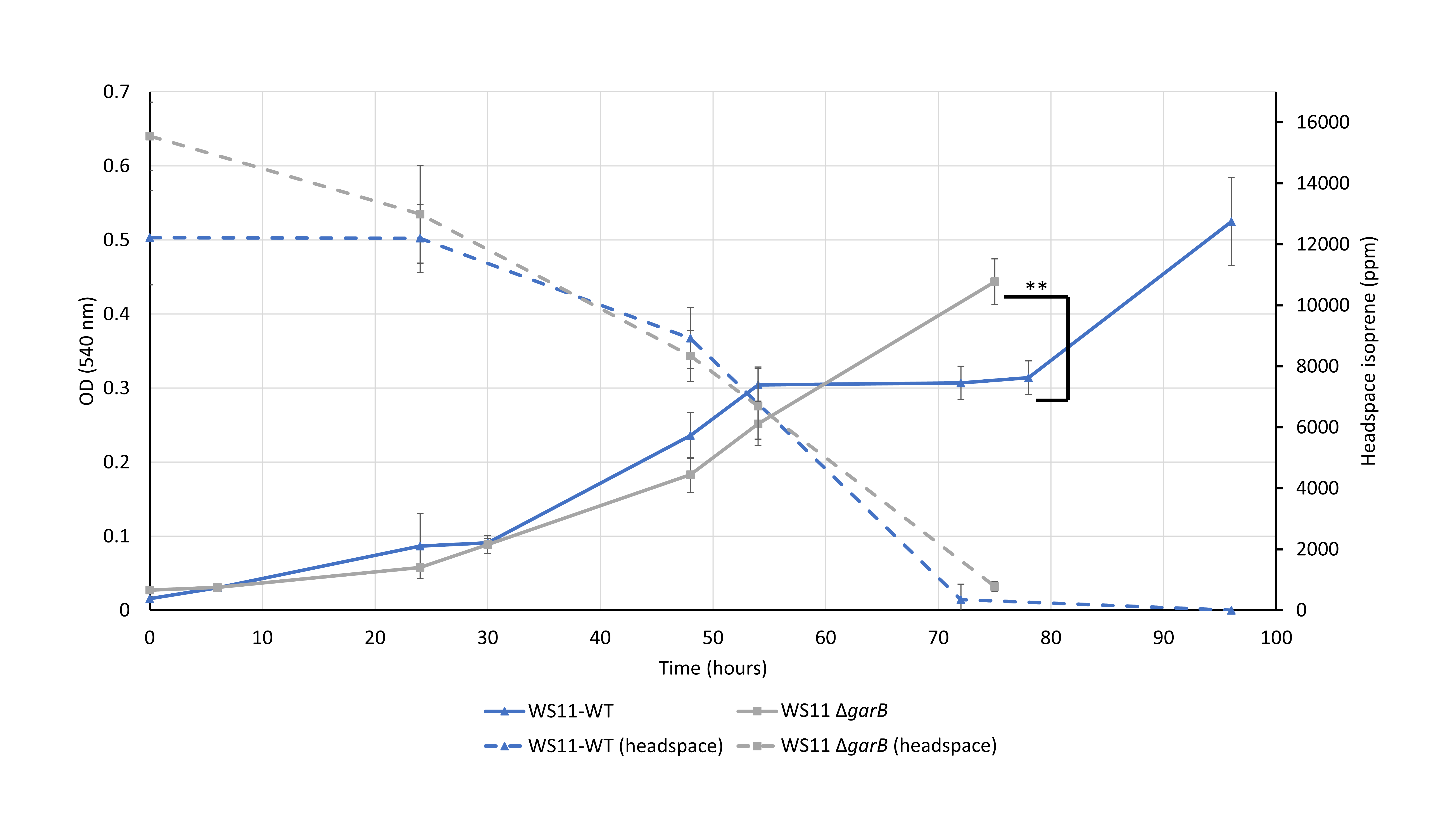

Supplement: Supplementary file 8 — Figure S8 Growth of Variovorax sp. WS11 ΔgarB on 1% (v/v) isoprene, compared to wild‐type Variovorax sp. WS11. Error bars represent the standard deviation about the mean (n = 3). An asterisk denotes a statistically significant difference between the indicated conditions (p ≤ 0.01). [file EMI-24-5151-s021.tif]

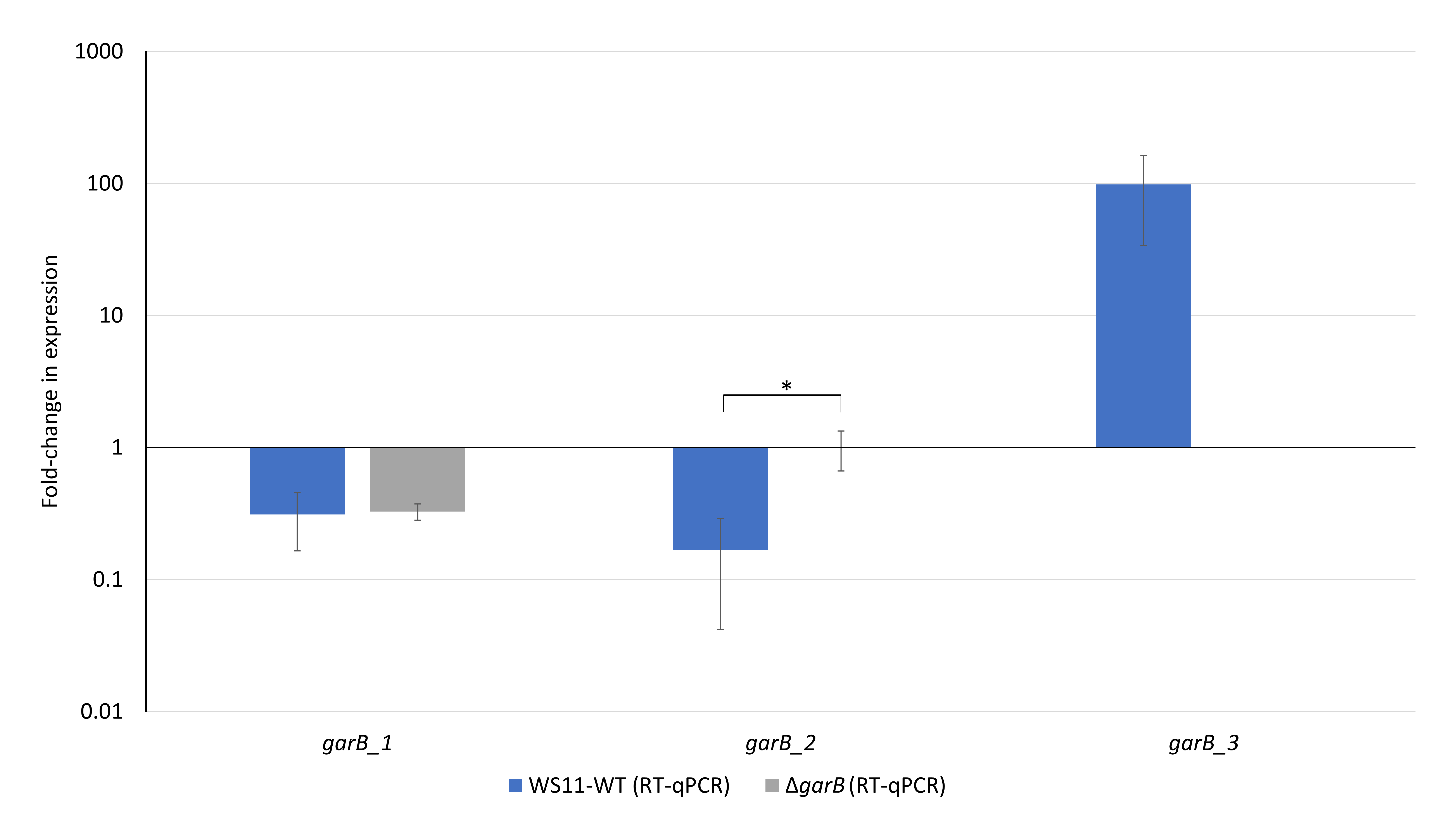

Supplement: Supplementary file 9 — Figure S9 Fold‐change in expression of garB_1, garB_2, and garB_3 by Variovorax sp. WS11 (wild‐type) and Variovorax sp. WS11 ΔgarB, during growth on isoprene compared to growth on succinate, determined by RT‐qPCR, relative to the expression of rpoB. The fold‐change in expression of garB_3 determined by RNA‐seq was calculated relative to timepoint 0. Error bars represent the standard deviation about the mean (n = 3). An asterisk (*) denotes a statistically significant difference between the indicated conditions (p ≤ 0.05). [file EMI-24-5151-s014.tif]

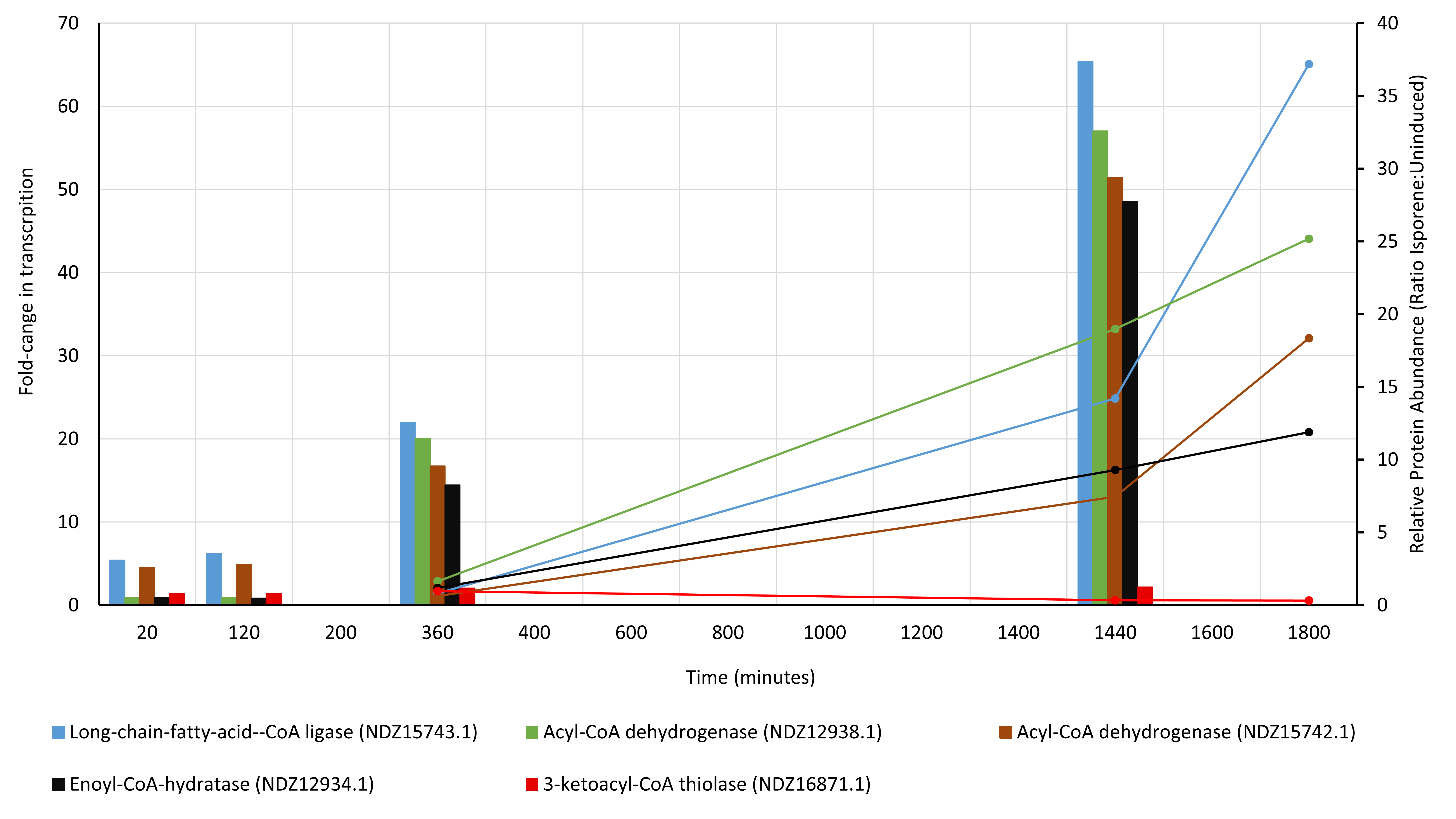

Supplement: Supplementary file 10 — Figure S10 Isoprene‐induced changes in the transcription of typical β‐oxidation genes (bars), and the relative protein abundance of the respective gene products (lines). [file EMI-24-5151-s020.tif]

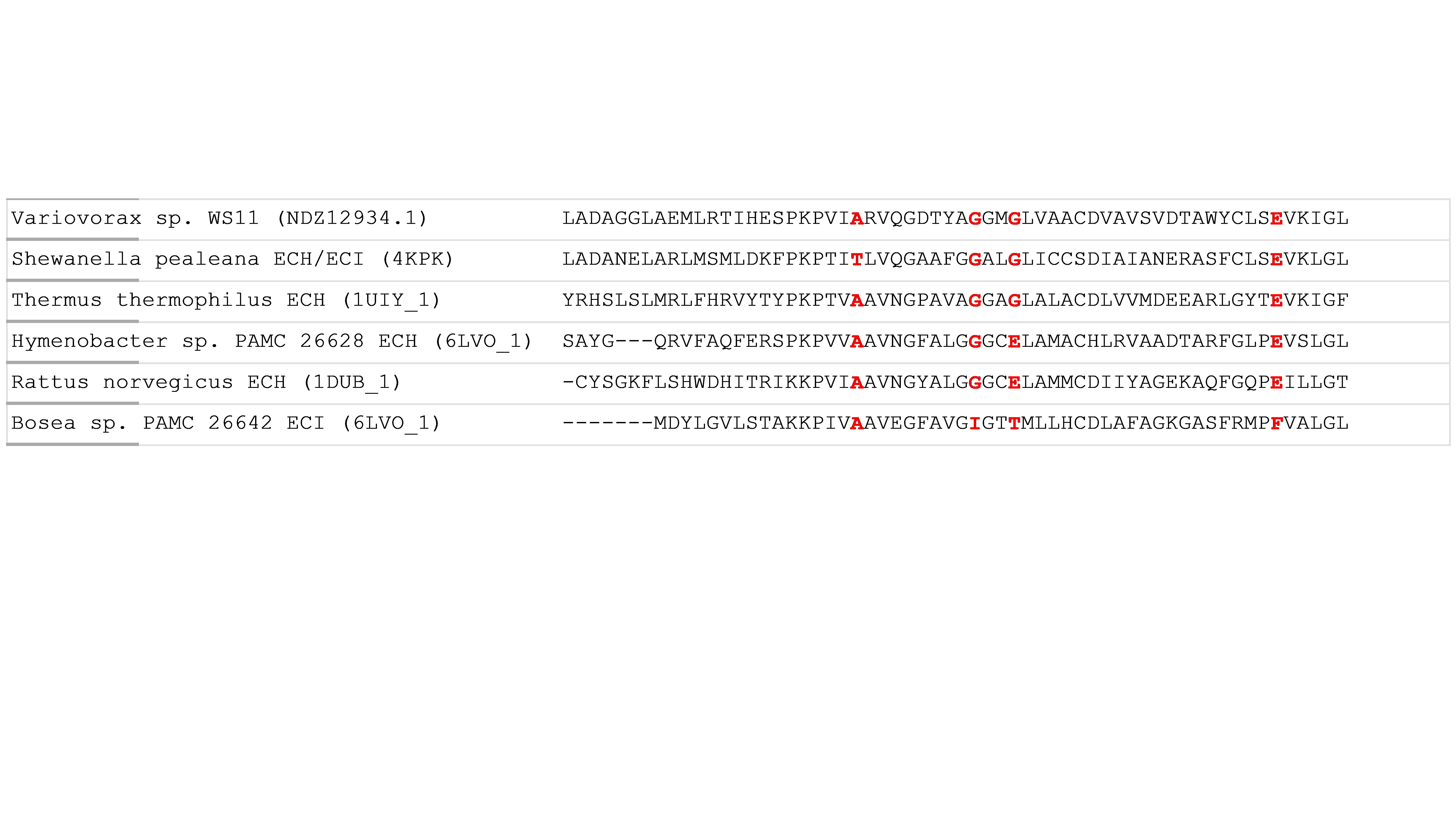

Supplement: Supplementary file 11 — Figure S11 Alignment of the deduced amino acid sequence of NDZ12934.1 (ECH) with published ECH and ECI sequences, using the MAFFT online multiple sequence alignment tool with default parameters (Katoh et al., 2019). Conserved residues [Ala98, Gly142, Glu144, Glu164, based on alignment against the Rattus norvegicus ECH (Padavattan et al., 2021)] are highlighted in red. Glu144 has been substituted with a glycine residue in Variovorax sp. WS11. Where available, protein databank identifiers have been included in brackets. [file EMI-24-5151-s012.tif]

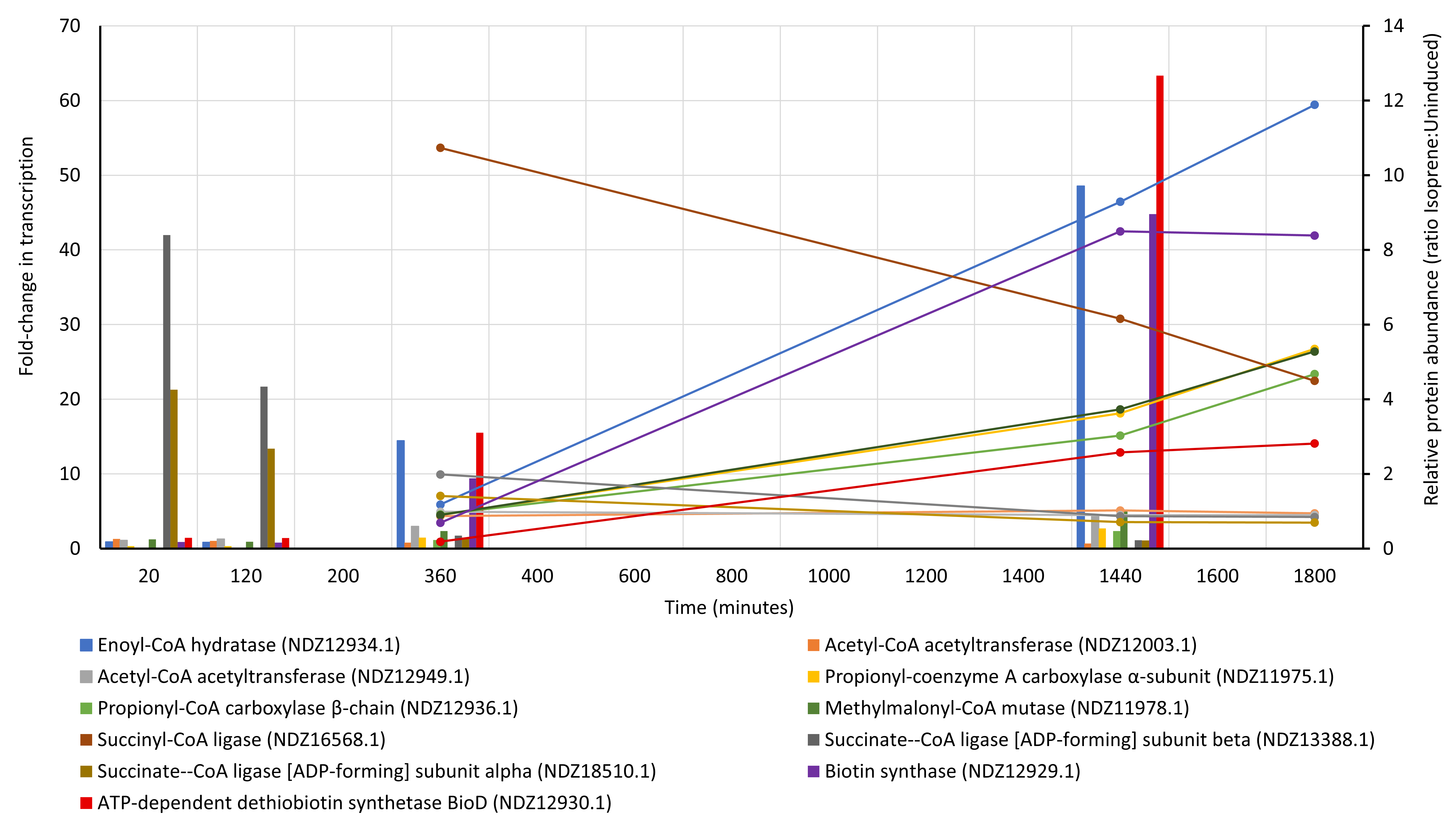

Supplement: Supplementary file 12 — Figure S12 Isoprene‐induced changes in the expression of transcribed genes (bars) and translated gene products (lines) with predicted roles in the isoprene metabolic pathway (Figure 4), subsequent to GMBA formation by IsoH. [file EMI-24-5151-s002.tif]

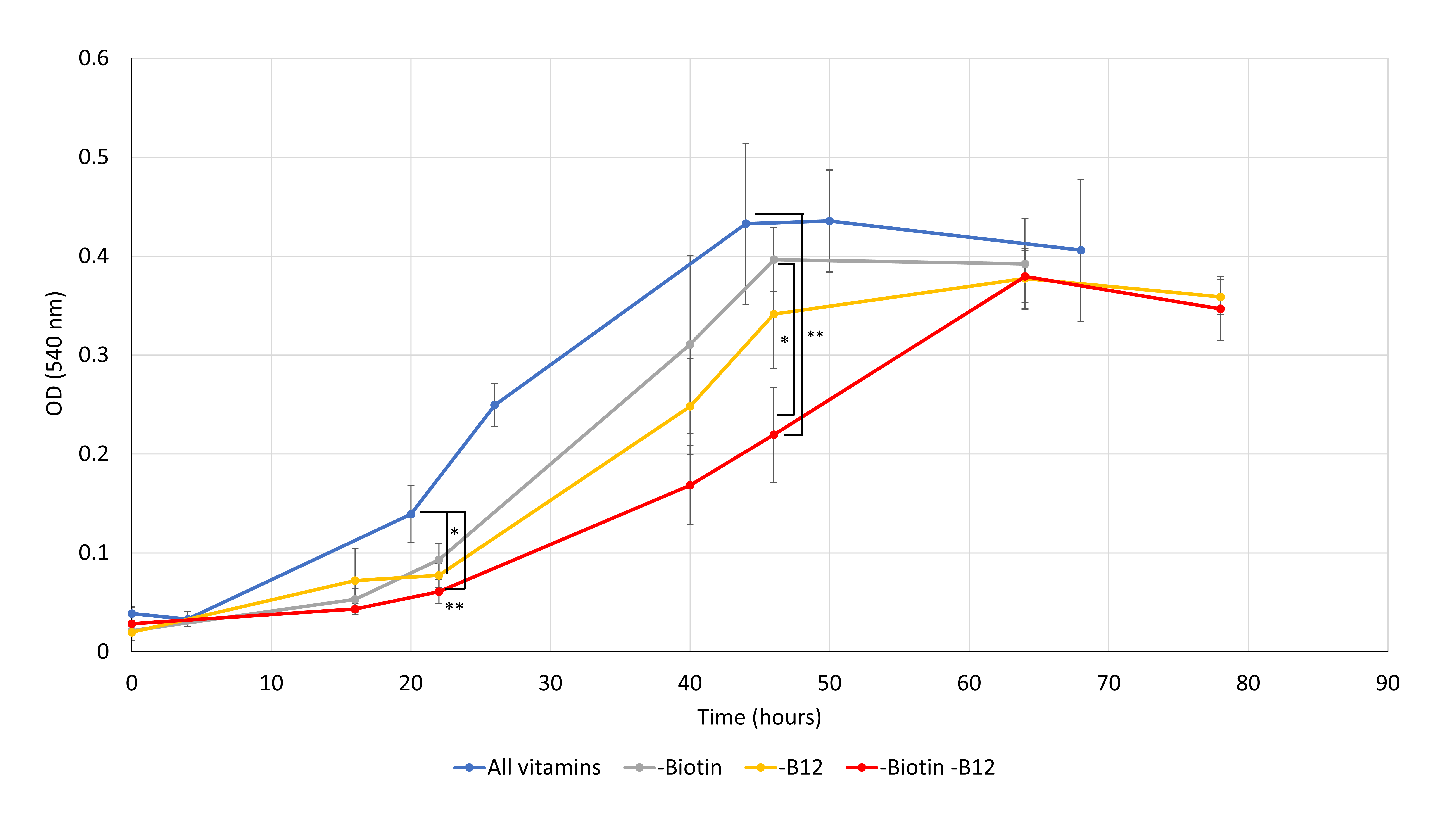

Supplement: Supplementary file 13 — Figure S13 Growth of Variovorax sp. WS11 on 1% (v/v) isoprene in the presence of a complete vitamin solution, in the absence of biotin (−biotin), in the absence of vitamin B12 (‐B12), or in the absence of both biotin and vitamin B12 (‐Biotin ‐B12). Error bars represent the standard deviation about the mean (n = 3). An asterisk denotes a statistically significant difference between the indicated conditions (*p ≤ 0.05, **p ≤ 0.01). [file EMI-24-5151-s010.tif]

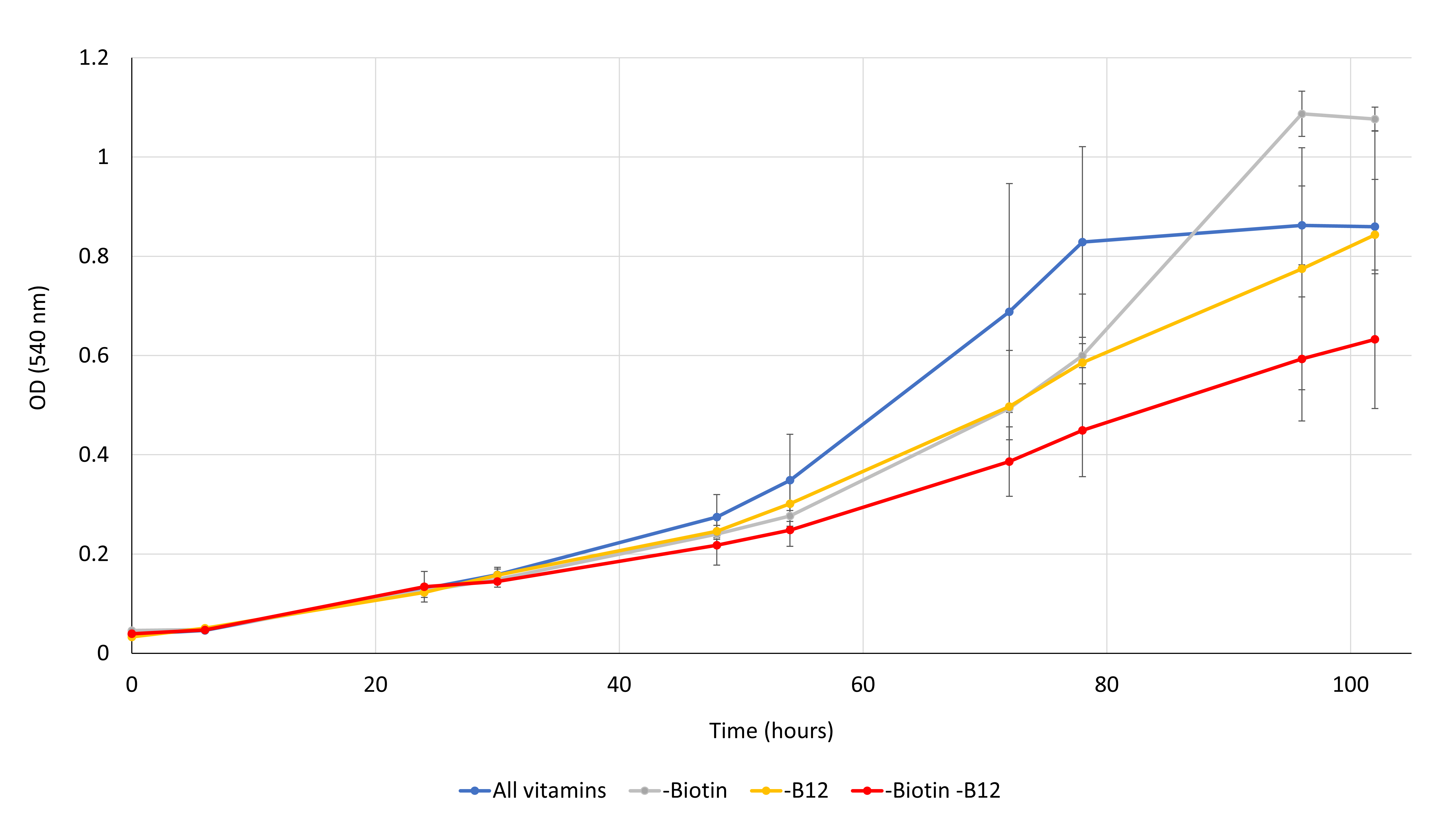

Supplement: Supplementary file 14 — Figure S14 Growth of Variovorax sp. WS11 on 10 mM propionate in the presence of all vitamins, in the absence of biotin (−biotin), in the absence of vitamin B12 (‐B12), or in the absence of both biotin and vitamin B12 (‐Biotin ‐B12). Error bars represent the standard deviation about the mean (n = 3). [file EMI-24-5151-s004.tif]

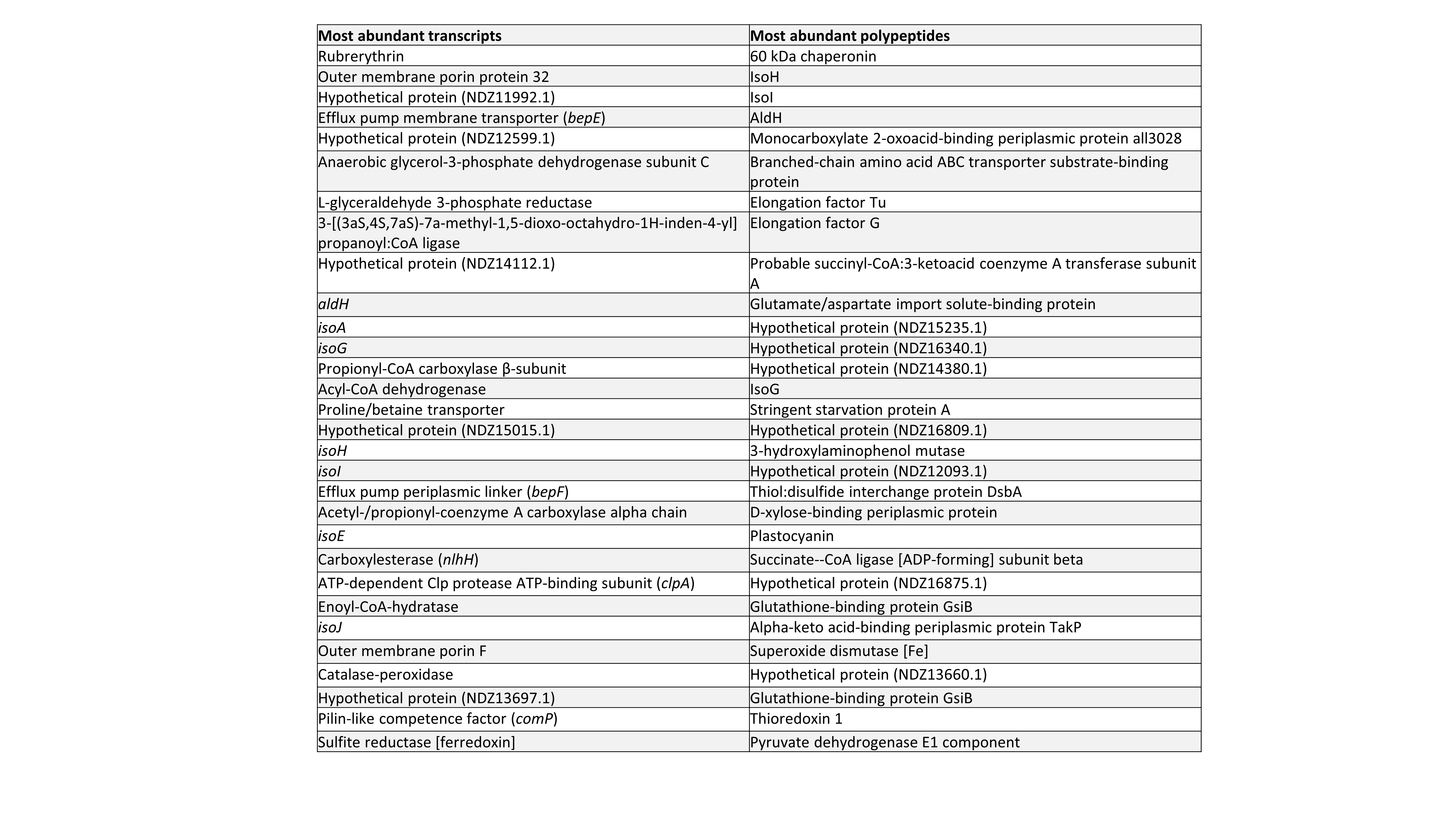

Supplement: Supplementary file 19 — Table S1 Top 30 most abundant transcripts and polypeptides after 24 hours' growth on isoprene, calculated as the fold‐change in transcripts and ratio of abundance of polypeptides, compared to the respective timepoint 0 samples. [file EMI-24-5151-s017.tif]

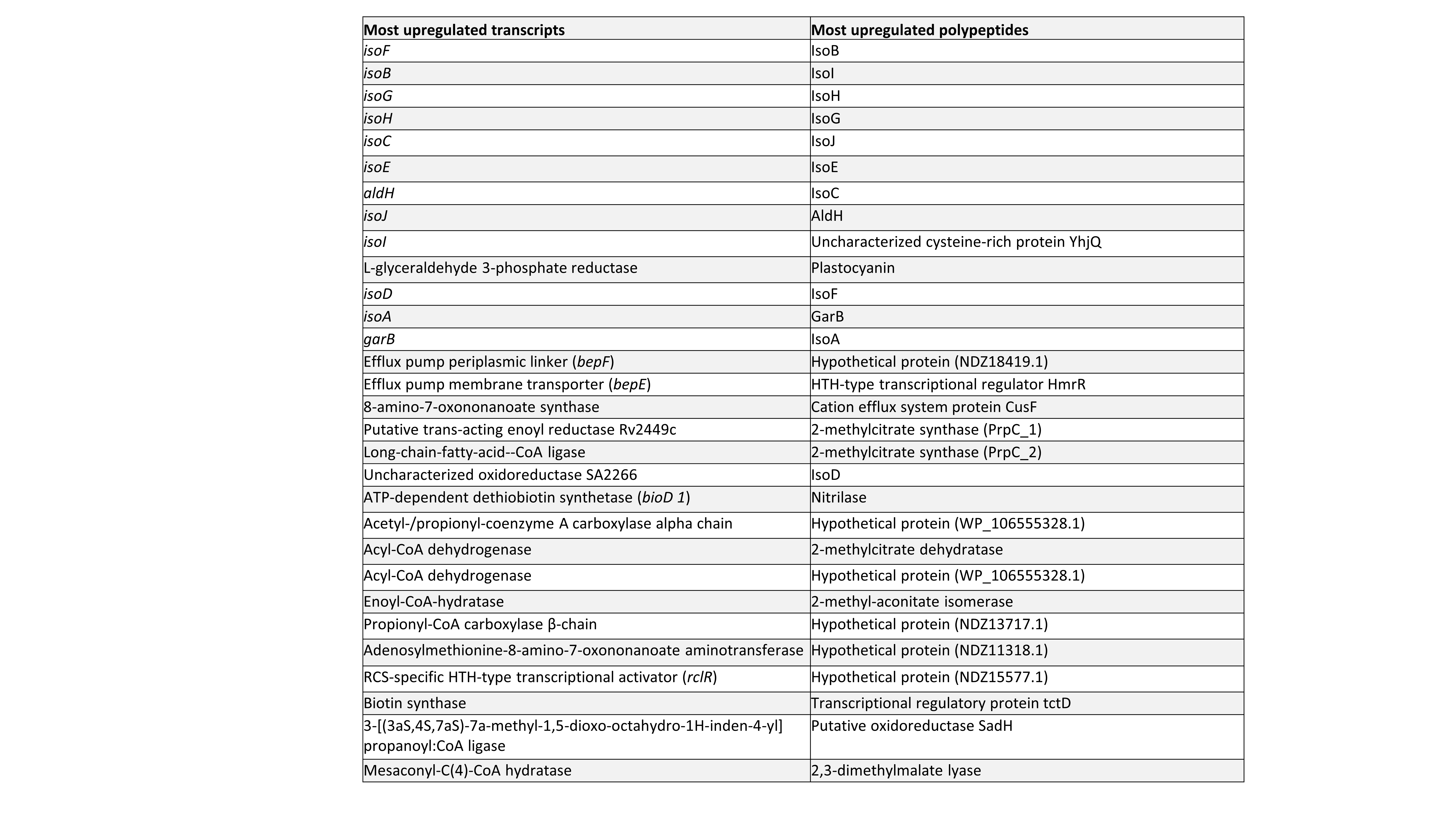

Supplement: Supplementary file 20 — Table S2 Top 30 most upregulated transcripts and polypeptides after 24 hours' growth on isoprene, calculated as the fold‐change in transcripts and the ratio of abundance of polypeptides, compared to the respective timepoint 0 samples. [file EMI-24-5151-s018.tif]

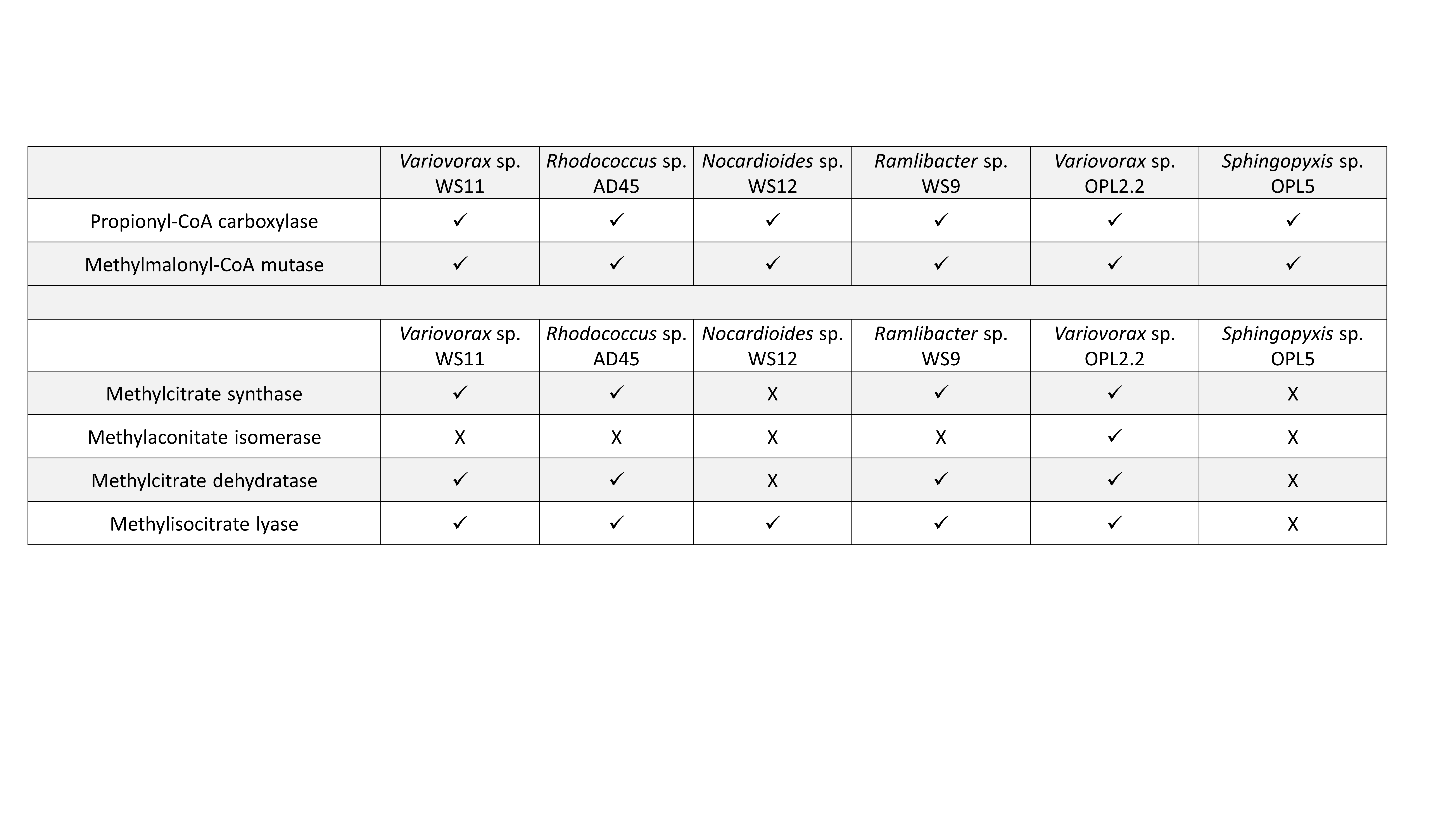

Supplement: Supplementary file 21 — Table S3 Presence (✓) or absence (X) of genes involved in the methylcitrate pathway and propionyl‐CoA assimilation pathways in isoprene degrading bacteria. [file EMI-24-5151-s011.tif]

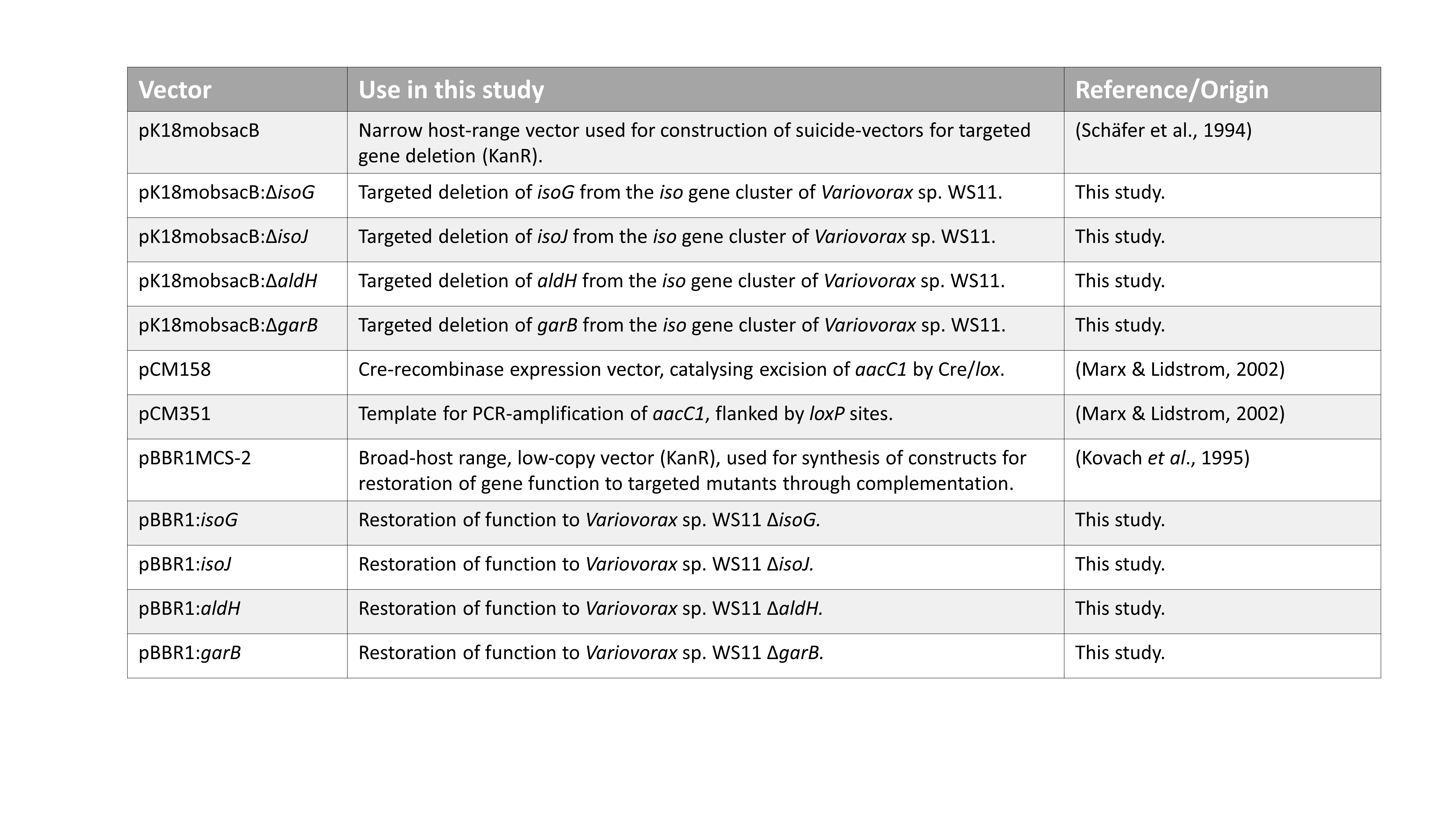

Supplement: Supplementary file 22 — Table S4 Vectors used in this study. [file EMI-24-5151-s008.tif]

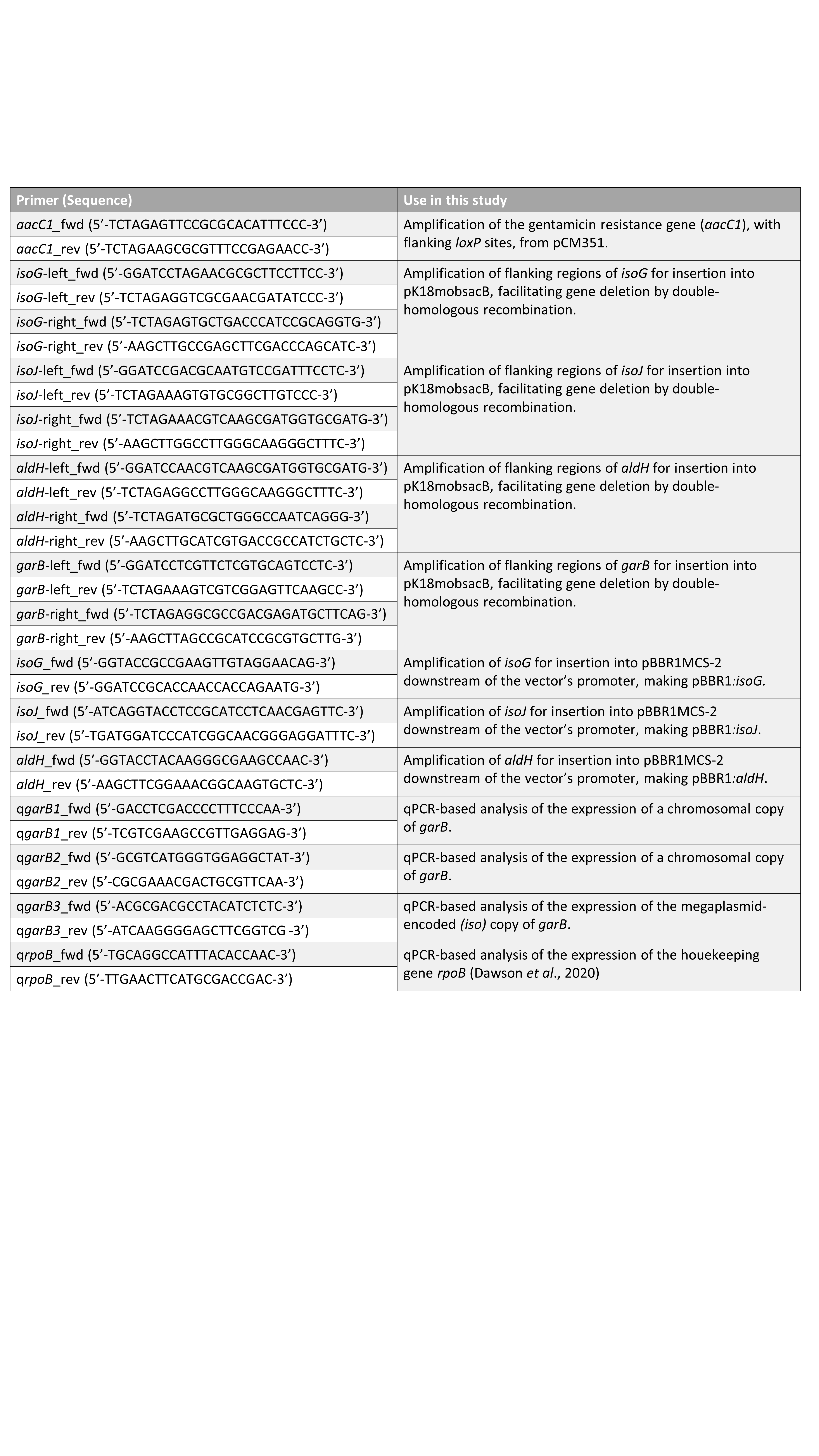

Supplement: Supplementary file 23 — Table S5 Primers used in this study. [file EMI-24-5151-s023.tif]
