# Supplementary material for: ‘Omics‐guided prediction of the pathway for metabolism of isoprene by Variovorax sp. WS11
Source: Environ Microbiol. 2022 Aug 5;24(11):5151–64. doi: 10.1111/1462-2920.16149 (PMC9804861; doi:10.1111/1462-2920.16149)
Supplement: Supplementary file 18 — Appendix S4 Supporting Information. [file EMI-24-5151-s019.docx]

Supplementary Information

Dawson, R.A., Rix, G.D., Crombie, A.T. and Murrell, J.C.*

*Corresponding author.

**Supplementary Materials and Methods**

**Analysis of the transcriptome of isoprene-grown *Variovorax* sp. WS11**

*Preparation of total RNA samples*

*Variovorax* sp. WS11 was grown in Ewers medium using 10 mM succinate as the sole source of carbon and energy to an OD_540_ of 0.6. Cultures were centrifuged at 4,000 *g*  for 10 minutes at 4 ^o^C, the supernatant was discarded, and the cell pellets were resuspended in an equal volume of fresh Ewers medium with no carbon source. Cell suspensions were starved for 1 hour at 30 ^o^C with shaking at 160 rpm with the aim of depleting intracellular stores of carbon which might interfere with the induction of gene expression. Starved cultures were amended with 10 mM succinate, 0.01% (w/v) epoxyisoprene, 1% (v/v) isoprene, or no substrate (starved control) and incubated at 30 ^o^C with shaking at 160 rpm. 10 ml aliquots of cells were removed by sterile syringe at the following times: no substrate/succinate (1 hour, 2 hours, 6 hours, 24 hours), isoprene (20 minutes, 2 hours, 6 hours, 24 hours), epoxyisoprene (10 minutes, 20 minutes, 2 hours, 4 hours). Aliquots were immediately treated with 2 volumes of RNAprotect (Qiagen, Hilden, Germany) according to the manufacturer’s instructions and stored at -80 ^o^C for no longer than 5 days. Protected cell pellets were thawed and total RNA was extracted using TRIzol reagent (Thermo Fisher Scientific, Waltham, MA, USA) according to the manufacturer’s instructions. Contaminating DNA was removed from the RNA by a single treatment with RNase-free DNase (Qiagen) according to the manufacturer’s instructions, followed by purification using an RNeasy minikit (Qiagen). Polymerase chain reaction (PCR) was used to confirm the lack of contaminating DNA, using primers designed to amplify the 16S ribosomal RNA (rRNA) gene (Lane, 1991).

*Sequencing, read alignment, and quantification*

RNA samples were used for library preparation by Novogene UK (Cambridge, UK). The directional library was prepared using NEBNext® UltraTM Directional RNA Library Prep Kit for Illumina® (NEB, USA) following the manufacturer’s protocol. Indices were included to multiplex multiple samples. Briefly, mRNA was purified from total RNA using poly-T oligo-attached magnetic beads. After fragmentation, the first strand cDNA was synthesized using random hexamer primers followed by the second strand cDNA synthesis. The strand-specific library was ready after end repair, A-tailing, adapter ligation, size selection, and USER enzyme digestion. After amplification and purification, insert size of the library was validated on an Agilent 2100 and quantified using quantitative PCR (Q-PCR). Libraries were then sequenced on Illumina NovaSeq 6000 S4 flowcell with PE150 according to results from library quality control and expected data volume.

**Analysis of the proteome of isoprene-grown *Variovorax* sp. WS11**

*Preparation of total protein samples*

*Variovorax* sp. WS11 was grown in 1 L Ewers medium in a 2 L flask with 10 mM succinate to an OD_540_ of 0.6, then centrifuged at 7,000 rpm for 10 minutes at 4 ^o^C in an Avanti J-20 high-speed centrifuge (Beckman Coulter, Pasadena, CA, USA) using a JLA 8.1000 rotor (Beckman Coulter). The supernatant was discarded and the cell pellet was washed in an equal volume of chilled Ewers medium. Cells were centrifuged as described above and resuspended in an equal volume of fresh Ewers medium without a carbon source, then starved for 1 hour at 30 ^o^C with shaking at 160 rpm. Two replicates of 200 ml cells were removed (timepoint 0) and stored as described below. The remaining cells were amended with 10 mM succinate or 1% (v/v) isoprene, using two biological replicates per condition, and 200 ml aliquots were removed after 6 hours, 24 hours, and 30 hours. Aliquots were centrifuged at 7,000 rpm for 10 minutes at 4 ^o^C, washed in an equal volume of ice-cold HEPES 50 mM HEPES (pH 7.0), and centrifuged as before. Cell pellets were resuspended in 1 ml of ice-cold breakage buffer (50 mM HEPES, pH 7.0, 10 mM dithiothreitol (DTT), 200 mM NaCl, SIGMA*FAST* Protease Inhibitor Cocktail Tablets (EDTA-Free, Sigma Aldrich, St Louis, USA)). Protease inhibitors were added to the breakage buffer according to the manufacturer’s instructions. Cells were broken by three passages through a pre-chilled French Pressure Mini at 20,000 psi (137 MPa), medium ratio. Cell debris was removed by spinning in a benchtop centrifuge at 17,000 g for 30 minutes at 4 ^o^C. Cell extracts were stored at -20 ^o^C until ready for analysis, which was run at the Proteomics Facility, John Innes Centre (Norwich, UK).

*Tandem Mass Tag (TMT) labelling and analysis by Orbitrap mass spectrometer*

Protein precipitation was achieved using 5 volumes of acetone (Nickerson and Doucette, 2020) and the resulting pellets were dissolved in 300 µl of 2.5% sodium deoxycholate (SDC; Merck). 100 µg of protein per sample (determined by bicinchoninic acid (BCA) assay) were reduced, alkylated, and digested with trypsin in the presence of 0.2 M EPPS buffer and 2.5% SDC according to standard procedures. Post-digest, the SDC was precipitated by adjusting to 0.2% trifluoroacetic acid (TFA), and the clear supernatant subjected to C18 solid phase extraction (SPE) (OMIX tips; Agilent). TMT labelling was performed using a TMTpro™ 16plex kit (Lot VI306840, ThermoFisher Scientific) according to the manufacturer’s instructions with slight modifications; the dried peptides were dissolved in 90 µl of 0.2 M EPPS buffer (MERCK)/10% acetonitrile, and 250 µg TMT reagent was added (dissolved in 22 µl of acetonitrile). Samples were assigned to the TMT channels in order avoiding channel leakage between different samples, if possible (Brenes *et al.*, 2019). After labelling, aliquots of 2 µl from each sample were combined, desalted, and analysed on the mass spectrometer (see below) to check labelling efficiency and estimate total sample abundances. The main sample aliquots were combined correspondingly and desalted using a C18 Sep-pak cartridge (Waters, Wilmslow, UK). The eluted peptides were dissolved in 500 µl of 10 mM NH_4_HCO_3_ and fractionated by high pH reversed-phase high performance liquid chromatography (HPLC). Using an ACQUITY Arc Bio System (Waters), the samples were loaded to a Kinetex® 5 µm EVO C18 100 Å liquid chromatography (LC) Column 250 x 4.6 mm (Phenomenex). Fractionation was performed with the following gradient of solvents A (water), B (acetonitrile), and C (25 mM NH_4_HCO_3_) at a flow rate of 1 ml min^-1^: solvent C was kept at 10% throughout the gradient; solvent B: 0-5 min: 5%, 5-10 min: 5-10%, 10-80 min: 10-50%, 80-90 min: 50-80%, followed by 5 min at 80% B and re-equilibration to 5%. Fractions of 1 ml were collected, dried down, and concatenated to produce 24 final fractions for mass spectrometry (MS) analysis.

Aliquots were analysed by nanoLC-MS/MS on an Orbitrap Eclipse™ Tribrid™ mass spectrometer coupled to an UltiMate® 3000 RSLCnano LC system (Thermo Fisher Scientific, Hemel Hempstead, UK). The samples were loaded onto a trap column (nanoEase M/Z Symmetry C18 Trap Column, Waters) with 0.1% TFA at 15 µl min^-1^ for 3 min. The trap column was then switched in-line with the analytical column (nanoEase M/Z column, HSS C18 T3, 1.8 µm, 100 Å, 250 mm x 0.75 µm, Waters) for separation using the following gradient of solvents A (water, 0.1% formic acid) and B (80% acetonitrile, 0.1% formic acid) at a flow rate of 0.2 µl min^-1^ : 0-3 min 3% B (parallel to trapping); 3-10 min linear increase B to 13%; 10-105 min increase B to 50%; followed by a ramp to 99% B and re-equilibration to 3% B. Data were acquired with the following parameters in positive ion mode: MS1/OT: resolution 120K, profile mode, mass range *m/z* 400-1800, AGC target 100%, max inject time 50 ms; MS2/IT: data dependent analysis with the following parameters: CID fragmentation of top10 in IT normal mode, CE = 30, quadrupole isolation window 0.7 Da, charge states 2-5, threshold 1.0e4, AGC target 100%, max. inject time 70 ms, dynamic exclusion 1 count/7 s/±7 ppm, centroid mode; MS3 synchronous precursor selection (SPS): 10 SPS precursors, isolation window 0.7 Da, higher-energy C-trap dissociation (HCD) fragmentation with CE=50, Orbitrap Turbo TMT and TMTpro resolution 15K, AGC target 200%, max inject time 120 ms, Real Time Search: protein fasta database *Variovorax* sp. WS11 (JAAGOW000000000_translated_cds.faa, number of entries: 8058), 1 missed cleavage, oxidation (M) as variable, carbamidomethyl (C) and TMTpro 16plex as fixed modifications, Xcorr = 1, dCn = 0.05, tolerance 6 ppm.

The acquired raw data were processed and quantified in Proteome Discoverer 2.4.1.15 (Thermo) using the incorporated search engine Sequest HT and the Mascot search engine (Matrix Science, London, UK; Mascot version 2.7.0). The processing workflow included recalibration of MS1 spectra (RC), reporter ion quantification by most confident centroid (20 ppm), fasta database *Variovorax* sp. WS11 (JAAGOW000000000_translated_cds.faa, number of entries: 8058) and common contaminants, precursor/fragment tolerance 6 ppm/0.6 Da, variable/fixed modifications were oxidation (M)/carbamidomethyl (C) and TMTpro™ 16plex. The consensus workflow included the following parameters: unique peptides (protein groups), intensity-based abundance, TMT channel correction values applied (VI306840), co-isolation/SPS matches thresholds 50%/80%, normalisation on total peptide abundances, protein abundance-based ratio calculation, missing values imputation by low abundance resampling, two replicates each with nested design, hypothesis testing by *t*-test (background based), adjusted p-value calculation by BH-method.


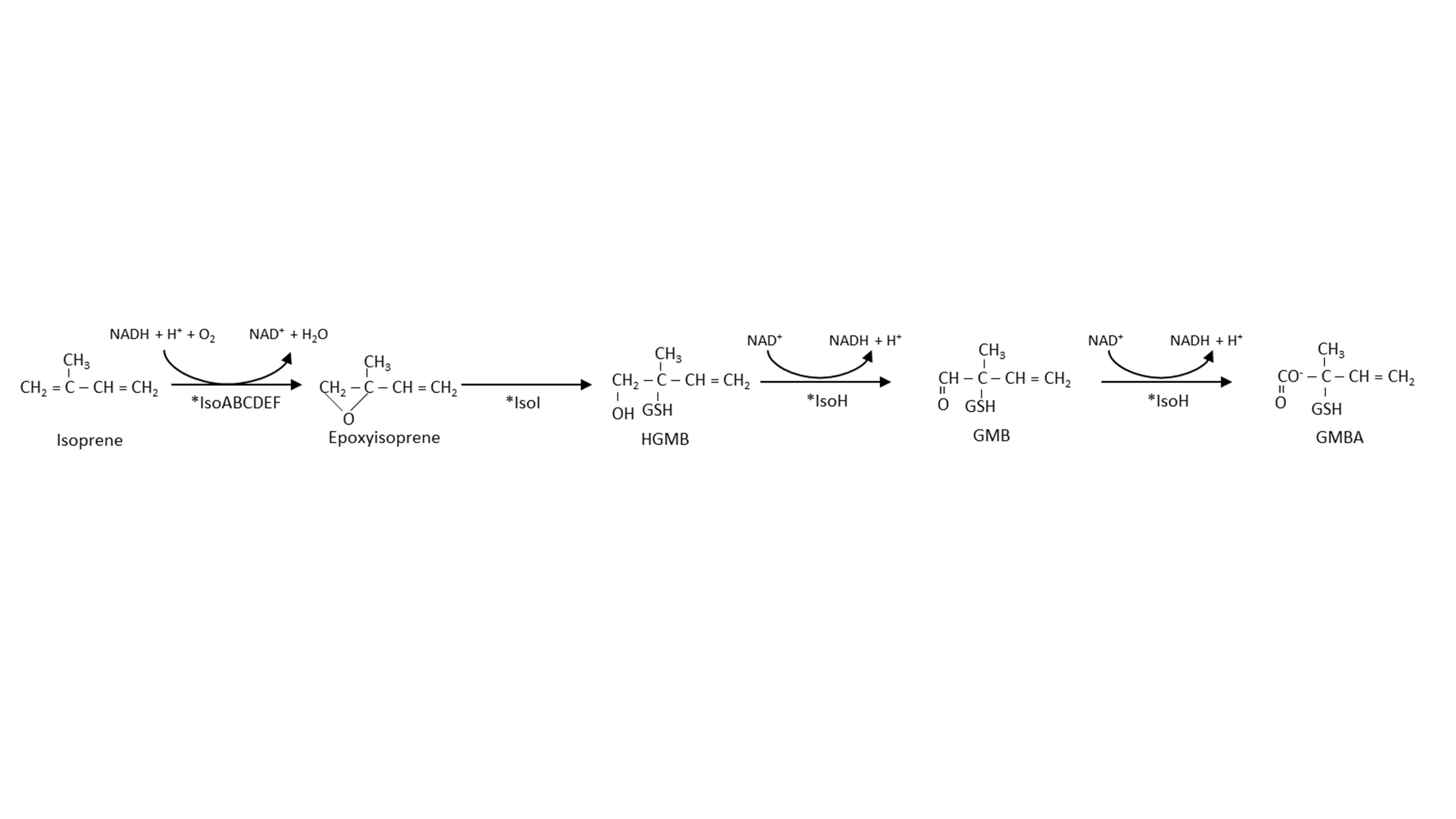


Figure S1. Confirmed steps of isoprene metabolism, adapted from van Hylckama Vlieg *et al*. (2000). Steps which are catalysed by enzymes are marked by an asterisk. IsoABCDEF: isoprene monooxygenase. IsoI: glutathione *S*-transferase. IsoH: dehydrogenase.


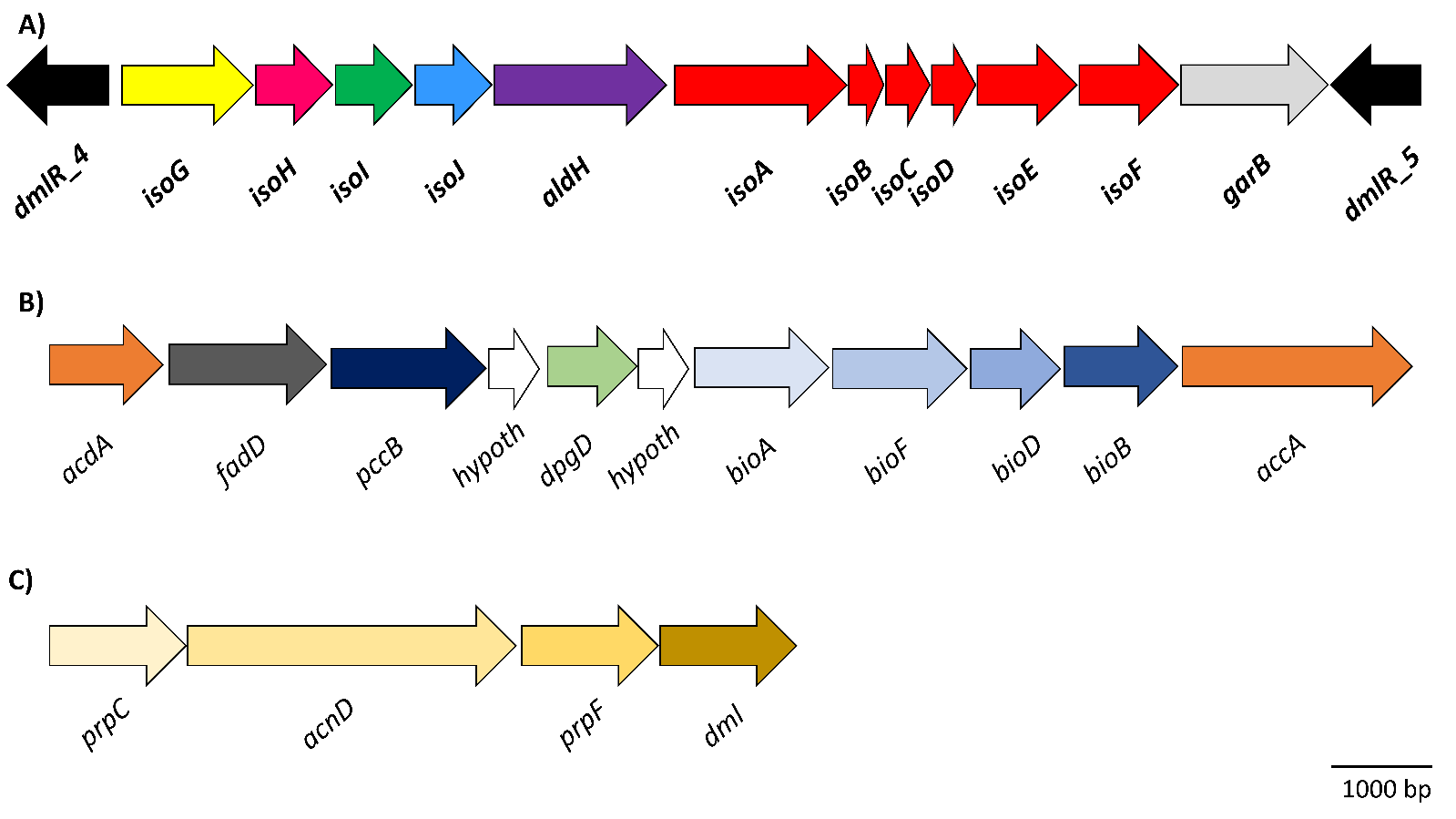


Figure S2. A) *iso* metabolic gene cluster encoded on Megaplasmid 1 of *Variovorax* sp. WS11 (taken from Dawson *et al.*, 2020). B) Putative isoprene-induced gene cluster with predicted roles in β-oxidation. *acdA*: acyl-CoA dehydrogenase (NDZ12938.1), *fadD*: propionyl-CoA ligase (NDZ12937.1), *pccB*: propionyl-CoA carboxylase, β-chain (NDZ12936.1), *hypoth*: hypothetical protein (NDZ12935.1, NDZ12933.1), *dpgD*: enoyl-CoA hydratase (NDZ12934.1), *bioA*: adenosylmethionine-8-amino-7-oxononanoate aminotransferase (NDZ12932.1), *bioF*: 8-amino-7-oxononanoate synthase (NDZ12931.1), *bioD*: ATP-dependent dethiobiotin synthetase (NDZ12930.1), *bioB*: biotin synthase (NDZ12929.1), *accA*: acetyl-/propionyl-CoA carboxylase α-chain (NDZ12928.1). C) Putative methylcitrate pathway gene cluster. *prpC*: 2-methylcitrate synthase (NDZ17600.1), *acnD*: 2-methylisocitrate dehydratase (NDZ17599.1), *prpF*: 2-methylaconitate isomerase (NDZ17598.1), *dml*: 2,3-dimethylmalate lyase (NDZ17597.1).


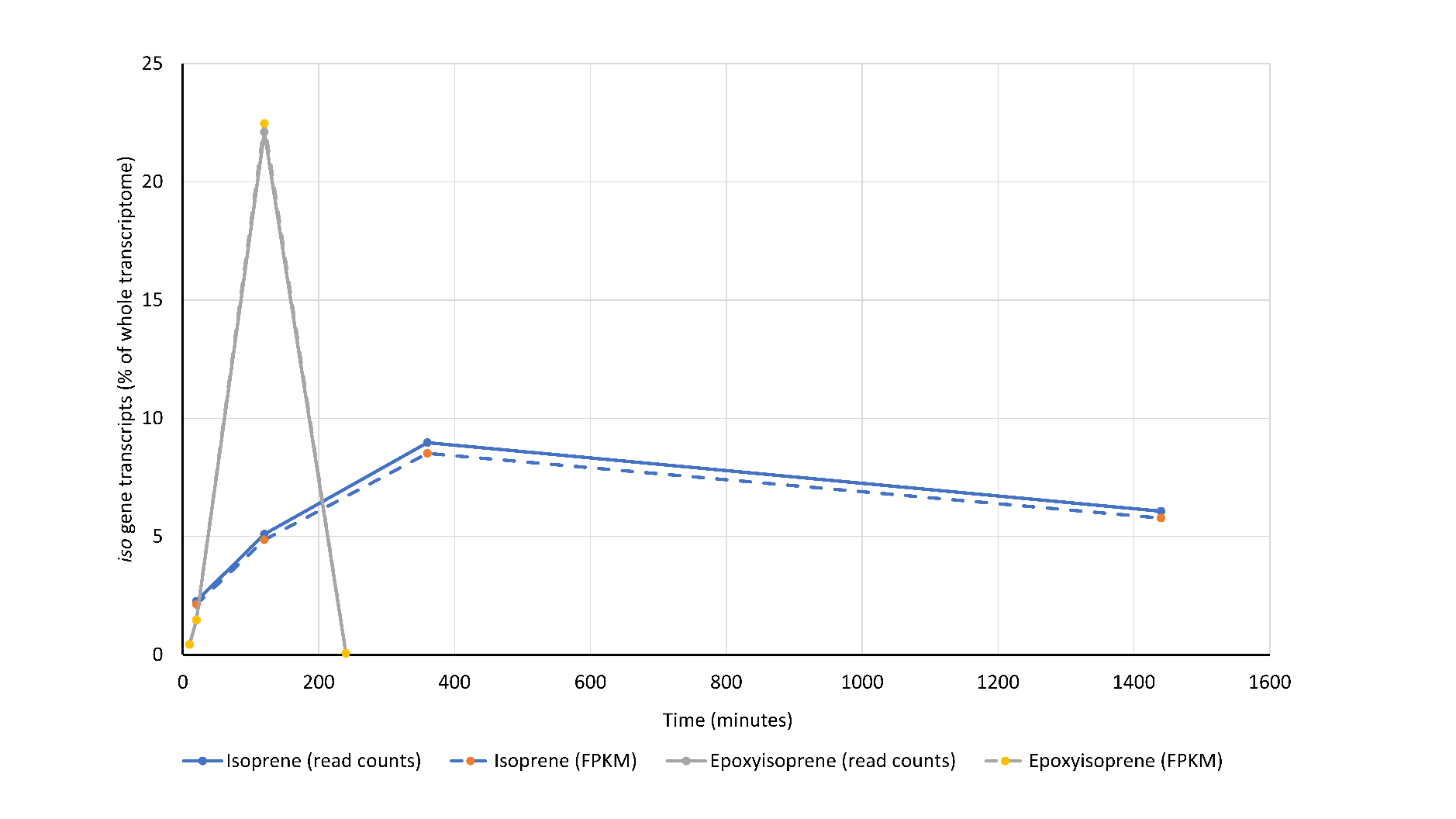


Figure S3. *iso* metabolic gene transcripts (normalised read counts vs. fragments per kilobase million) as a percentage of all detected transcripts during growth on isoprene or incubation with epoxyisoprene, measured over time.


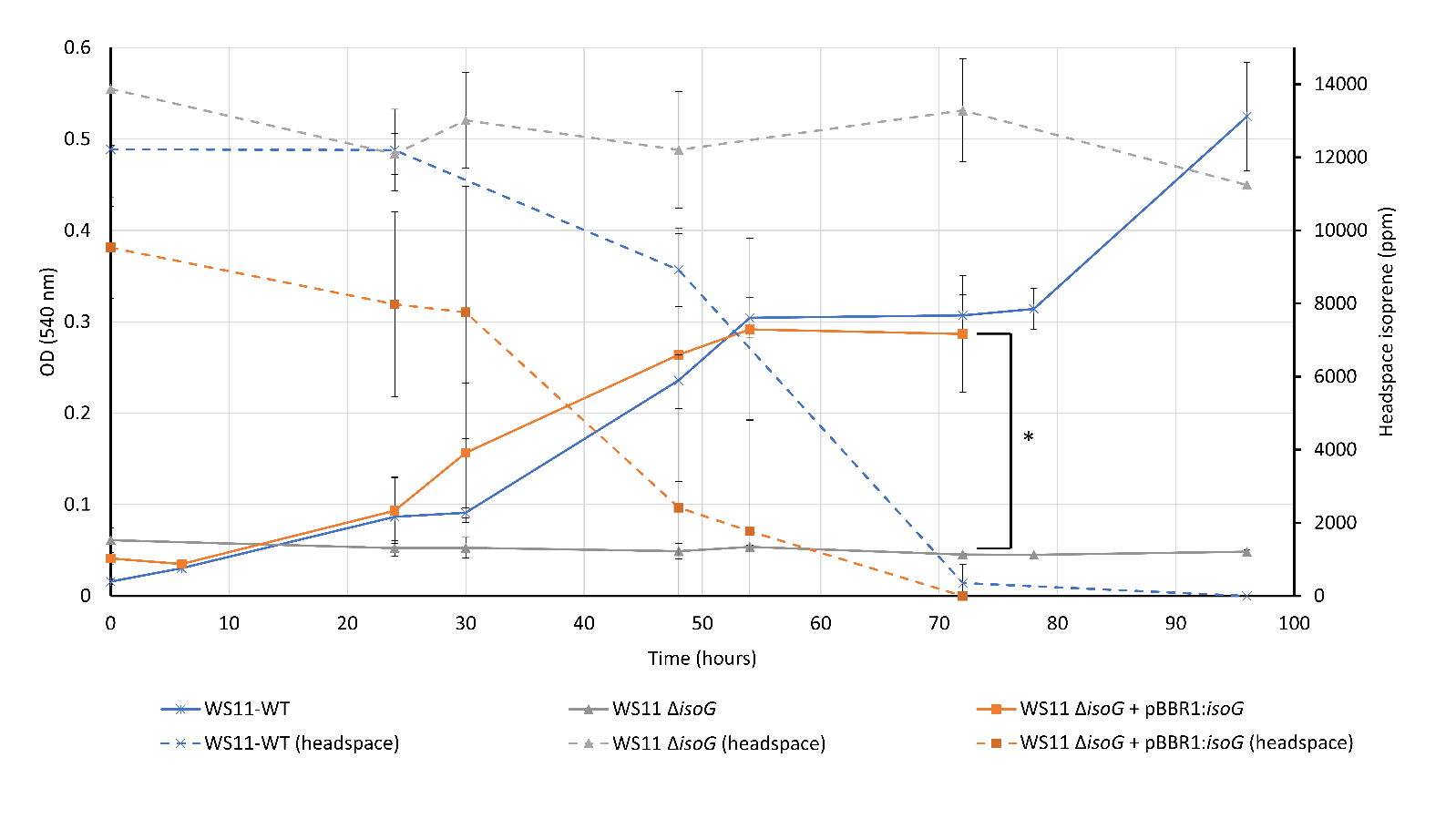


Figure S4. Growth of *Variovorax* sp. WS11 Δ*isoG* on 1% (v/v) isoprene, compared with growth of *Variovorax* sp. WS11 Δ*isoG* transformed with pBBR1:*isoG*. Error bars represent the standard deviation about the mean (n=3). An asterisk (*) denotes a statistically significant difference between the indicated data (p ≤ 0.05), determined by student’s *t*-test.


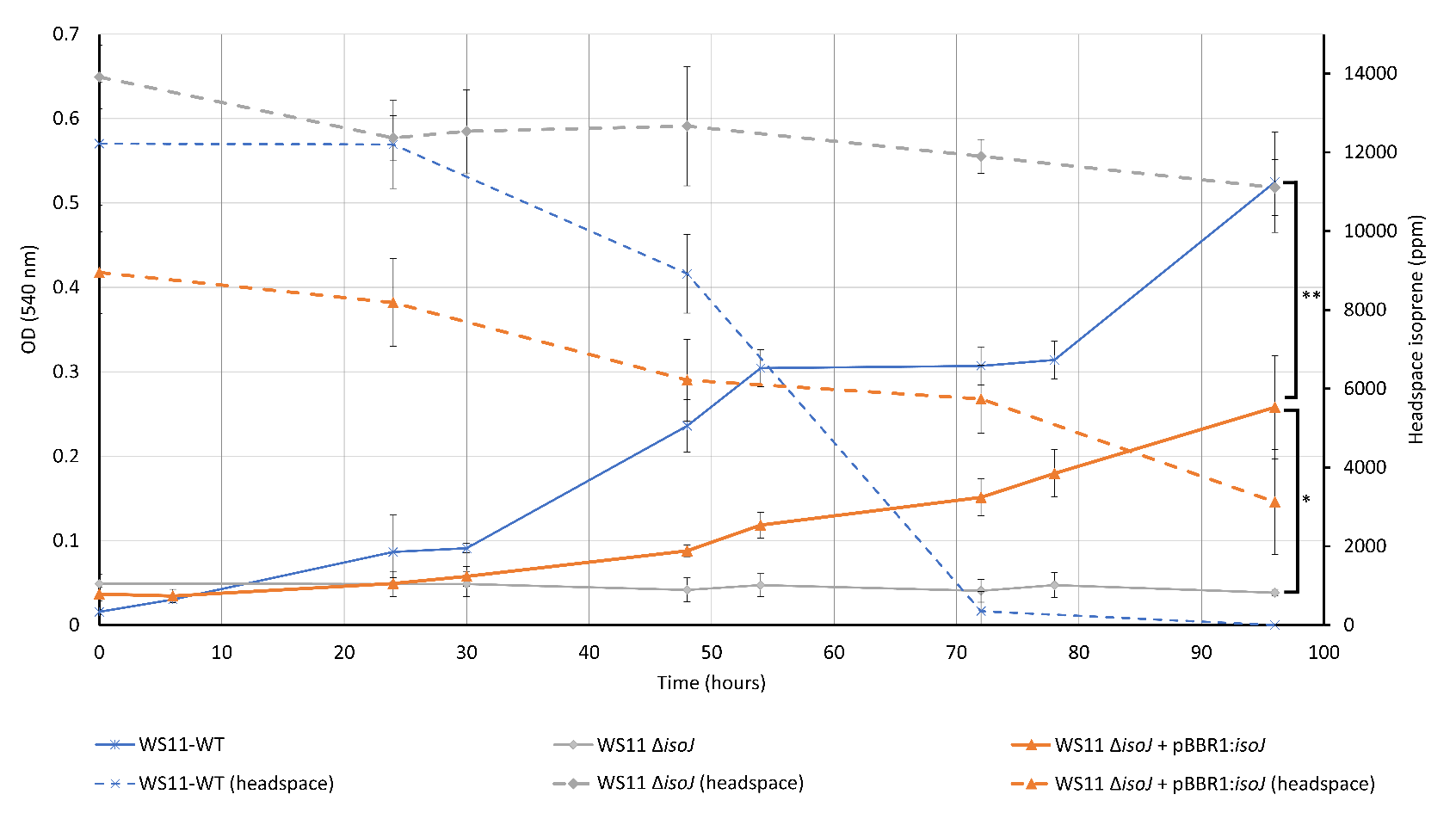


Figure S5. Growth of *Variovorax* sp. WS11 Δ*isoJ* on 1% (v/v) isoprene, compared with the growth of *Variovorax* sp. WS11 Δ*isoJ* transformed with pBBR1:*isoJ*. Error bars represent the standard deviation about the mean (n=3). An asterisk denotes a statistically significant difference between the indicated conditions (*p ≤ 0.05; **p ≤ 0.01).


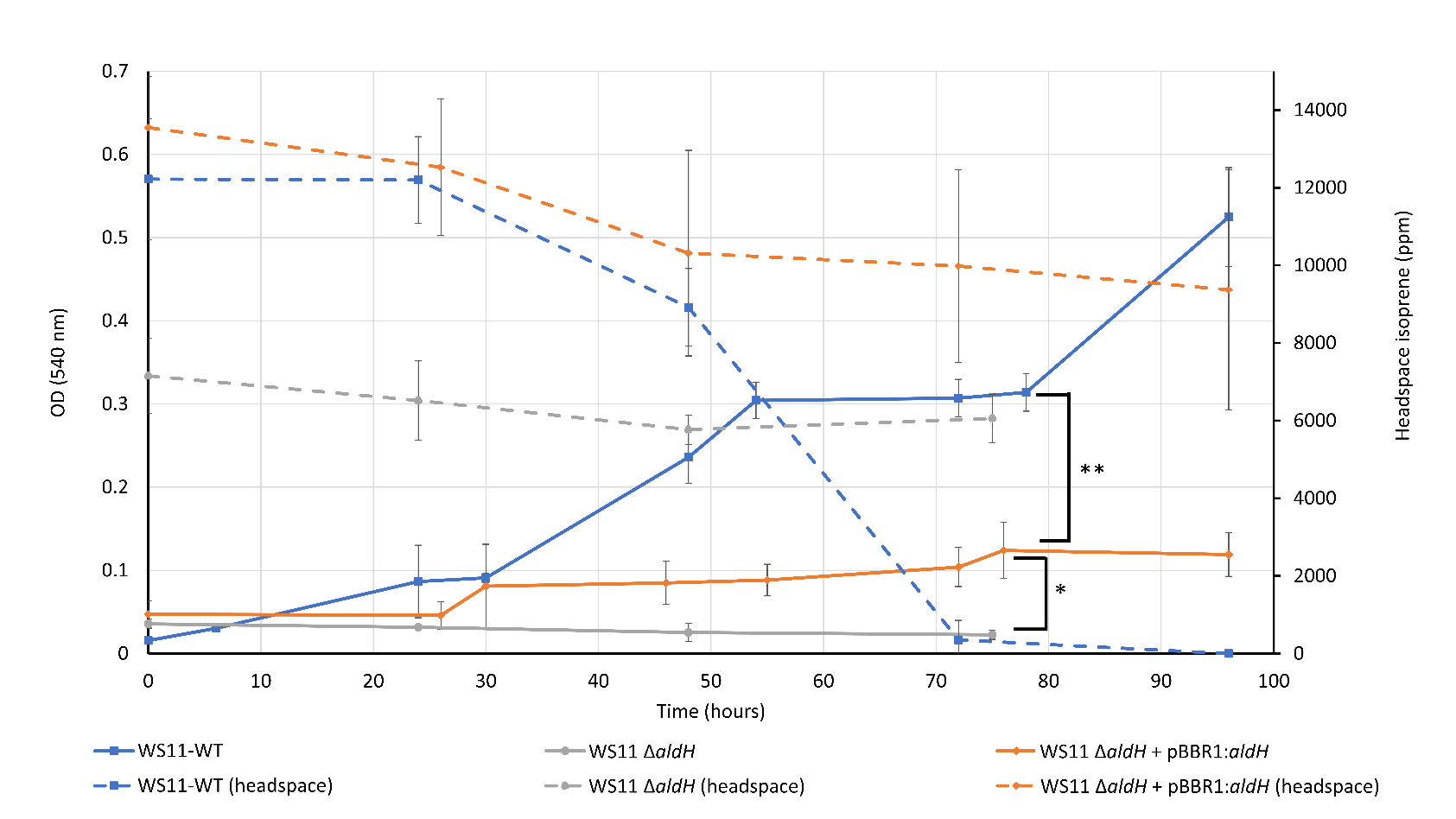


Figure S6. Growth of *Variovorax* sp. WS11 Δ*aldH* on 1% (v/v) isoprene, compared with the growth of *Variovorax* sp. WS11 Δ*aldH* transformed with pBBR1:*aldH*. Error bars represent the standard deviation about the mean (n=3). An asterisk denotes a statistically significant difference (*p ≤ 0.05; **p ≤ 0.01).


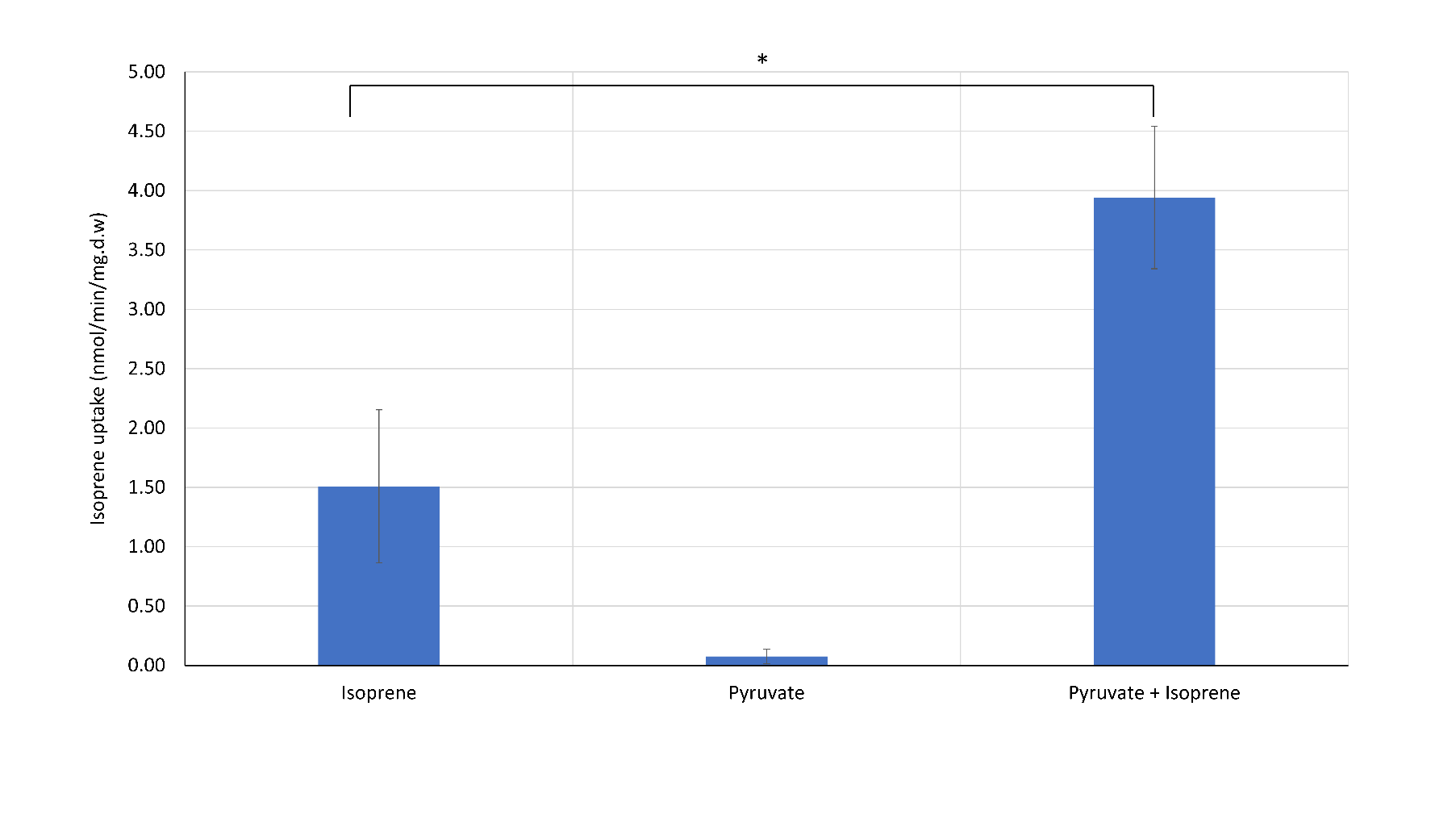


Figure S7. Isoprene oxidation by *Variovorax* sp. WS11 grown in the presence of 1% (v/v) isoprene, 10 mM pyruvate, or a combination of 1% (v/v) isoprene and 10 mM pyruvate, measured as nmol isoprene consumed minute^-1^ mg dry weight^-1^. Error bars represent the standard deviation about the mean (n=3). An asterisk denotes a statistically significant difference (p ≤ 0.05) between the indicated conditions, determined by students *t*-test.


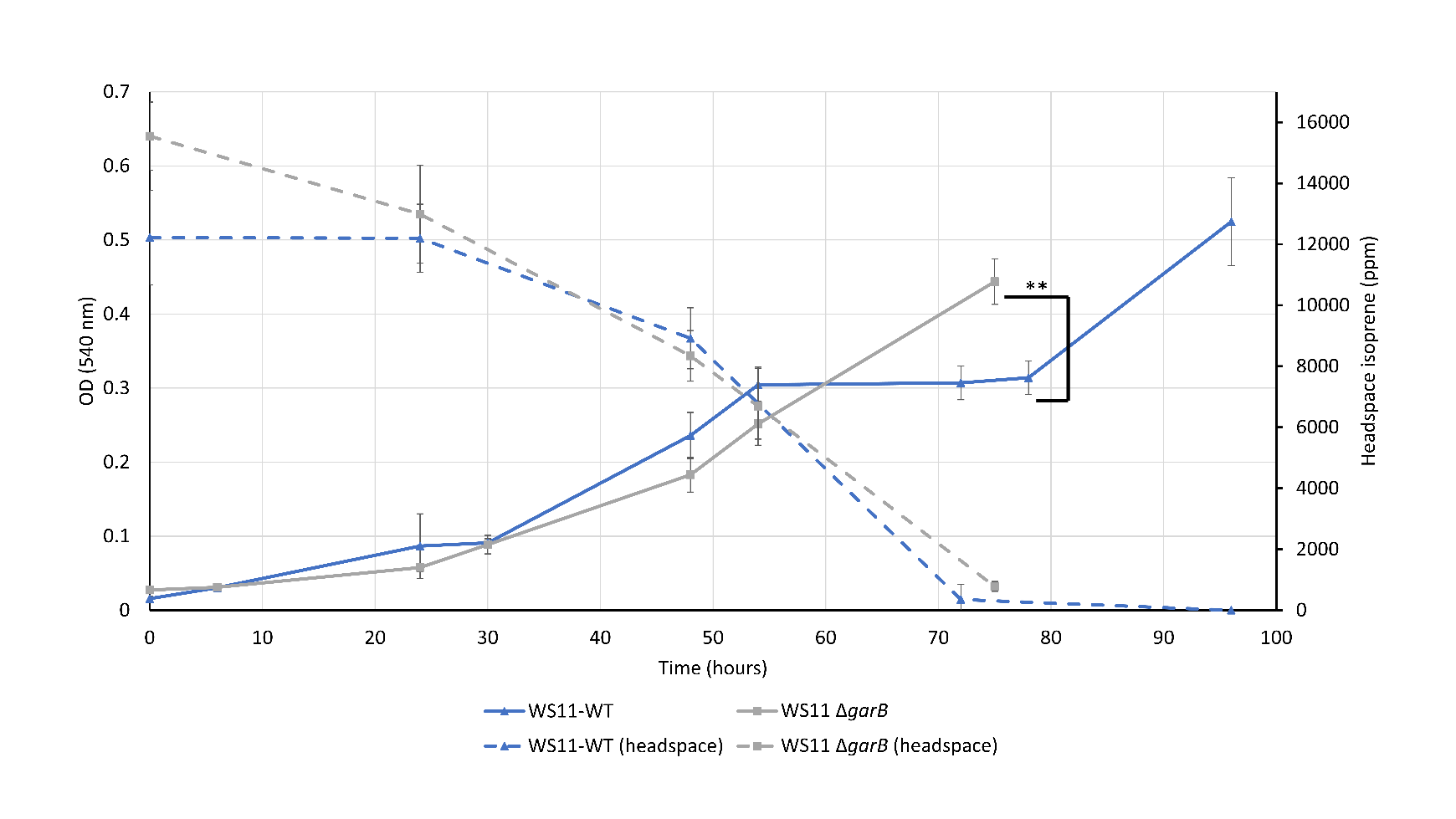


Figure S8. Growth of *Variovorax* sp. WS11 Δ*garB* on 1% (v/v) isoprene, compared to wild-type *Variovorax* sp. WS11. Error bars represent the standard deviation about the mean (n=3). An asterisk denotes a statistically significant difference between the indicated conditions (p ≤ 0.01).


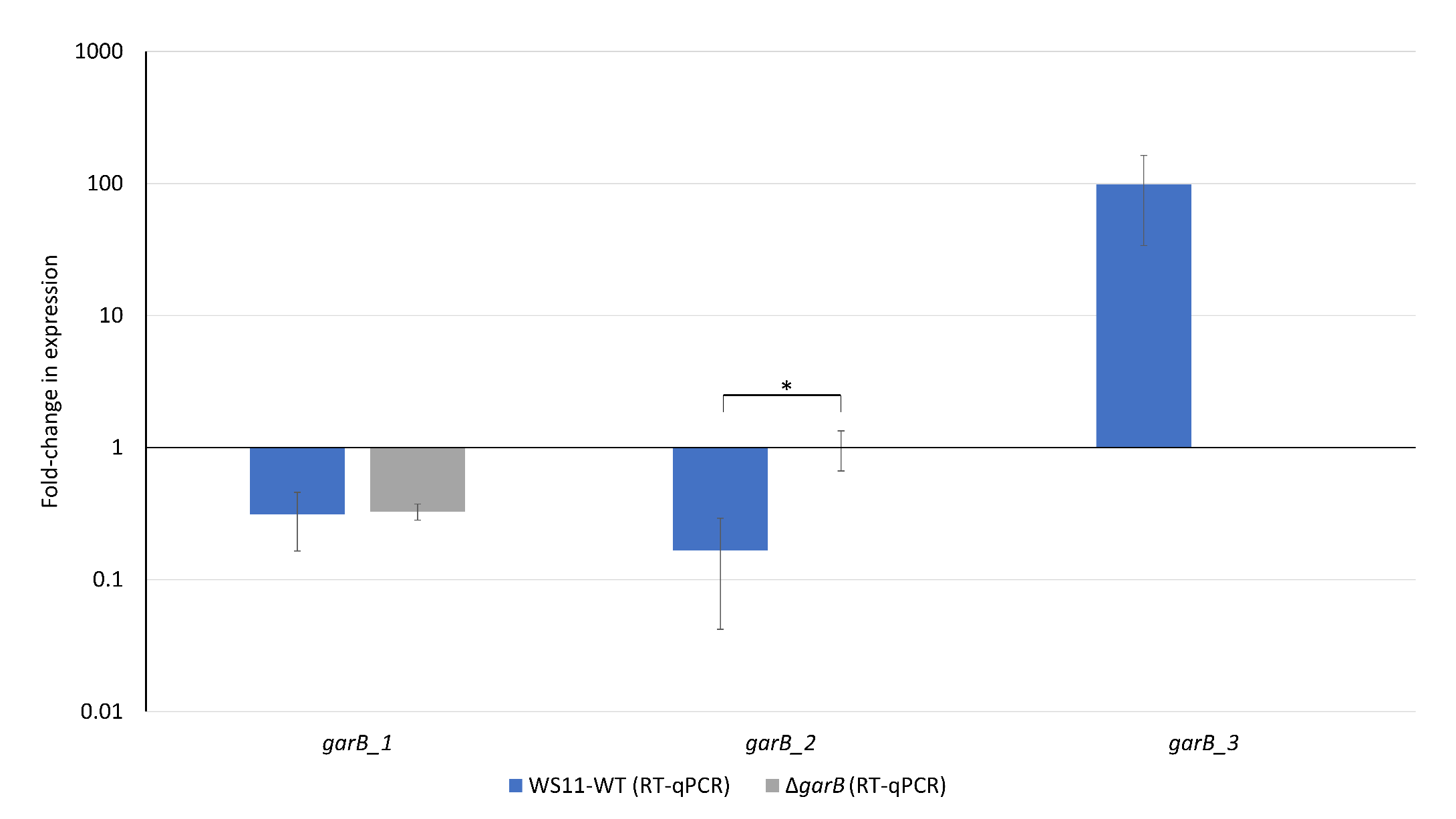


Figure S9. Fold-change in expression of *garB_1, garB_2*, and *garB_3* by *Variovorax* sp. WS11 (wild-type) and *Variovorax* sp. WS11 Δ*garB*, during growth on isoprene compared to growth on succinate, determined by RT-qPCR, relative to the expression of *rpoB*. The fold-change in expression of *garB_3* determined by RNA-seq was calculated relative to timepoint 0. Error bars represent the standard deviation about the mean (n=3). An asterisk (*) denotes a statistically significant difference between the indicated conditions (p ≤ 0.05).


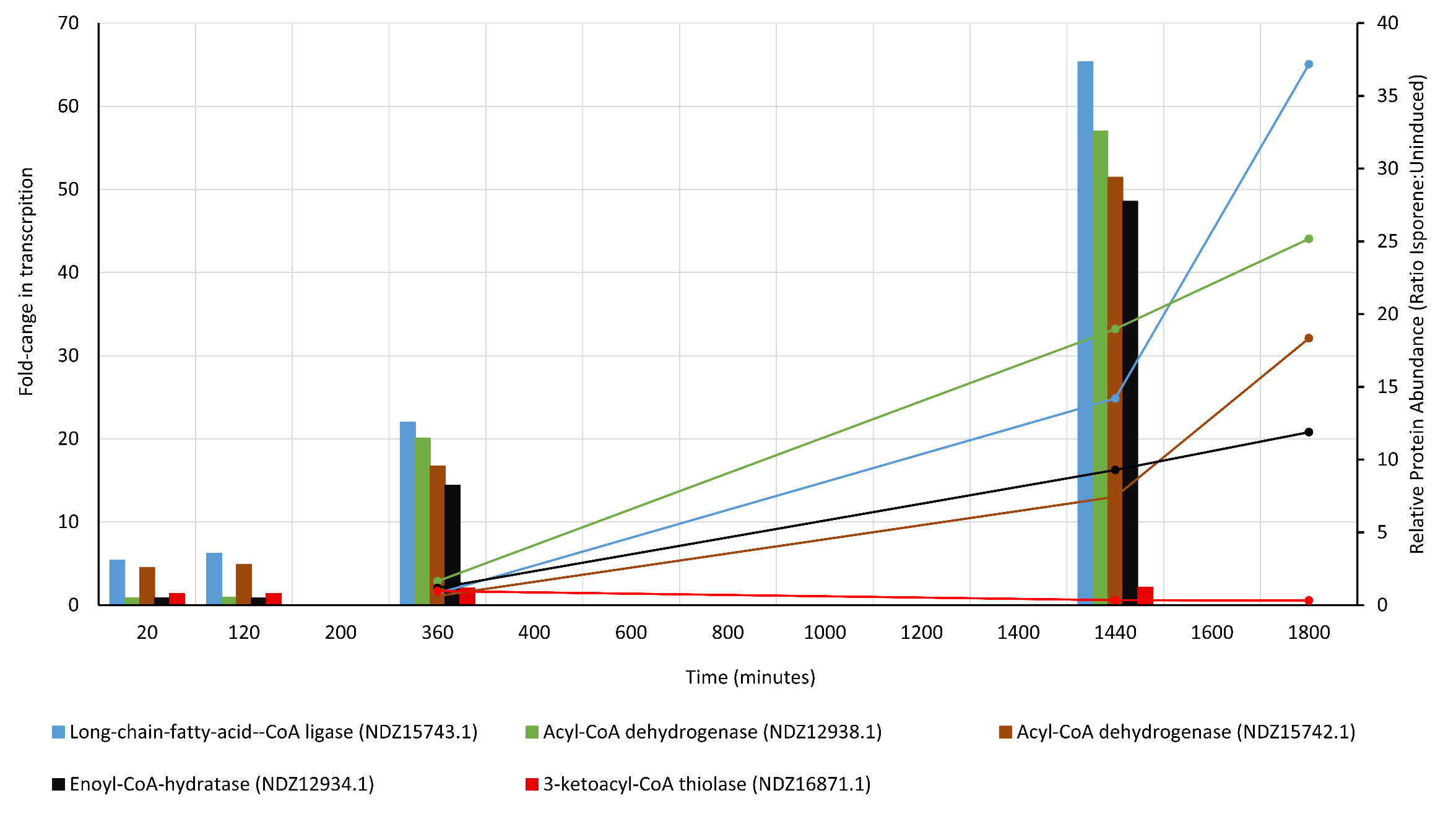


Figure S10. Isoprene-induced changes in the transcription of typical β-oxidation genes (bars), and the relative protein abundance of the respective gene products (lines).


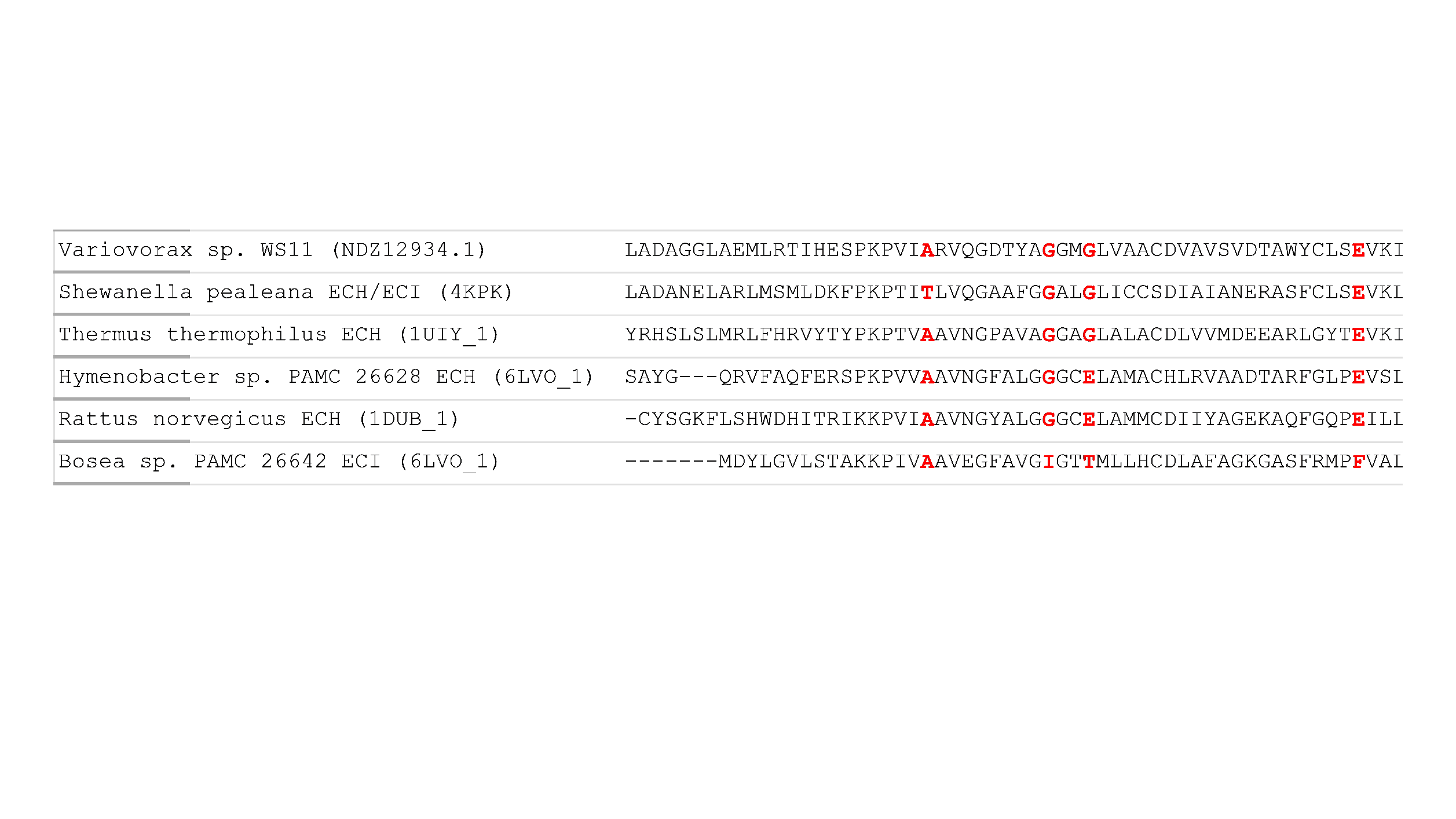


Figure S11: Alignment of the deduced amino acid sequence of NDZ12934.1 (ECH) with published ECH and ECI sequences, using the MAFFT online multiple sequence alignment tool with default parameters (Katoh *et al.*, 2019). Conserved residues (Ala98, Gly142, Glu144, Glu164, based on alignment against the *Rattus norvegicus* ECH (Padavattan *et al.*, 2021)) are highlighted in red. Glu144 has been substituted with a glycine residue in *Variovorax* sp. WS11. Where available, protein databank identifiers have been included in brackets.


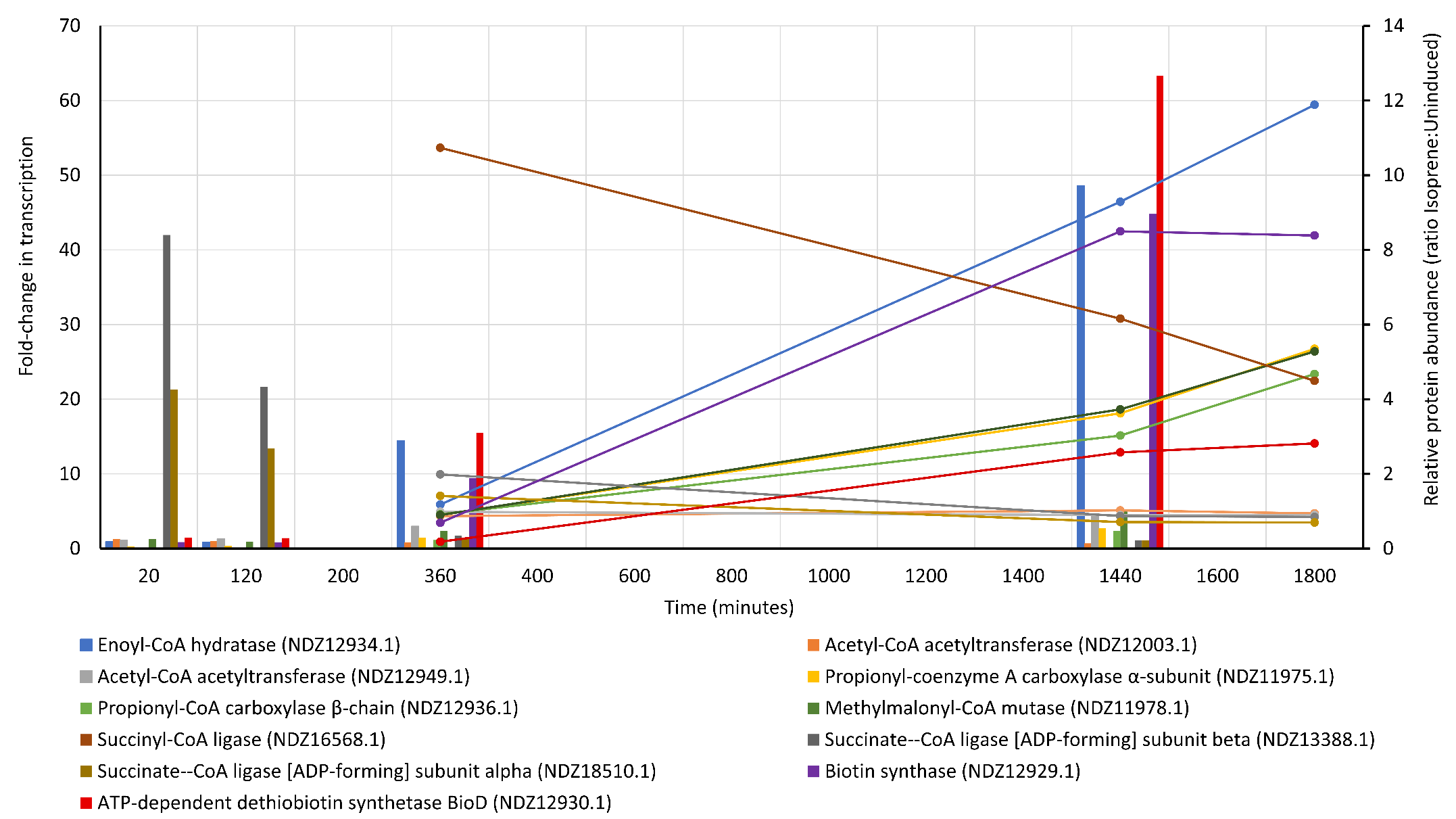


Figure S12: Isoprene-induced changes in the expression of transcribed genes (bars) and translated gene products (lines) with predicted roles in the isoprene metabolic pathway (Figure 4), subsequent to GMBA formation by IsoH.


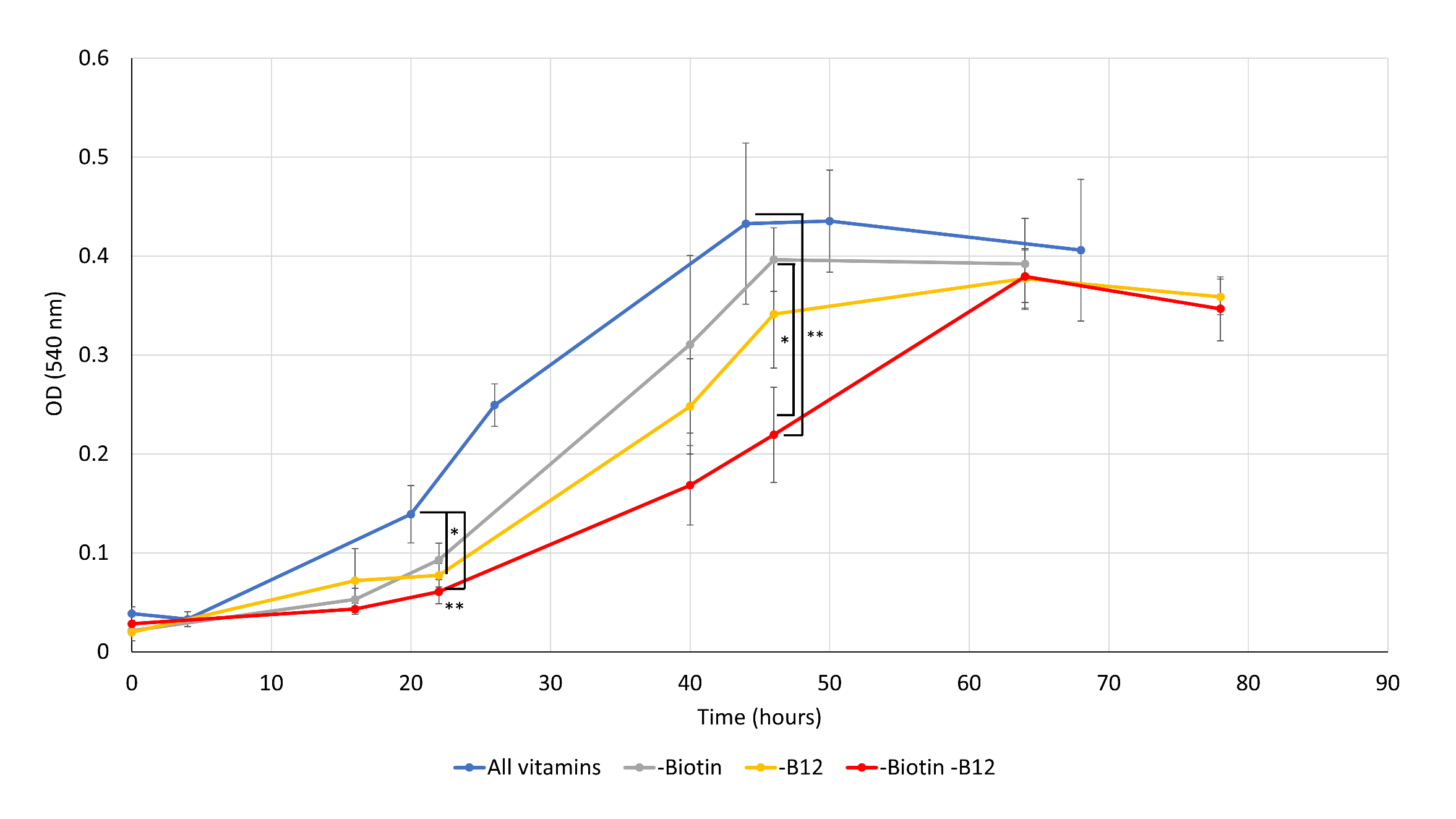


Figure S13. Growth of *Variovorax* sp. WS11 on 1% (v/v) isoprene in the presence of a complete vitamin solution, in the absence of biotin (-biotin), in the absence of vitamin B12 (-B12), or in the absence of both biotin and vitamin B12 (-Biotin -B12). Error bars represent the standard deviation about the mean (n=3). An asterisk denotes a statistically significant difference between the indicated conditions (* p ≤ 0.05, ** p ≤ 0.01).


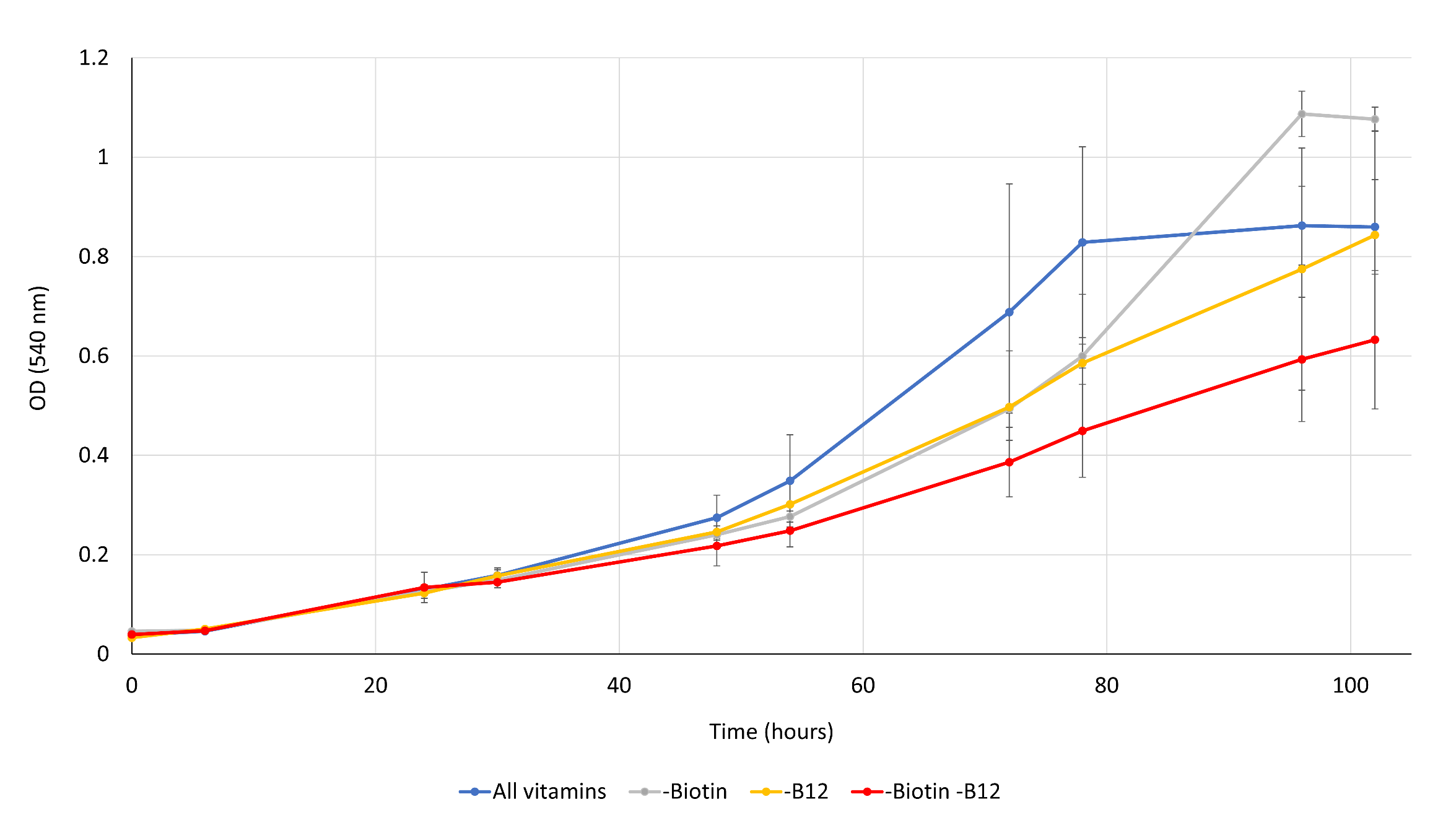


Figure S14. Growth of *Variovorax* sp. WS11 on 10 mM propionate in the presence of all vitamins, in the absence of biotin (-biotin), in the absence of vitamin B12 (-B12), or in the absence of both biotin and vitamin B12 (-Biotin -B12). Error bars represent the standard deviation about the mean (n=3).

Table S1. Top 30 most abundant transcripts and polypeptides after 24 hours’ growth on isoprene, calculated as the fold-change in transcripts and ratio of abundance of polypeptides, compared to the respective timepoint 0 samples.


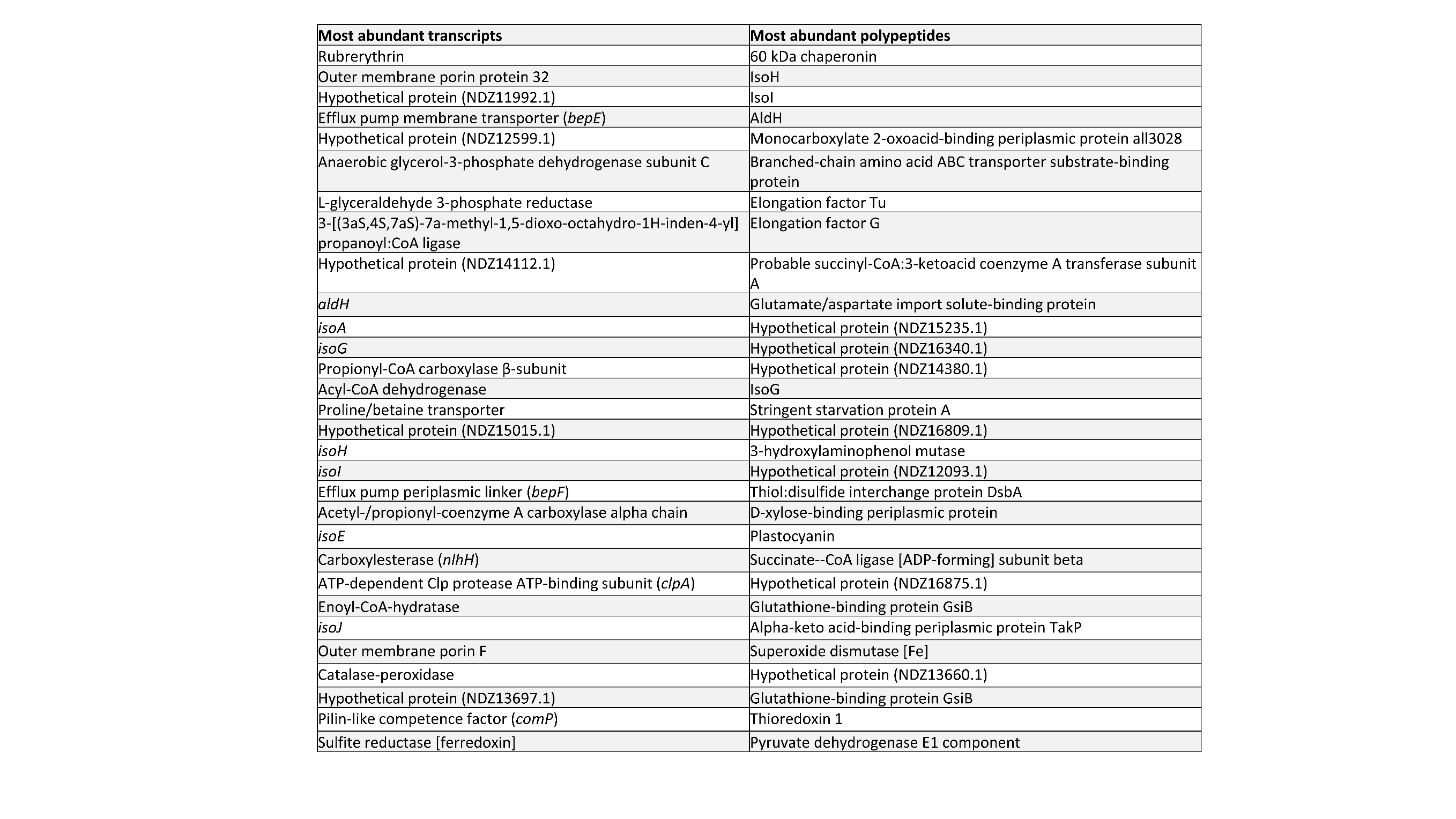


The relative abundance of proteins in Table S2 ranged from 1000 to 0.61 after 24 hours of growth on isoprene, relative to timepoint 0. The fold-change in transcripts in Table S2 ranged from 0.4 to 451.9 after 24 hours of growth on isoprene, relative to timepoint 0.

Table S2. Top 30 most upregulated transcripts and polypeptides after 24 hours’ growth on isoprene, calculated as the fold-change in transcripts and the ratio of abundance of polypeptides, compared to the respective timepoint 0 samples.


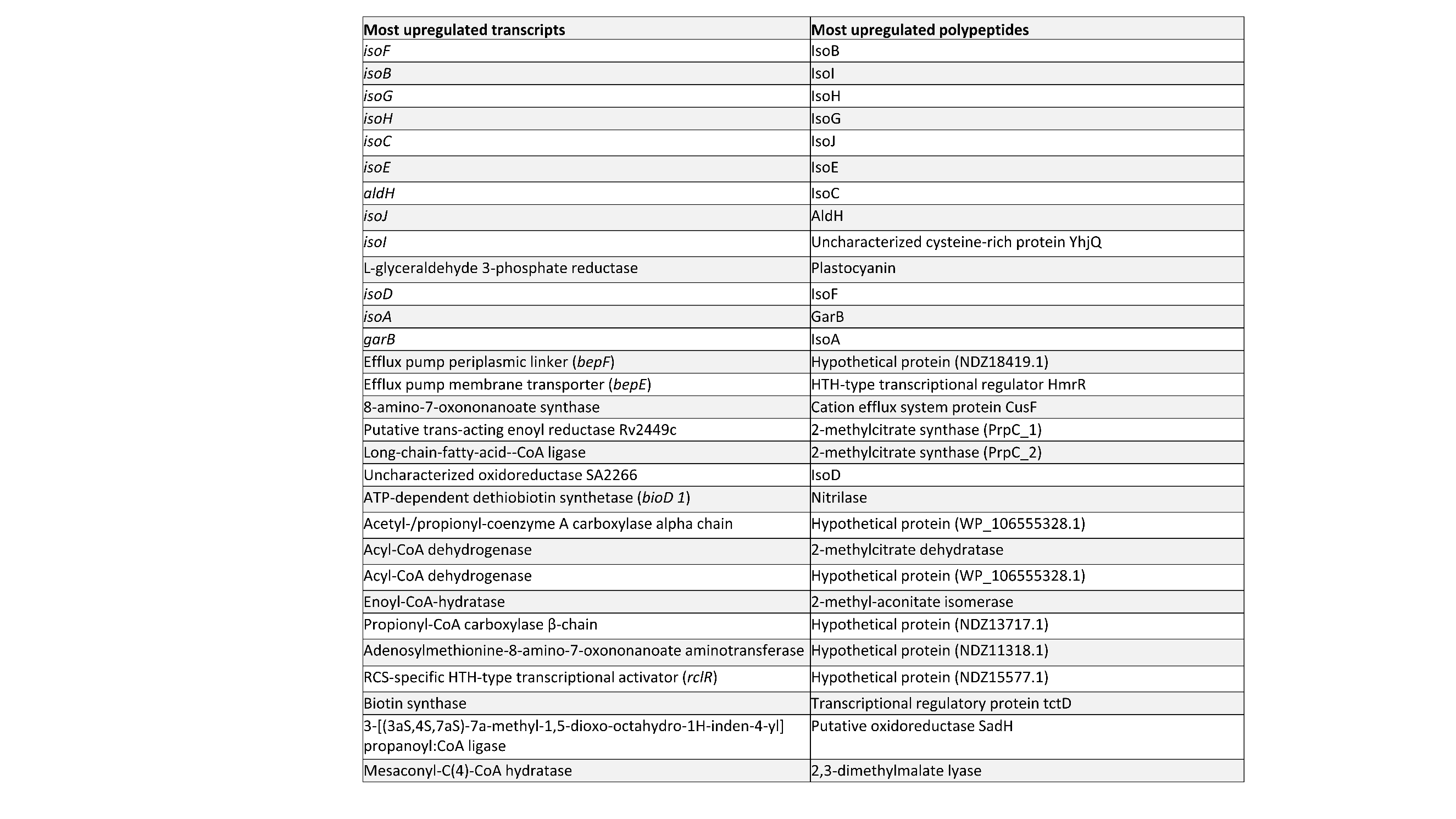


The relative abundance of proteins shown in Table 1 after 24 hours of growth on isoprene ranged from 1000 to 23.79, relative to timepoint 0. The fold-change in transcripts ranged from 41.4 to 643.6 after 24 hours of growth on isoprene, relative to timepoint 0. A single protein annotated in the proteome of isoprene-grown *Variovorax* sp. WS11 (WP_106555328.1) was identified as two genes in the genome of *Variovorax* sp. WS11 when annotated by GenBank (NZ_JAAGOW010000000), resulting in this entry appearing twice in Table S2.

Table S3. Presence (✓) or absence (X) of genes involved in the methylcitrate pathway and propionyl-CoA assimilation pathways in isoprene degrading bacteria.


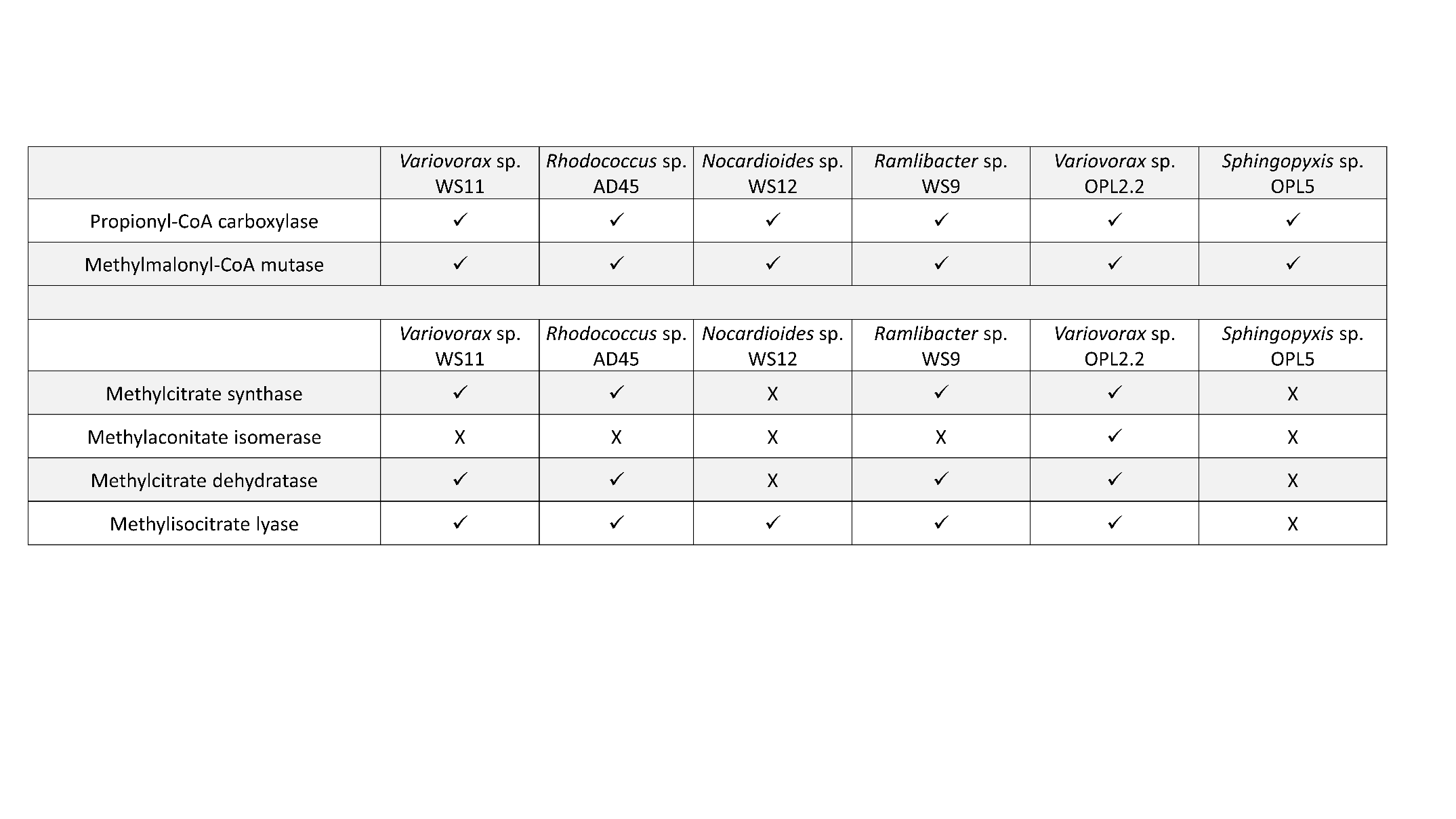


Table S4. Vectors used in this study.


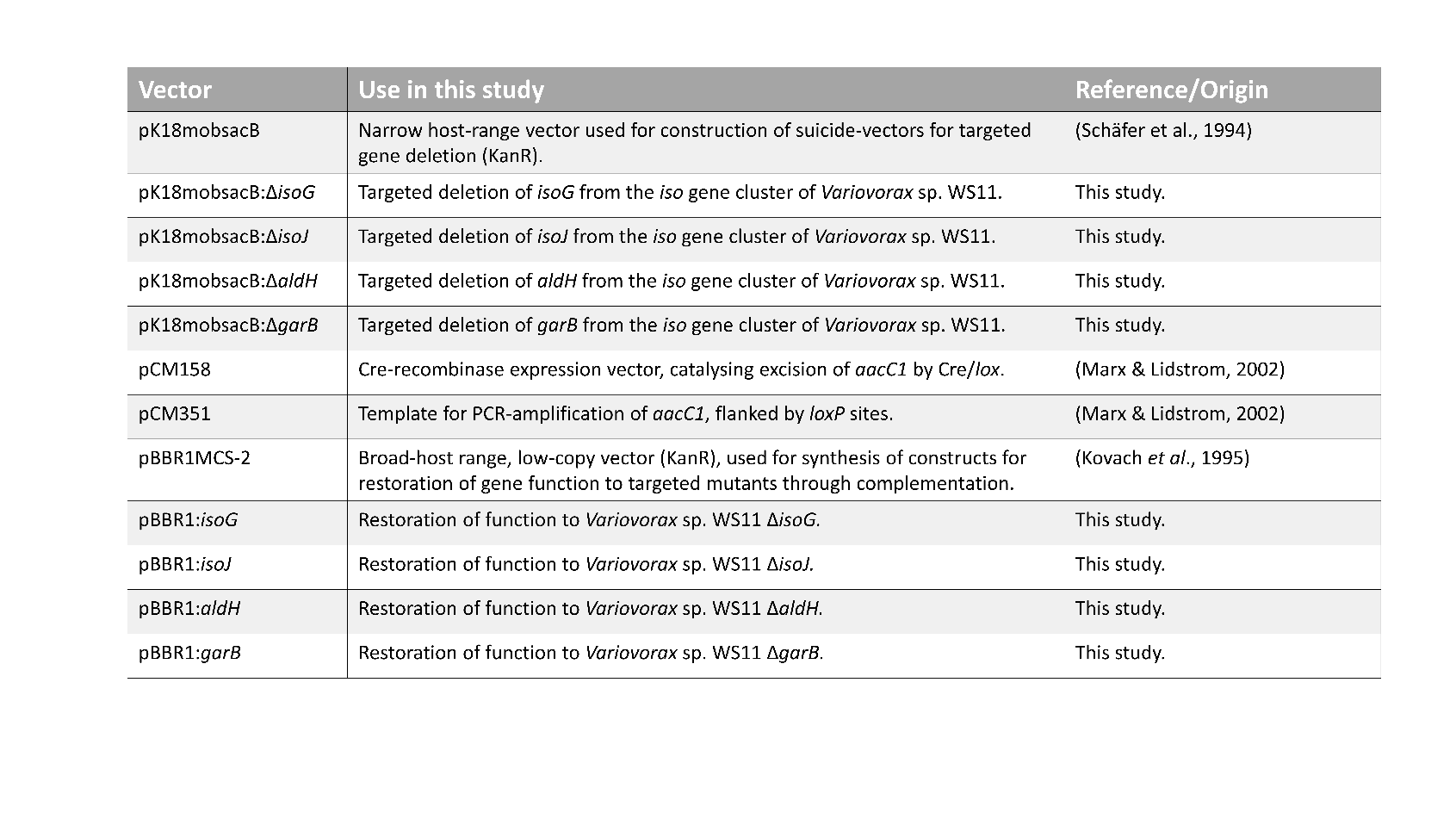


(Marx and Lidstrom, 2002)(Schäfer *et al.*, 1994; Kovach *et al.*, 1995)

Table S5. Primers used in this study.


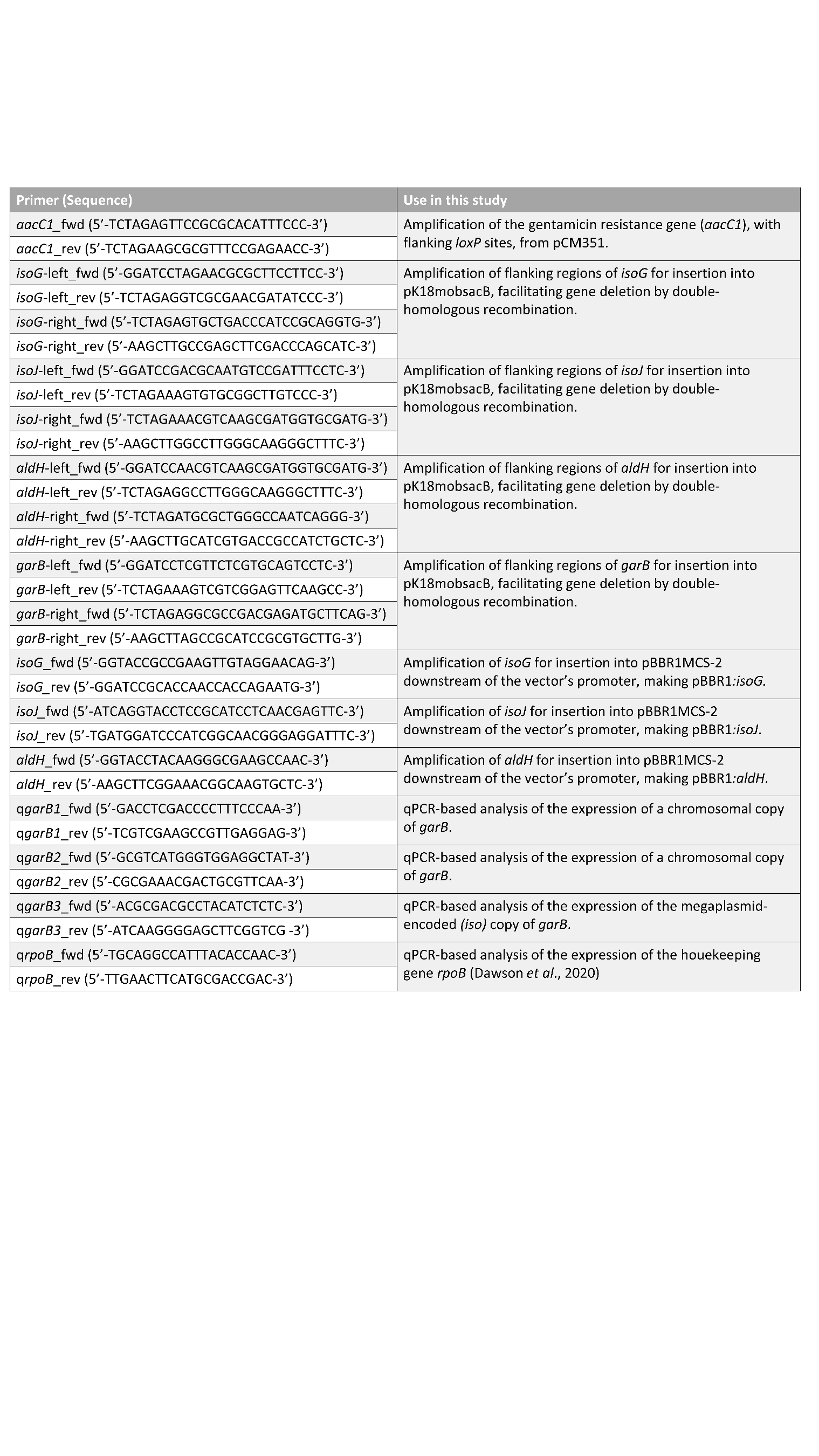


Supplementary references

Brenes, A., Hukelmann, J., Bensaddek, D., and Lamond, A.I. (2019) Multibatch TMT reveals false positives, batch effects and missing values. *Mol Cell Proteomics* **18**: 1967–1980.

Dawson, R.A., Larke-Mejía, N.L., Crombie, A.T., Farhan Ul Haque, M., and Murrell, J.C. (2020) Isoprene oxidation by the Gram-negative model bacterium *Variovorax* sp. WS11. *Microorganisms* **8**: doi: 10.3390/microorganisms8030349.

van Hylckama Vlieg, J.E.T., Leemhuis, H., Jeffrey, H., Spelberg, L., and Janssen, D.B. (2000) Characterization of the gene cluster involved in isoprene metabolism in *Rhodococcus* sp. strain AD45. *J Bacteriol* **182**: 1956–1963.

Katoh, K., Rozewicki, J., and Yamada, K.D. (2019) MAFFT online service: multiple sequence alignment, interactive sequence choice and visualization. *Brief Bioinform* **20**: 1160–1166.

Kovach, M.E., Elzer, P.H., Hill, D.S., Robertson, G.T., Farris, M.A., Roop, R.M., and Peterson, K.M. (1995) Four new derivatives of the broad-host-range cloning vector PBBR1MCS, carrying different antibiotic-resistance cassettes. *Gene* **166**: 175–176.

Lane, D.. (1991) 16S/23S rRNA sequencing. In *Nucleic Acid Techniques in Bacterial Systematics*. Stackebrant, E. and Goodfellow, M. (eds). New York: John Wiley & Sons, pp. 115–175.

Marx, C.J. and Lidstrom, M.E. (2002) Broad-host-range cre-lox system for antibiotic marker recycling in Gram-negative bacteria. *Biotechniques* **33**: 1062–1067.

Nickerson, J.L. and Doucette, A.A. (2020) Rapid and quantitative protein precipitation for proteome analysis by mass spectrometry. *J Proteome Res* **19**: 2035–2042.

Padavattan, S., Jos, S., Gogoi, H., and Bagautdinov, B. (2021) Crystal structure of enoyl-CoA hydratase from *Thermus thermophilus* HB8. *Acta Crystallogr Sect F Struct Biol Commun* **77**: 148–155.

Schäfer, A., Tauch, A., Jsger, W., Kalinowski, J.J., Thierbachb, G., Piihler, A., et al. (1994) Small mobilizable multi-purpose cloning vectors derived from the *Escherichia coli* plasmids pK18 and pK19: selection of defined deletions in the chromosome of *Corynebacterium glutamicum*. *Gene* **145**: 69–73.
